# Cortical Structure of Hallucal Metatarsals and Locomotor Adaptations in Hominoids

**DOI:** 10.1371/journal.pone.0117905

**Published:** 2015-01-30

**Authors:** Tea Jashashvili, Mark R. Dowdeswell, Renaud Lebrun, Kristian J. Carlson

**Affiliations:** 1 Evolutionary Studies Institute, University of the Witwatersrand, Wits, South Africa; 2 Department of Geology and Palaeontology, Georgian National Museum, Tbilisi, Georgia; 3 School of Statistics and Actuarial Science, University of the Witwatersrand, Wits, South Africa; 4 Institut des Sciences de l’Evolution de Montpellier—UMR 5554, Montpellier, France; 5 Department of Anthropology, Indiana University, Bloomington, Indiana, United States of America; Université de Lyon–Université Jean Monnet, FRANCE

## Abstract

Diaphyseal morphology of long bones, in part, reflects in vivo loads experienced during the lifetime of an individual. The first metatarsal, as a cornerstone structure of the foot, presumably expresses diaphyseal morphology that reflects loading history of the foot during stance phase of gait. Human feet differ substantially from those of other apes in terms of loading histories when comparing the path of the center of pressure during stance phase, which reflects different weight transfer mechanisms. Here we use a novel approach for quantifying continuous thickness and cross-sectional geometric properties of long bones in order to test explicit hypotheses about loading histories and diaphyseal structure of adult chimpanzee, gorilla, and human first metatarsals. For each hallucal metatarsal, 17 cross sections were extracted at regularly-spaced intervals (2.5% length) between 25% and 65% length. Cortical thickness in cross sections was measured in one degree radially-arranged increments, while second moments of area were measured about neutral axes also in one degree radially-arranged increments. Standardized thicknesses and second moments of area were visualized using false color maps, while penalized discriminant analyses were used to evaluate quantitative species differences. Humans systematically exhibit the thinnest diaphyseal cortices, yet the greatest diaphyseal rigidities, particularly in dorsoplantar regions. Shifts in orientation of maximum second moments of area along the diaphysis also distinguish human hallucal metatarsals from those of chimpanzees and gorillas. Diaphyseal structure reflects different loading regimes, often in predictable ways, with human versus non-human differences probably resulting both from the use of arboreal substrates by non-human apes and by differing spatial relationships between hallux position and orientation of the substrate reaction resultant during stance. The novel morphological approach employed in this study offers the potential for transformative insights into form-function relationships in additional long bones, including those of extinct organisms (e.g., fossils).

## Introduction

Humans exhibit a unique foot structure that is distinct from African ape foot structure [1–4]. The presence and unique form of longitudinal and transverse bony arches, midfoot stability (as opposed to midfoot mobility in the calcaneocuboid and cuboid-metatarsal joints), loss of an opposable hallux, and shortening of pedal phalanges are some of the skeletal features distinguishing human foot form [5–14]. Accompanying unique ligamentous structures (e.g., plantar aponeurosis and long plantar ligament) reinforce the bony arches, also distinguishing human foot design from that of other apes [11], [15–19]. Exactly when these unique features emerged within human evolutionary history is still vigorously debated, but the selective reasons for their differentiation (i.e., confer structural advantages during terrestrial bipedalism) are widely accepted [20–23].

Attempts to quantify internal stresses and joint motions within the foot have been rare [24– 25] due to the difficult nature of analyzing *in vivo* foot mechanics without disrupting normal function. Rather, much of the information on loading patterns experienced by the foot has been derived from *in vitro* cadaver studies [26–27] or studies of external pressure and foot kinematics [8], [28–30]. That the human hallux functions comparatively better as a stable lever in a metatarsi-fulcrimating foot is likely a consequence of selection favoring a more medial and stereotyped path of the center of pressure during human bipedalism compared to the more lateral and variable path of the center of pressure observed during *Pan* terrestrial bipedalism or quadrupedalism [8], [28–30]. This difference is accentuated even more by speed (i.e., during higher pressures) since the center of pressure in the human foot follows an even more medial trajectory at faster speeds [11]. The comparatively more medial position of the center of pressure during stance would facilitate more close-packing of joints comprising the human bony longitudinal arch and elastic energy return from its associated ligamentous structures such as the plantar aponeurosis [11], [15], [17–18].

A comparatively robust and adducted human hallux is thought to reflect a unique human toe-off mechanism during terminal stance phase of bipedal gaits [6], [28], [31], whereas the forefoot configuration of other hominoids, particularly their less robust and abducted first ray, is thought to reflect a design for accommodating retained grasping capabilities [32]. Indeed, these different configurations result in the functional comparability of dorsoplantar cortices of human hallucal metatarsals and ML cortices in chimpanzee hallucal metatarsals due to internal rotation of the abducted hallux in the grasping foot [33– 34]. Greater midshaft robusticity of the human hallucal metatarsal compared to those of other apes [34–35] would result in the former being better-suited to resist comparatively higher loading (bending) experienced during the last half of stance ending with toe-off. Moreover, since the path of the center of pressure during human stance remains comparatively medial and thus more parallel to the longitudinal axis of the hallucal metatarsal from initial contact between the forefoot and the ground at foot flat (i.e., the point when pressure initially appears below the hallucal metatarsal head) until the heel lifts and toe-off occurs [11], [29], bending experienced by the human hallucal metatarsal diaphysis presumably occurs predominantly in a dorsoplantar plane. Bending experienced by the diaphysis of hallucal metatarsals in chimpanzees and gorillas presumably is less stereotyped in the functionally equivalent dorsoplantar direction because of the observed variation in hallux position and the center of pressure during stance. The goal of the present study, therefore, is to document structural variation in diaphyses of hominoid first metatarsals using continuous cortical thickness distribution and selected cross-sectional geometric (CSG) properties in order to test predictions of form generated from observed loading histories during stance experienced by hominoid feet.

Modeling a long bone as a hollow beam permits using CSG properties to estimate the ability of its diaphysis to resist (bending) deformations that may occur during different activities, particularly dynamic activities such as locomotion [36–43]. Specifically, CSG properties quantify different aspects of form: cortical bone area (CA) provides information on axial compressive stiffness; second moments of area (SMAs) provide information on diaphyseal resistance to bending (i.e., stiffness) in specific directions (i.e., about specific neutral axes); and the polar moment of area (J) provides information on twice average bending stiffness of a diaphysis. Most CSG studies focus attention on limb segments that contain only a single bone (e.g., humerus or femur) because form-function relationships are more easily inferred than those in limb segments containing paired bones, such as the forearm or leg [44–48]. Studies of CSGs from elements of the primate hand (e.g., metacarpals) or foot (e.g., metatarsals) are comparatively rarer [34–35], [49–51]. Since hands and feet of weight-bearing limbs generally make direct contact with the substrate during locomotion, it is reasonable to generalize that metacarpal and metatarsal diaphyses more straightforwardly reflect loading patterns compared to diaphyses of bones in more proximal limb segments (e.g., femora). In other words, since limb segments are more aligned with ground reaction resultants due to the proximity in contact, bones of the hands and feet experience comparatively smaller bending moments. However, it is important to remain mindful of the complexities in correspondence between CSGs and actual loading regimes that make inferring form-function relationships challenging, irrespective of the bone being examined [52–55].

Marchi [34] compared hominoid metatarsal midshaft structure using characterizations of habitual locomotor repertoire differences. Marchi [34] standardized midshaft CA to estimated body mass, while J was standardized to the product of estimated body mass and bone length. Marchi [34] Table 4 reported the highest size-standardized midshaft CAs in chimpanzee hallucal metatarsals and the lowest in gorillas, while humans and orangutans were intermediate. Human hallucal metatarsals, on the other hand, exhibited the highest size-standardized midshaft polar moments of area followed by chimpanzees, gorillas, and finally orangutans. Specifically, humans exhibited the highest midshaft Js in marginal metatarsals (i.e., first and fifth metatarsals), while other hominoid groups usually displayed progressively lower midshaft Js from medial to lateral metatarsals. The distinctive human pattern in metatarsal robusticity observed by Marchi [34] corroborated earlier findings by Day and Napier [6] and Reisenfeld [56] but see [57–58].

Recently, advanced methods of visualizing cortical bone thickness distributions and structural parameters of femoral diaphyses, such as SMAs and section moduli, have been demonstrated [59–62]. These methods, which focus on using false color maps to display continuous structure, offer exciting opportunities to comprehensively characterize long bone diaphyses. For example, continuous cortical thickness and stiffness distributions within a cross section can be visualized rather than restricting the extraction of structural information to a handful of parameters [e.g., rigidities in anatomical anteroposterior (AP) and mediolateral (ML) orientations]. In a recent study by Bondioli and colleagues [61], cortical bone thickness distributions of two femoral diaphyses (i.e., from a recent human and a Late Upper Paleolithic human) were qualitatively compared by visualizing patterns in thicknesses using false color maps. Subsequently, in a series of studies, Morimoto and colleagues [59], [63–65] extracted and visualized structural properties of hominoid femoral diaphyses from non-adult and adult, or captive and non-captive individuals, focusing on form comparisons and phyletic signals. In addition to nicely visualizing local diaphyseal structure at the site of muscle attachments [65], [59], Morimoto and colleagues [59] quantified cortical bone distribution patterns by employing principal component analyses (PCA) to explain variability in femoral cortical thickness, second moment of area, and section modulus. Not until the study by Puymerail and colleagues [60], however, was an appreciation of the loading regime of the femur fully integrated with this type of visualization approach. Puymerail and colleagues [60] extracted and visualized cortical bone distributions in an Indonesian *Homo erectus* partial femur (Kresna 11) by mapping cortical thickness to a false color spectrum. They depicted %CA and section modulus ratios (Z_x_/Z_y_) at 1% intervals along the diaphysis of the hominin femur, subsequently comparing analogous properties from a number of modern human femoral diaphyses in order to qualitatively evaluate structure of the Kresna 11 partial femur. Puymerail and colleagues [60] noted that the *H*. *erectus* femur exhibited a diaphysis with relatively greater distal thickening (greater %CA), and more ML stiffness (reinforcement) throughout its proximal two-thirds compared to diaphyses of modern human femora, suggesting this reflected greater mobility and different body proportions (i.e., breadths). While these studies collectively demonstrate the potential for extracting substantially more structural information than traditional applications of CSGs [54], which tend to focus more narrowly on structural information from far fewer cross sections per diaphysis (but see [66]), thus far they have been slow to fully integrate loading histories, possibly because of the complexity of the femoral loading regime.

Further refinement of quantifying continuous cortical thickness distributions using this approach is an additional goal of the present study. Extracting structural information from an image, such as topographical or thickness information [59–61], usually results in a data set where, *p*, the number of variables is more than, *n*, the number of sample members [e.g., each variable (pixel in a color map) is a thickness measurement in a contiguous series of radially-arranged thickness measurements in a cross section]. This statistical concern can be summarized as the “large *p*, small *n*” (“*p* >> *n*”) problem [67]. Furthermore, due to their spatial relationships, variables (pixels in a color map) originating from the discretization of a continuous signal in a cross section (e.g., adjacent thickness measurements) would be expected to be autocorrelated [68]. While several studies [59–61] clearly demonstrate the exciting potential for extracting new types of information from continuous measurements of cortical bone thickness and CSG properties (i.e., visualizing them), only one study has attempted to quantify the visualized patterns. Specifically, Morimoto and colleagues [59] used PCAs in an attempt to reduce the dimension of their data set. It should not be taken as a certainty, however, that a low-dimensional PCA projection (e.g., consideration of the first two or three components) will adequately separate comparative groups. Rather, it is feasible that all information relevant to group separation may be contained within the ultimate, penultimate, or last several principal components, even though the first few principal components capably explain substantial variation within the cumulative sample. Moreover, a PCA is not designed to resolve the problem of differentiating between comparative groups using data with a highly correlated “*p* >> *n*” structure, as outlined above [69]. Alternatively, a linear discriminant analysis is a more appropriate statistical procedure for quantifying data of this nature since it is designed to discriminate between known subgroups in a sample, and furthermore a penalized discriminant analysis (PDA) is especially designed to address problems associated with “*p* >> *n*” and autocorrelation, as described above [68–69].

In the present study, we aim to visualize and quantify cortical bone thicknesses and SMAs in a sample of hominoid hallucal metatarsals. We assess correspondence between predicted and observed diaphyseal structure in the context of documented foot loading patterns. We use a PDA, which we argue to be a more appropriate statistical procedure than a PCA given the nature of these data, to quantify group differences. Specifically, we compare three hominoid species, which represent two broadly different behavioural repertoires (e.g., human bipedalism versus non-human quadrupedalism), and that load their feet differently during terrestrial locomotion. In order to achieve these aims, we assess two specific hypotheses. First, hallucal metatarsal bone thickness (and other structural properties) should reflect species-specific patterns in foot loading during terrestrial gaits, such that humans are distinguishable from chimpanzees and gorillas in that the former emphasize dorsoplantar stiffness. Second, within each group, there are diaphyseal regions where low variability is expressed (i.e., regions where metatarsal thickness is relatively uniform across the sample), and these regions can be spatially distinguished from regions where high variability is expressed. A “low variability region approach” may highlight areas of the diaphysis within the overall sample, or within a specific subgroup, where bone distribution is highly constrained either for genetic (e.g., developmental canalization) or functional (e.g., low variability in loading regimes) reasons, versus regions that are highly variable perhaps for opposite reasons. Identifying constrained and/or variable regions in a continuous distribution of diaphyseal properties (e.g., thicknesses) may pinpoint areas that are particularly crucial (or irrelevant) for understanding structural similarities and differences in hominoids. In addressing these hypotheses, we provide new insights into form-function relationships in hominoid feet (i.e., hallucal metatarsals), and simultaneously advance the exciting potential for visualizing distributions of continuous thickness (and other structural properties) within long bone diaphyses.

## Materials and Methods

### Sample

Data were collected from hallucal metatarsals of 43 extant, adult hominoids (Table 1, S1 Text). Individuals were selected for inclusion in the study when postcranial skeletons were reasonably complete, and all foot bones were free of visible pathologies or injuries. The chosen sample includes right and left elements. Individuals in central chimpanzee (*Pan troglodytes troglodytes*) and western lowland gorilla (*Gorilla gorilla gorilla*) samples were wild shot adults (i.e., more precise ages are not available in museum records), and are housed in the Primate Collection of the Department of Comparative Anatomy of the National Museum of Natural History, Paris, France (S1 Text, S1 File). Humans (*Homo sapiens*) are housed in the Raymond A. Dart Collection of Human Skeletons at the University of the Witwatersrand, Johannesburg, South Africa. The human sample includes adults primarily between the ages of 20 and 30 (with one 45 year old male) from different southern African populations (e.g., Sotho, Xhosa and Pondo) (S1 Text, S1 File) [70]. It is unlikely that normally-active human adults in this age range would exhibit age-related bone loss, such as cortical thinning [71]. While age-related bone loss has been documented in chimpanzees [72–73], the phenomenon is undocumented in gorillas. Typically, cortical thinning is a product of faster outward expansion of the endosteal border compared to the periosteal border [74]. None of the chimpanzee or gorilla hallucal metatarsals stood out as having unusually thin diaphyses (S1 Fig), suggesting that age-related bone loss was not acting as a bias in the sample.

**Table 1:** Sample composition.

Genus	Species	Subspecies	Age	Sex
Pan	troglodytes	troglodytes*	14 adult individuals (M3 erupted)	6 males; 8 females
Gorilla	gorilla	gorilla*	14 adult individuals (M3 erupted)	9 males; 5 females
Homo	*sapiens* ^#^		15 adult individuals (M3 erupted)	8 males; 7 females

*Primate Collection of the National Museum of Natural History, Paris, France;

^#^The Raymond A. Dart Collection of Human Skeletons at the University of the Witwatersrand, Johannesburg, South Africa.

### Computed Tomography (CT) scanning protocol

Chimpanzee and lowland gorilla hallucal metatarsals were CT scanned at the Pitié-Salpêtrière Hospital (Paris, France), in the Department of Radiology, using a Philips iCT256 medical scanner. Human hallucal metatarsals were CT scanned with a Philips Brilliance 16P medical CT scanner at the Charlotte Maxeke Johannesburg Academic Hospital (South Africa). As both hospitals provided access to a Philips medical CT scanner, a single protocol—Open SKULL U.H.R. 0.5 protocol (Philips Healthcare, Andover, MA, USA)—could be used for image acquisition. Relevant parameters included: 140 kVp, a tube current of 253 mAs, a slice thickness of 0.67 mm, and a reconstruction increment of 0.3 mm. Subsequent to the acquisition of all raw data, image data for a specimen were reconstructed as 16-bit DICOM images using a bone reconstruction algorithm (i.e., a “sharp” kernel). Image data were segmented and rendered using Avizo 6.3.1 software (Visualization Sciences Group, Mérignac cedex, France), and a segmentation threshold varying between-450 and-750 HU. Once rendered, digital versions of metatarsals were separated using the Label Field module in Avizo 6.3.1 software (Visualization Sciences Group, Mérignac cedex, France).

### Data collection protocol

**Fig 1:**
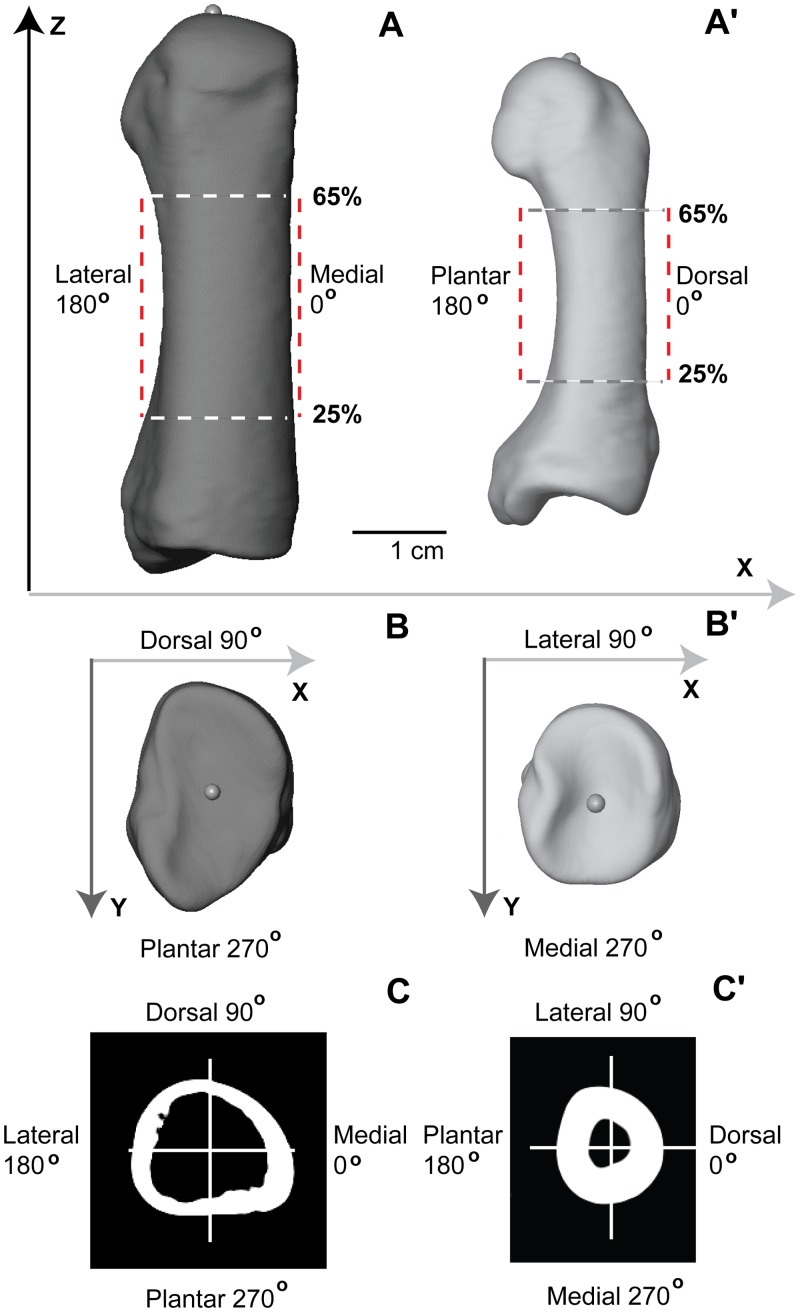
Protocol for positioning metatarsal renderings and extraction of cross sections. **A** Human (dorsal view) and **A’** Chimpanzee (lateral view): Landmarks positioned in centers of proximal (visible) and distal (not visible) articular surfaces were used to define the longitudinal axis of a diaphysis, which subsequently was aligned to the Z-axis in 3D space. Length refers to the distance between proximal and distal landmarks. Only the diaphyseal region between 25% and 65% length was investigated in the present analysis. Renderings (**A** and **A’**) illustrate broadly functionally equivalent positions of human and chimpanzee metatarsals during terrestrial locomotion, and thus the difference between functional and anatomical correspondence of cortices of the diaphysis. Specifically, and as explained in the method section (“Data Collection Protocol” subsection), the anatomical ML axis of chimpanzee (and gorilla) hallucal metatarsals is rotated internally 90 degrees, such that it becomes a ‘functional’ equivalent to the dorsoplantar axis of the human hallucal metatarsal. **B** Human and **B’** Chimpanzee (proximal views): After aligning the longitudinal axis of a rendering to the Z-axis in 3D space, and while viewing the proximal articular surface, each rendering was rotated about its longitudinal axis until the sides of the grooved proximal articular surface paralleled the Y-axis in 3D space. **C** Human and **C’** Chimpanzee: Cross section from 50% length of the rendering illustrated in **A** and 50% length of the rendering illustrated in **A’**. Note that in humans (**C**) 0 degrees corresponds to medial position on the diaphysis, 90 degrees to dorsal position, 180 degrees to lateral position, and 270 degrees to plantar position. In chimpanzees (and gorillas) (**C’**) 0 degrees corresponds to dorsal position on the diaphysis, 90 degrees to lateral position, 180 degrees to plantar position, and 270 degrees to medial position. Thus, functional comparisons between human and chimpanzee/gorilla hallucal metatarsal diaphyses compare the medial cortex in the former with the dorsal cortex in the latter, etc. See method section (“Data Collection Protocol” subsection) for additional explanation.

Mechanical length is the most relevant measure of length to use when modelling long bones as beams [38], [75]. For the sake of simplicity, in the remainder of the text we refer to this measure simply as length. Individual metatarsal renderings were carefully positioned in order to extract functionally comparable cortical thicknesses at regularly-spaced intervals (2.5%) from 25% to 65% length along the proximal-distal (longitudinal) axis of the shaft (Fig. 1A). Due to an approximately 90 degree internal rotation of the first ray in chimpanzees and gorillas resulting from their opposable hallux, the anatomically lateral cortex of chimpanzee and gorilla hallucal metatarsals is compared to the anatomically dorsal cortex of human hallucal metatarsals [33]. In the present study, following Marchi [34], a functional comparison was undertaken in order to evaluate hallucal metatarsal diaphyses amongst the groups. For example, when a superiorly-directed reaction force resultant is applied through the head of a hallucal metatarsal positioned in the sagittal plane, bending in its dorsoplantar axis (e.g., the human state) would be comparable to bending in the anatomical ML axis of an internally rotated metatarsal (e.g., the chimpanzee or gorilla state), or its ‘functional’ dorsoplantar axis. For ease of interpretation going forward and to maximize comparability with previous published work [34], the anatomical human dorsoplantar axis is compared to the ‘functional’ chimpanzee and gorilla dorsoplantar axis.

Protocol for positioning renderings was as follows: first, a landmark was positioned in the centre of proximal and distal articular surfaces; next, the rendering was rotated and translated in such a way that a line connecting the two landmarks became parallel to the Z-axis and orthogonal to the X-axis of the rendering (Fig. 1A); finally, each rendering was rotated about its Z-axis (Fig. 1B) following the protocol of Marchi [34]. In other words, after successfully applying the positioning protocol in 3D space, the landmark placed on the proximal articular surface was superimposed in the Z-axis on the landmark placed on the distal articular surface. After position was established, the original volume data corresponding to a rendering were resliced to reflect new spatial coordinates that took into account all previous translations and rotations. All right metatarsals were mirrored in order to approximate the form of (left) antimeres.

**Fig 2:**
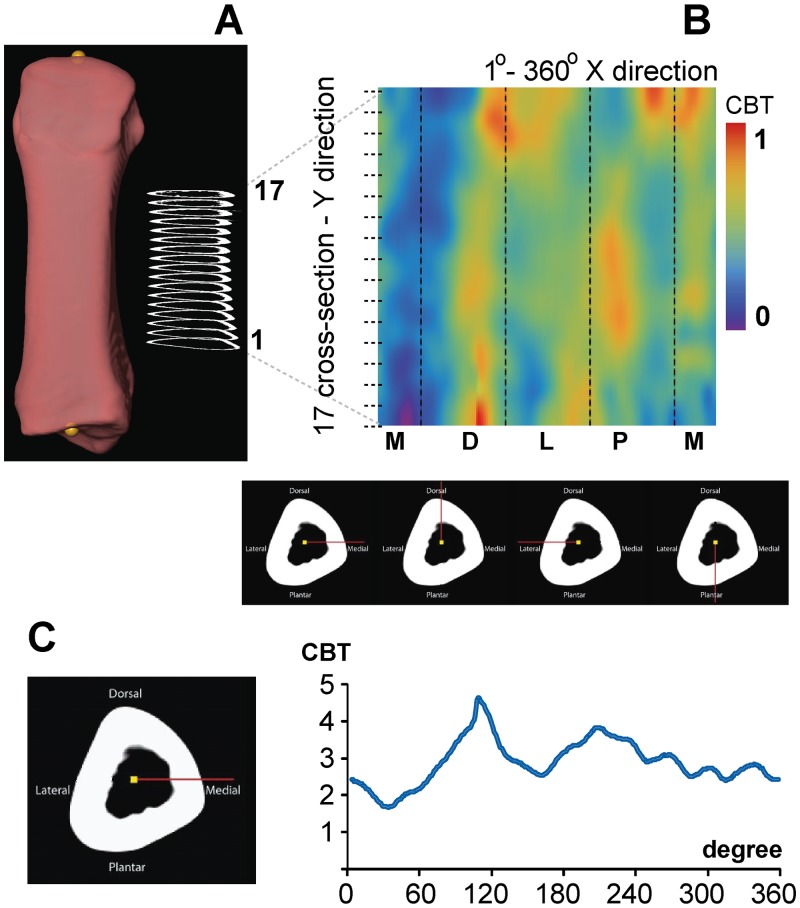
Quantifying and visualizing cortical bone thickness of hallucal metatarsals using false color maps. **A**: Rendering of a human hallucal metatarsal illustrated after application of the position protocol. A total of 17 cross sections were extracted from each metatarsal in 2.5% length increments beginning at 25% (1) and ending at 65% length. **B**: Cortical thicknesses in the 17 cross sections (vertical axis) were mapped to a continuous range of colours. Cortical thickness along each of the 360 rays of a cross section was visualized from left to right (horizontal axis) beginning at 0 degrees (medial: M), and continuing through 90 (dorsal: D), 180 (lateral: L), and 270 degrees (plantar: P). Purple-colored pixels represent minimum thickness, while red-colored pixels represent maximum thickness. Second moments of area were calculated about neutral axes using the same 360 degree incremental protocol. Below the color map, an example of a cross section (25% length) is provided with cortical thicknesses measured from the centroid (i.e., yellow circle) to the periosteal surface along red lines located at 0, 90, 180, and 270 degrees (left to right, bottom). **C**: Measurements of cortical thickness at each of the 360 rays (e.g., left) cumulatively generate an overall thickness profile for the cross section (right). Illustrated measurements are in mm and are not standardized.

Regions of interest (ROIs) containing specific diaphyseal cross sections were identified and extracted from positioned renderings (Fig. 1C). The resliced volume data (DICOM image stack) were cropped of empty images using proximal and distal landmarks as truncation points. Distance between the two landmarks in the Z-axis, therefore, was calculated and recorded as length. The proximal end corresponded to a landmark positioned at 0% length and the distal end corresponded to a landmark positioned at 100% length (Fig. 2A). Only the diaphyseal region between 25% and 65% length was analyzed in the present study. This avoided including regions of metatarsals where the presence of trabecular bone may have unduly influenced cortical bone properties (e.g., thickness). An interval between consecutive cross sections of 2.5% length was chosen in order to balance the tradeoff between oversampling (e.g., a desire to minimize autocorrelation within and between cross sections) and undersampling the diaphysis (e.g., a desire to capture useful structural information). The chosen interval (2.5%) corresponded to approximately 1.5–1.6 mm in actual length of typical metatarsal diaphyses. Consequently, 17 cross sections were extracted from each rendering and individually saved as 8-bit BMP files (Fig. 2A). Pixels in each image of the BMP stack were standardized to dimensions of 0.1 mm, after which images were resaved. This avoided introducing bias into the calculation of thicknesses and cross-sectional properties that could result from using different pixel dimensions (i.e., size-dependent field of view parameter). All steps described above were performed in Avizo 6.3.1 software (Visualization Sciences Group, Mérignac cedex, France).

Cortical bone thicknesses (CBTs), and second moments of area (SMAs) from each of the 17 cross sections comprising a rendering were quantified by one of us (MRD) using custom-written code in Wolfram Mathematica^©^9 (Wolfram Research, Inc., Mathematica, Version 9.0.1, Champaign IL). The initial step in the routine consisted of differentiating pixels containing material of interest from pixels containing background. Holes within cortex (e.g., passages for nutrient arteries) were filled. Boundary pixels were included or excluded using a bivariate normal kernel within the custom-written Mathematica code (S2 Text). Subsequently, structural properties (e.g., CBTs and SMAs) were calculated for each cross section in one degree increments (Fig. 2B) [76]. As with the selection of an optimal length interval (i.e., 2.5% length), and mindful of computational constraints, an increment of one degree was chosen in order to balance a trade-off between oversampling (e.g., motivated by capturing useful structural information) and undersampling diaphyses (e.g., motivated by minimizing autocorrelation within cross sections). Periosteal radius was calculated as the distance from the centroid of a given cross section to its intersection with the outermost border, while endosteal radius was calculated as the distance from the centroid of the given cross section to its intersection with the innermost border. Both were measured along the same ray. Each of the radial measurement pairs was obtained using one degree increments, proceeding counter-clockwise about an entire cross section (i.e., 720 measurements along 360 rays per cross section). Cortical thickness was calculated as the difference between paired periosteal and endosteal radii for each of the 360 rays in a cross section. SMAs were calculated about 360 neutral axes also oriented in successive one degree increments in a cross section. For example, in humans these started medially (i.e., neutral axes for rays 0 and 180 were parallel to the ML plane of a cross section) and proceeded counter-clockwise in a cross section. This resulted in unavoidable redundancy in SMA values, for example, neutral planes through rays 0 and 180, through rays 1 and 181, and so on. Incorporating this redundancy was useful, however, since it generated more intuitive color maps that could comprehensively visualize an entire cross section, or diaphysis.

As structural parameters of diaphyses (e.g., cortical areas and SMAs) are known to scale with body size and proportions [75], they were standardized before visualization and quantitative analyses. Cortical thickness measurements, which describe the distribution of material in a diaphysis, were standardized by length (i.e., as described above, the distance between proximal and distal landmarks along the Z-axis of a rendering), which served as a proxy for overall size. Second moments of area, which quantify bending stiffness in a direction perpendicular to their neutral axis, were standardized by the product of (estimated) body mass and length [47]. For chimpanzees and gorillas, sex-specific mean body mass reported for each species [77] was used. For humans, body mass was estimated using AP femoral head diameter from the right femur associated with a metatarsal. These measurements were inserted into sex-specific regression formulae [78] in order to obtain individual body mass estimates. From the individual estimated body masses, human sex-specific means were generated and used in standardizing properties. These were used in order to be consistent with the use of chimpanzee and gorilla sex-specific body masses.

### Color Map Visualization

Recent applications have demonstrated the usefulness of morphometric color maps in visualizing morphological differences between human, hominin, and ape femora [59], [61]. Similarly, we apply a 2D color map approach in visualizing morphological differences between hominoid hallucal metatarsals. Each 2D color map was generated using Wolfram Mathematica^©^9 (Wolfram Research, Inc., Mathematica, Version 9.0.1, Champaign IL). The horizontal axis of each color map represents consecutive radii circumnavigating a cross section (Fig. 2A), for example, where the medial location on each human hallucal metatarsal rendering indicates the starting point of the horizontal axis (0°). Thus, the dorsal position in human hallucal metatarsals occurs at 90° counter-clockwise, the lateral position at 180°, and the plantar position at 270°. In other words, the horizontal axis visualizes the medial cortex and precedes counter-clockwise 360 degrees around the diaphyseal cross section of human hallucal metatarsals, as viewed from the proximal end of the diaphysis. The vertical axis of each color map represents cross section number (e.g., percentage length), whereby the bottom-most pixels in a map are from the most proximal cross section (1) and the top-most pixels in a map are from the most distal cross section (17) (Fig. 2A). In this manner, standardized structural variables of a diaphysis (e.g., standardized CBTs and SMAs) can be visualized using a series of binned pixels comprising a color map. Ultimately, the location of each pixel corresponds to a specific radial position in a given cross section of the diaphysis, and the color of each pixel corresponds to a specific value in the binned range (Fig. 2A-B). In order to avoid a stepping artefact when visualizing vertical and horizontal axes of color maps (i.e., stepping with a frequency dictated by the number of cross sections or the number of radii about a cross section; a matrix of 17 rows by 360 columns), pixels from successive rays within a cross section or from successive cross sections were linked and values resampled for visualization purposes using the default resampling scheme in Mathematica’s “ImageResize” function (Wolfram Research, Inc., Mathematica, Version 9.0.1, Champaign IL).

**Fig 3:**
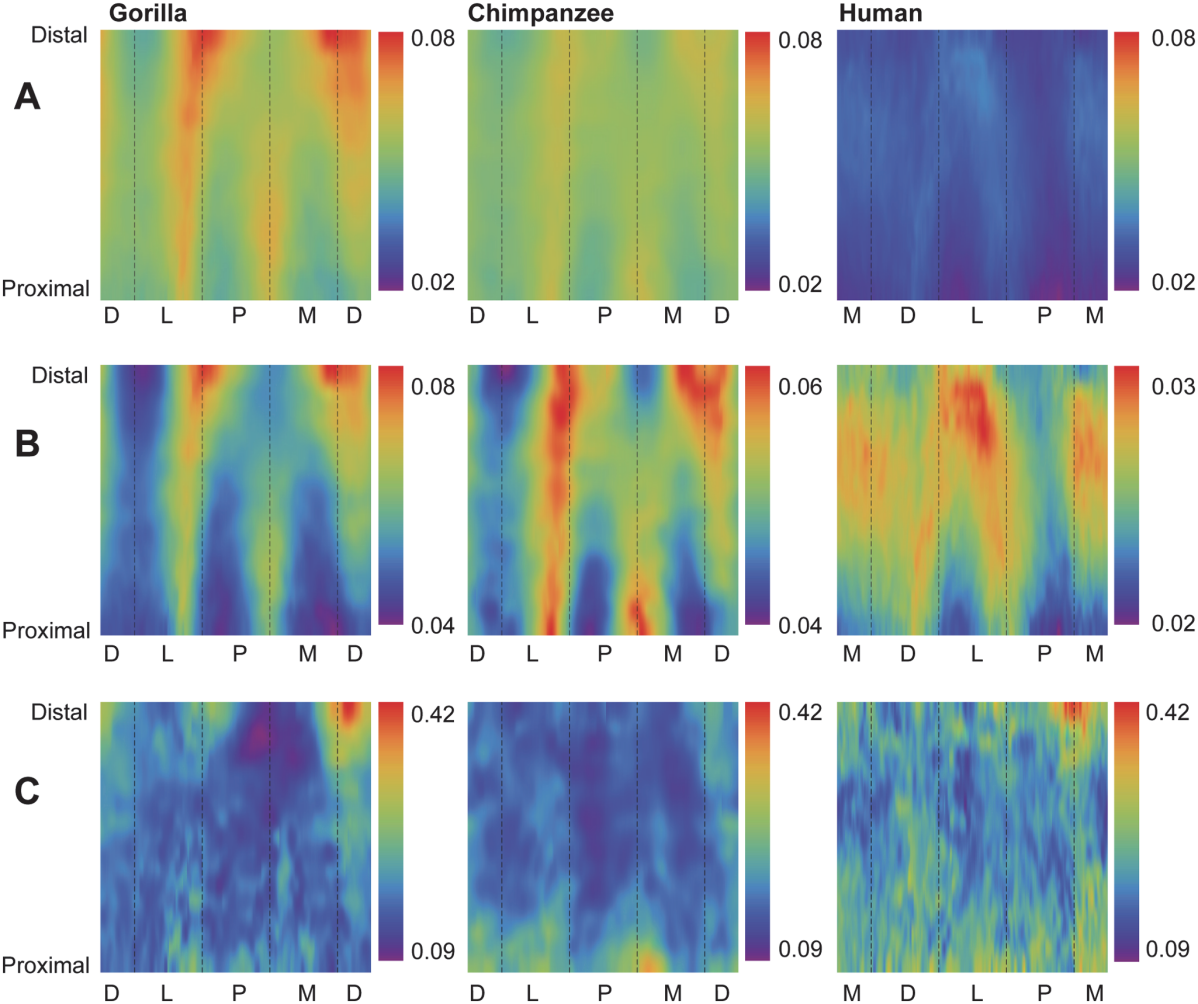
Comparisons of cortical bone thicknesses (CBTs). Due to differences in configurations resulting from hallucal abduction in chimpanzees and gorillas (see Fig. 1), functionally equivalent cortices (columns in the color map) differ in anatomical correspondence (i.e., dorsal cortices of chimpanzee and gorilla hallucal metatarsals are comparable with medial cortices of human hallucal metatarsals, etc.). **A**: Distribution of standardized cortical thickness visualized for interspecific comparisons. The color scheme is mapped to cortical thickness measurements standardized by length, thus creating dimensionless values. Amongst all pixels in the species consensus maps, global minimum (0.02) and maximum (0.08) values were used to establish the same range against which each species map was illustrated. Color maps demonstrate variation between species (e.g., gorillas exhibit the highest standardized cortical thickness, while humans exhibit the lowest). **B**: Distribution of standardized cortical thickness visualized for intraspecific comparisons. The color scheme is mapped to cortical thickness measurements standardized by length, thus creating dimensionless values. Amongst all pixels in respective species consensus maps, global minimum and maximum values were used to establish species-specific ranges for visualizing each map. **C**: Distribution of coefficients of variation (CVs) of standardized cortical thickness for interspecific comparisons. Minimum and maximum CVs from the three species were used to establish the same range with which each individual species color map was illustrated. Each CV color map visualizes the range of variation expressed within the diaphysis of a species. M—medial, D—dorsal, L—lateral, P—plantar.

In order to visualize group comparisons of standardized structural properties using color maps (*n* = 3 species), color bins were adjusted in two ways. First, in order to evaluate interspecific patterns amongst all pixels in the three species consensus maps, global minimum and maximum values were extracted and scaled to minimum “cold” (purple) and maximum “hot” (red) colors, respectively, such that each species map used the same possible range of colors (e.g., Fig. 3A). Second, in order to evaluate intraspecific patterns, minimum and maximum values were extracted from each individual consensus map of a species and scaled to the minimum “cold” color (purple) and the maximum “hot” color (red), respectively, for the individual species (e.g., Fig. 3B).

In order to quantitatively evaluate group patterns in diaphyseal regions expressing low and high variability of standardized structural properties (e.g., CBTs and SMAs), coefficients of variation (CVs) were calculated for each of the structural properties. Magnitudes of CVs were visualized using pixel colors, thus forming species-specific CV maps. As with other maps, each pixel corresponds to a unique combination of the specific radial position (e.g., measurement of cortical thickness or neutral plane) and diaphyseal cross section location (e.g., Fig. 3C). Global minimum and maximum CVs were extracted from across species maps and each species-specific CV map was rescaled to global minimum “cold” (purple) and maximum “hot” (red) colors, respectively. Intervening CVs were mapped to colors within the range defined by the observed extreme CVs.

### Penalized Discriminant Analysis

In order to further evaluate the hypotheses, we applied a PDA to morphometric data generated from the renderings [68]. Each PDA was implemented in Wolfram Mathematica^©^9 (Wolfram Research, Inc., Mathematica, Version 9.0.1, Champaign IL). Of the 43 metatarsal renderings, 36 were randomly assigned to a training sample (i.e., 12 per species), while the remaining 7 were assigned to a test sample (i.e., 2 chimpanzees, 2 gorillas, and 3 humans). A training sample of 36 individuals was chosen to optimize test sample size, and to permit flexibility in assigning cross-validation folds since choosing a multiple of 3 allowed equal representation of species (*n* = 3) in each fold. Penalized discriminant functions were fit to the training sample using 12-fold cross-validation in order to select regularization parameters [69] (S2 A–B Fig).

Correct classifications provide a measure of the predictive accuracy of discriminant functions after fitting. For CBTs, fit discriminant functions used 15.2 degrees of freedom; none of the training or test observations were misclassified. For SMAs, fit discriminant functions used 4.3 degrees of freedom; only one of the 36 training observations was misclassified (i.e., one chimpanzee was misclassified as a gorilla), while again, none of the seven test observations were misclassified.

## Results

### Group patterns in hallucal metatarsal mean structural properties (CBT)

Humans exhibit significantly lower absolute CBT (Table 2) and standardized CBT (Table 3) than chimpanzees and gorillas at 35%, 50%, and 65% diaphyseal cross sections. When systematically comparing group consensus standardized thicknesses across entire diaphyses, humans again stand out compared to chimpanzees and gorillas (Fig. 3A). While absolutely thinner in CBT, the human consensus pattern also exhibits local thicknesses in different locations of the metatarsal shaft (e.g., laterally, especially in the distal half of the diaphysis; medially in the midshaft) compared to consensus chimpanzee and gorilla patterns (e.g., dorsally, laterally and medially, especially in the distal half of the diaphysis) (Fig. 3B, S1 Fig).

**Table 2:** Non-scaled values for cortical bone thickness (CBT) and second moments of area (SMA).

	Homo sapiens	Gorilla gorilla	Pan troglodytes	Significant comparisons^#^
CBT (mm)	1.487	2.953	2.491	Gorilla > Homo
35% diaphysis*	-0.207	-0.378	-0.336	Gorilla > Pan
	0.70–2.80	1.74–5.41	1.52–3.64	Pan > Homo
CBT (mm)	1.676	3.164	2.585	Gorilla > Homo
50% diaphysis*	-0.191	-0.439	-0.326	Gorilla > Pan
	0.73–2.72	1.86–4.97	1.47–3.83	Pan > Homo
CBT (mm)	1.483	3.441	2.587	Gorilla > Homo
65% diaphysis*	-0.211	-0.684	-0.349	Gorilla > Pan
	0.56–2.74	1.55–7.84	1.46–4.20	Pan > Homo
SMA (mm^4^)	1538.4	1487.8	431.8	Homo > Pan
35% diaphysis*	-441.6	-782.9	-147.6	Gorilla > Pan
	680.2–2682.1	350.8–3172.3	253.0–909.4	
SMA (mm^4^)	1302.5	1288.9	363.8	Homo > Pan
50% diaphysis*	-411.7	-416.1	-116	Gorilla > Pan
	589.2–2490.7	290.9–2834.2	227.7–843.4	
SMA (mm^4^)	1305.3	1331.5	454.1	Gorilla > Pan
65% diaphysis*	-389.5	-755.9	-171.8	Homo > Pan
	511.2–2636.0	272.1–5417.7	243.3–1300.5	

A mean for each individual is calculated by averaging the 360 radial measurements for that individual at each indicated position of the diaphysis (i.e., 35%, 50%, and 65%). Summary statistics for individual subject means are reported here. Cells report mean in the top row, (1 s.d.) in the middle row, and range in the bottom row (global minimum and maximum values for a group).

*ANOVA, p < 0.001;

^#^Statistically significant Student-Newman-Keuls post hoc test results for the indicated pairings.

**Table 3:** Standardized values for cortical bone thickness (sCBT) and second moments of area (sSMA).

	Homo sapiens	Gorilla gorilla	Pan troglodytes	Significant comparisons^#^
sCBT	0.024	0.05	0.047	Gorilla > Homo
35% diaphysis*	-0.004	-0.006	-0.006	Pan > Homo
	0.011–0.046	0.027–0.096	0.029–0.070	
sCBT 50% diaphysis*	0.027	0.053	0.049	Gorilla > Homo
	-0.003	-0.007	-0.006	Gorilla > Pan
	0.012–0.044	0.029–0.090	0.028–0.072	Pan > Homo
sCBT	0.024	0.058	0.049	Gorilla > Homo
65% diaphysis*	-0.003	-0.01	-0.006	Gorilla > Pan
	0.009–0.045	0.025–0.132	0.028–0.077	Pan > Homo
sSMA	0.398	0.179	0.154	Homo > Gorilla
35% diaphysis*	-0.113	-0.05	-0.032	Homo > Pan
	0.207–0.729	0.072–0.277	0.102–0.280	
sSMA	0.337	0.127	0.13	Homo > Pan
50% diaphysis*	-0.103	-0.035	-0.025	Homo > Gorilla
	0.162–0.677	0.050–0.243	0.099–0.259	
sSMA	0.337	0.162	0.162	Homo > Gorilla
65% diaphysis*	-0.095	-0.05	-0.043	Homo > Pan
	0.141–0.717	0.047–0.465	0.104–0.400	

A mean for each individual is calculated by averaging the 360 radial measurements for that individual at each indicated position of the diaphysis (i.e., 35%, 50%, and 65%). Summary statistics for individual subject means are reported here. Cells report mean in the top row, (1 s.d.) in the middle row, and range in the bottom row (global minimum and maximum values for a group).

*ANOVA, p < 0.001;

^#^Statistically significant Student-Newman-Keuls post hoc test results for the indicated pairings.

**Fig 4:**
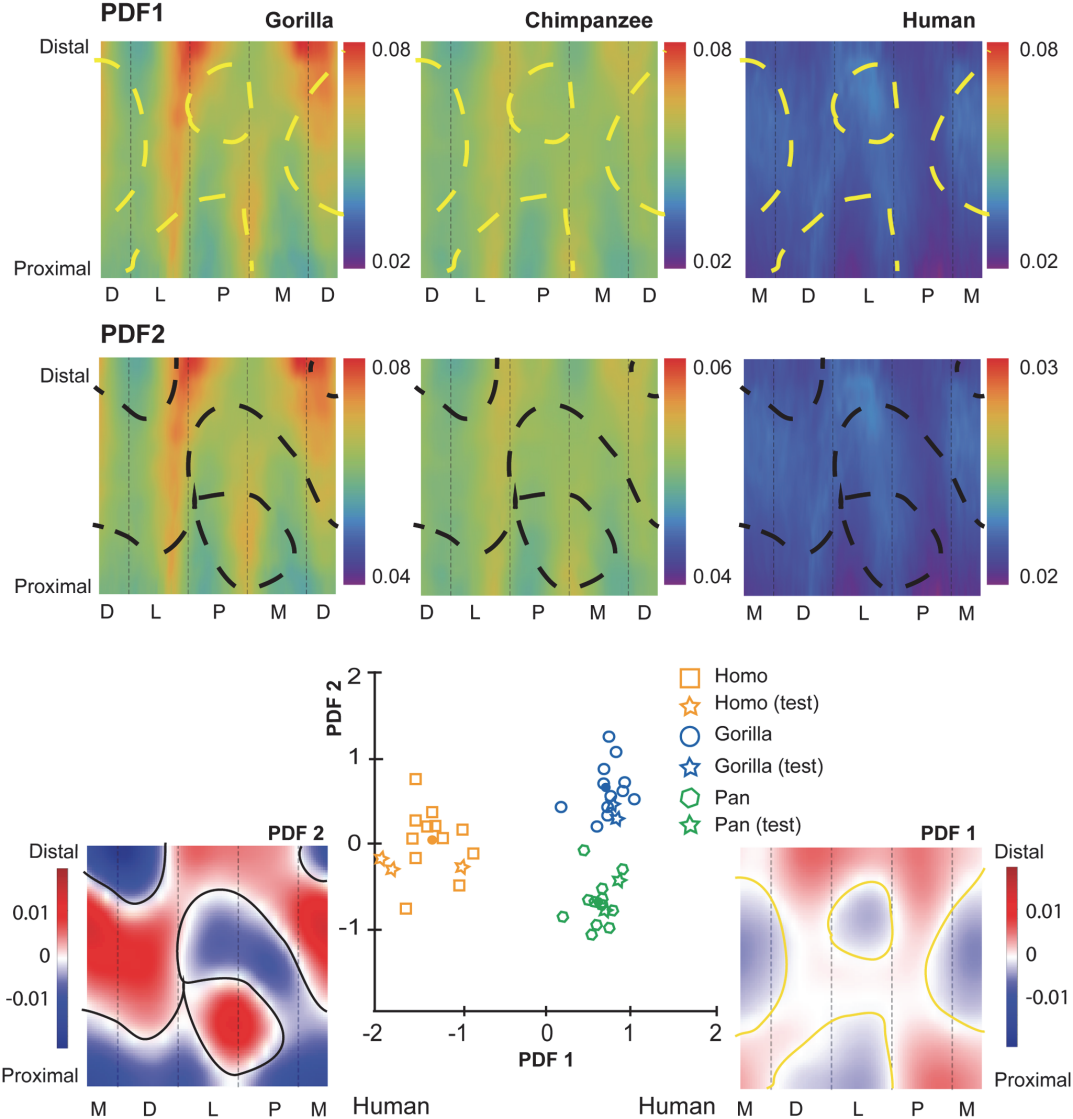
Penalized discriminant analysis (PDA) of standardized cortical bone thicknesses (CBTs). Due to differences in configurations resulting from hallucal abduction in chimpanzees and gorillas (see Fig. 1), functionally equivalent cortices (columns in the color map) differ in anatomical correspondence (i.e., dorsal cortices of chimpanzee and gorilla hallucal metatarsals are comparable with medial cortices of human hallucal metatarsals, etc.). Rows of color maps along the top (PDF1 and PDF2) visualize the distribution of mean scaled CBT for interspecific comparisons. In the uppermost row (PDF1), boundaries (dashed yellow lines) superimposed on consensus maps (see Fig. 3A) differentiate pixels with positive loading (red) from those with negative loading (blue) on PDF1. A positive loading for a given pixel indicates that a larger CBT value at that pixel increases the relative score on that discriminant axis. Similarly, a negative loading for a given pixel indicates that a larger CBT value at that pixel decreases the relative score on that discriminant axis. In the middle row (PDF2), boundaries (dashed black lines) superimposed on the same consensus maps differentiate pixels with positive loading (red) from those with negative loading (blue) for PDF2. Along the bottom, color maps (far left and far right in a red-blue colour scale) visualize pixel-wise loadings of 1^st^ and 2^nd^ penalized discriminant functions (PDF1 on the right and plotted on the horizontal axis of the centre scatter plot; PDF2 on the left and plotted on the vertical axis of the centre scatter plot). Note that white indicates the transition between positive and negative loadings (i.e., 0 loading by default). The bivariate scatter plot (bottom centre) presents the projection of each individual in the sample (*n* = 43; open symbols) into discriminant space via PDF1 and PDF2. Circles in the scatter plot indicate species means in discriminant space, effectively indicating group separation. Squares indicate subjects used in the training sample. Stars indicate test subjects. See the methods for an explanation of training versus test subjects. M—medial, D—dorsal, L—lateral, P—plantar.

The first penalized discriminant function separates standardized CBTs of humans from those of chimpanzees and gorillas (Fig. 4). When PDF1 is mapped to the diaphysis, it visualizes specific anatomical locations in which cortical thickness of human hallucal metatarsals can be distinguished from cortical thickness of chimpanzee and gorilla hallucal metatarsals (Fig. 4). Positive loadings are visualized as dark red regions, while negative loadings are visualized as dark blue regions. The mapped PDF1, in this manner, reinforces cortical thickness trends visualized in Fig. 3A-B.

Gorillas exhibit significantly higher absolute CBT (Table 2) than chimpanzees at 35%, 50%, and 65% diaphyseal locations, as well as significantly higher standardized CBT (Table 3) at 50%, and 65% diaphyseal locations. When systematically comparing group consensus standardized thicknesses across entire diaphyses, the consensus gorilla pattern exhibits higher thicknesses than the consensus chimpanzee pattern dorsally, laterally, and medially in cross sections, particularly in the distal region of the diaphysis (Fig. 3A). The consensus chimpanzee pattern, on the other hand, is relatively more uniform thickness along a cortical region of the hallucal metatarsal shaft compared to the consensus gorilla pattern (Fig. 3A-B), except for a distinct local thickness in the plantar and medial aspects of the proximal diaphysis that is absent from the consensus gorilla pattern (Fig. 3B).

The second penalized discriminant function separates standardized CBTs of chimpanzees and gorillas (Fig. 4). When PDF2 is mapped to the their diaphyses, it visualizes (as dark red or dark blue) dorsal and lateral areas of the midshaft region as being particularly useful for discriminating between chimpanzee (thinner) and gorilla (thicker) diaphyses (Fig. 4). It also visualizes plantar and medial regions of the their proximal diaphyses as useful for discriminating between chimpanzee (thinner) and gorilla (thicker) diaphyses (Fig. 4). The mapped PDF2, in this manner, reinforces cortical thickness trends that are visualized in Fig. 3A-B.

### Group patterns in hallucal metatarsal mean structural properties (SMA)

**Fig 5:**
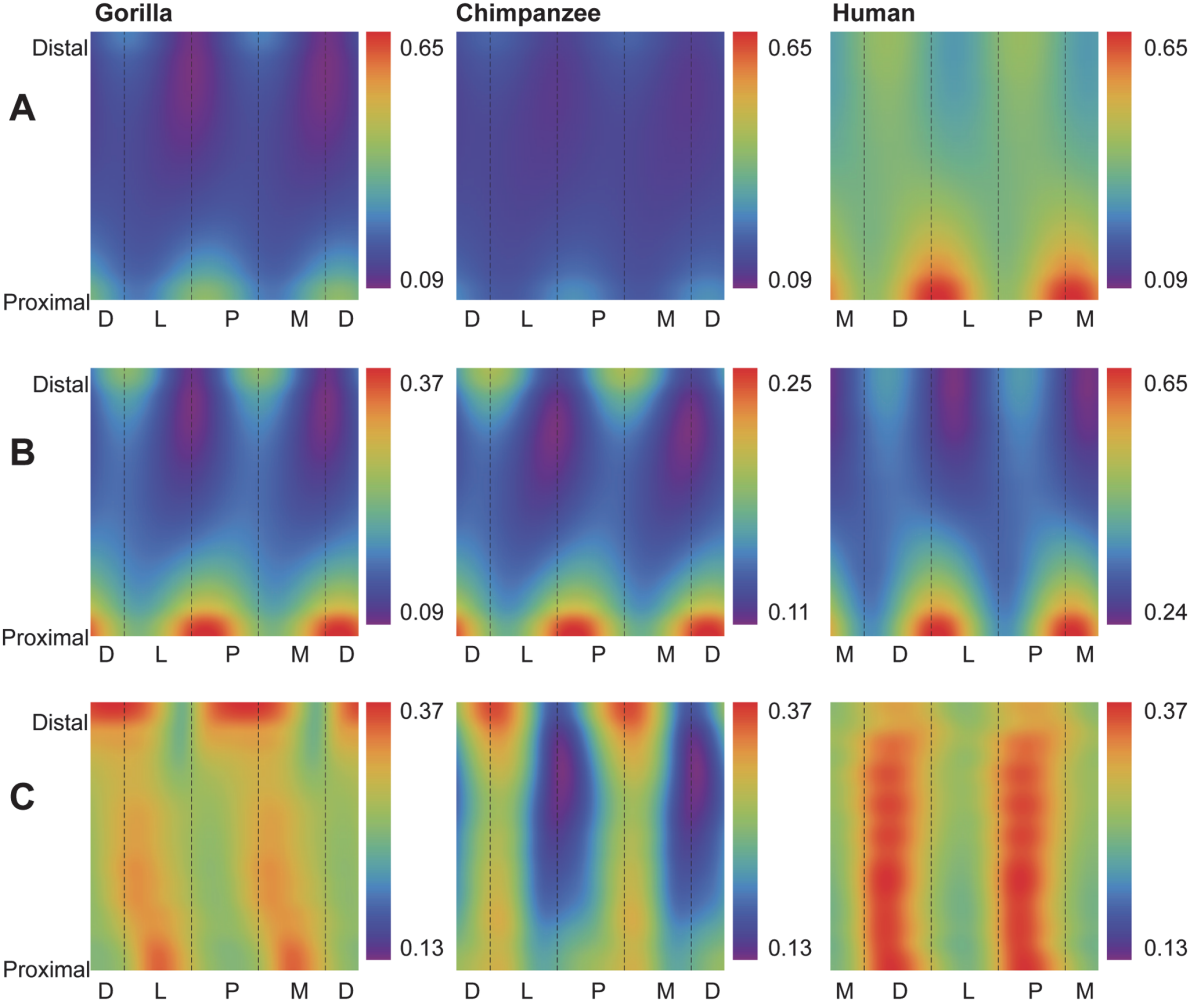
Comparisons of second moments of area (SMAs). Due to differences in configurations resulting from hallucal abduction in chimpanzees and gorillas (see Fig. 1), functionally equivalent cortices (columns in the color map) differ in anatomical correspondence (i.e., dorsal cortices of chimpanzee and gorilla hallucal metatarsals are comparable with medial cortices of human hallucal metatarsals, etc.). **A**: Distribution of standardized SMAs visualized for interspecific comparisons. The color scheme is mapped to SMAs standardized by the product of length and estimated body mass, creating mm^3^/kg values. Amongst all pixels in the species consensus maps, global minimum (0.87) and maximum (6.45) values were used to establish the same range against which each species map was illustrated. Color maps demonstrate variation between species (e.g., humans exhibit the highest standardized SMAs, while chimpanzees exhibit the lowest). **B**: Distribution of standardized SMAs visualized for intraspecific comparisons. The color scheme is mapped to SMAs standardized by the product of length and estimated body mass, creating mm^3^/kg values. Amongst all pixels in respective species consensus maps, global minimum and maximum values were used to establish species-specific ranges for visualizing each map. **C**: Distribution of coefficients of variation (CVs) of standardized SMAs for interspecific comparisons. Minimum and maximum CVs from the three species were used to establish the same range with which each individual species color map was illustrated. Each CV color map visualizes the range of variation expressed within the diaphysis of a species. M—medial, D—dorsal, L—lateral, P—plantar.

Humans exhibit significantly higher absolute SMAs (Table 2) than chimpanzees at 35%, 50%, and 65% diaphyseal locations, while differences from gorillas are non-significant. When comparing standardized SMAs (Table 3), however, humans are significantly higher than either chimpanzees or gorillas at 35%, 50%, and 65% diaphyseal locations. Humans are also distinguishable from chimpanzees and gorillas when systematically comparing group consensus standardized SMAs across diaphyses (Fig. 5A). While human, chimpanzee, and gorilla consensus maps exhibit functionally equivalent positions of maximal SMAs in the proximal hallucal metatarsal (i.e., lateral and plantar in chimpanzees/gorillas versus dorsal and lateral in humans), the human consensus map exhibits maximal SMAs dorsoplantarly in the distal half of the diaphysis compared to chimpanzee and gorilla consensus maps that exhibit maximal SMAs dorsolaterally and plantomedially (Fig. 5B). The human consensus map indicates a gradual internal rotation of maximal SMAs (i.e., orientation of maximum bending stiffness) proceeding proximodistally along the diaphysis. In contrast, consensus maps of chimpanzee and gorilla hallucal metatarsals indicate a gradual external rotation of maximal SMAs (i.e., orientation of maximum bending stiffness) proceeding proximodistally along the diaphysis (Fig. 5B, S3 Fig). The contrasting rotations are relative to the proximal end of diaphyses and reflect bone distribution within cross sections, and as such, they are not *a priori* reflections of different hallucal metatarsal head orientations.

**Fig 6:**
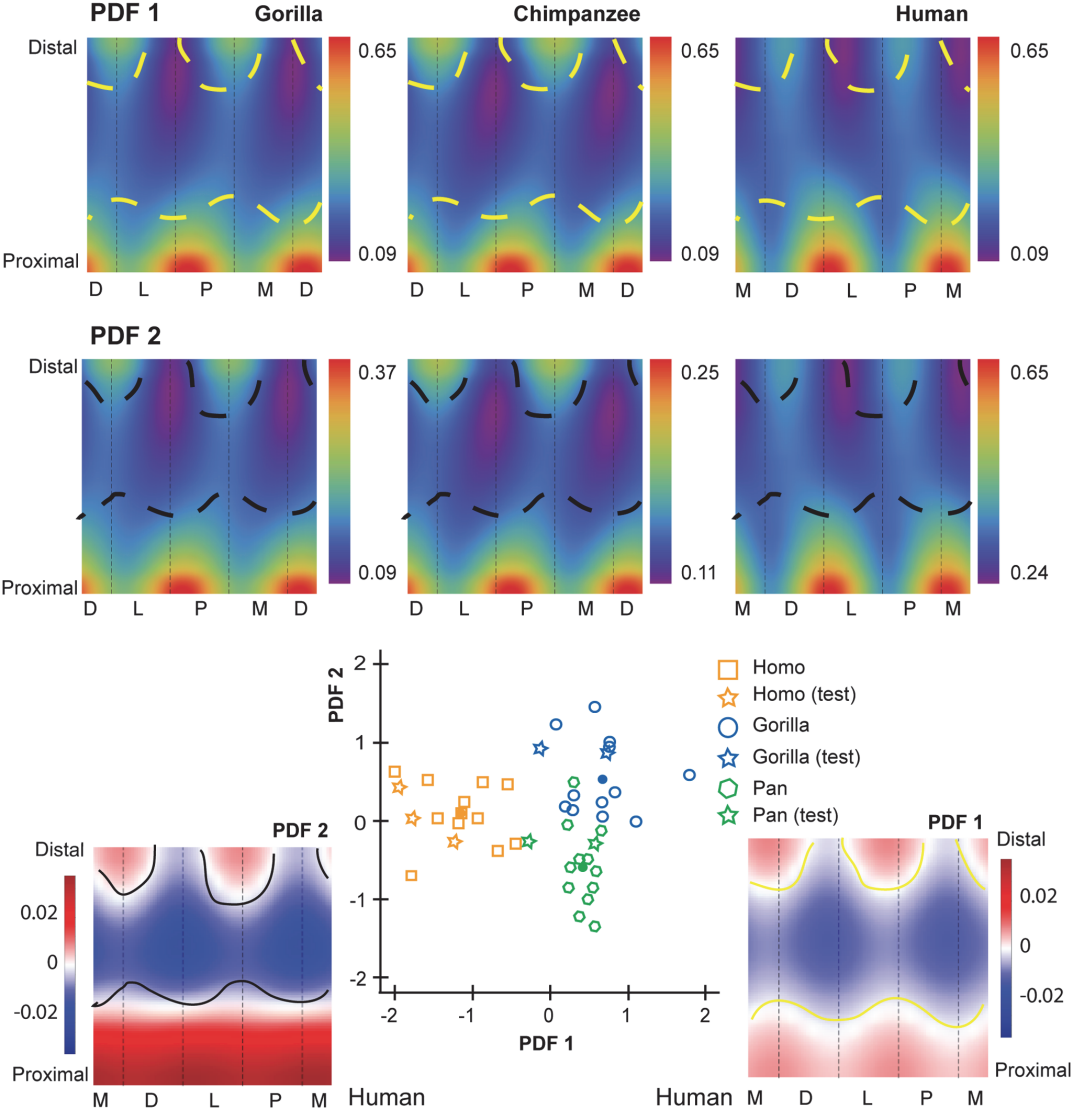
Penalized discriminant analysis (PDA) of standardized second moments of area (SMAs). Due to differences in configurations resulting from hallucal abduction in chimpanzees and gorillas (see Fig. 1), functionally equivalent cortices (columns in the color map) differ in anatomical correspondence (i.e., dorsal cortices of chimpanzee and gorilla hallucal metatarsals are comparable with medial cortices of human hallucal metatarsals, etc.). Rows of colour maps along the top (PDF1 and PDF2) visualize the distribution of mean scaled SMA for interspecific comparisons. In the uppermost row (PDF1), the boundaries (dashed yellow lines) superimposed on consensus maps (see Fig. 5A) differentiate pixels with positive loading (red) from those with negative loading (blue) on PDF1. A positive loading for a given pixel indicates that a larger SMA value at that pixel increases the relative score on that discriminant axis. Similarly, a negative loading for a given pixel indicates that a larger SMA value at that pixel decreases the relative score on that discriminant axis. In the middle row (PDF2), boundaries (dashed black lines) superimposed on the same consensus maps differentiate pixels with positive loading (red) from those with negative loading (blue) for PDF2. Along the bottom, color maps (far left and far right in a red-blue color scale) visualize pixel-wise loadings of 1^st^ and 2^nd^ penalized discriminant functions (PDF1 on the right and plotted on the horizontal axis of the centre scatter plot; PDF2 on the left and plotted on the vertical axis of centre scatter plot). Note that white indicates the transition between positive and negative loadings (i.e., 0 loading by default). The bivariate scatter plot (bottom centre) presents the projection of each individual in the sample (*n* = 43; open symbols) into discriminant space via PDF1 and PDF2. Circles in the scatter plot indicate species means in discriminant space, effectively indicating group separation. Squares indicate subjects used in the training sample. Stars indicate test subjects. See the methods for an explanation of training versus test subjects. M—medial, D—dorsal, L—lateral, P—plantar.

The first penalized discriminant function separates human standardized SMAs from those of chimpanzees and gorillas (Fig. 6). When PDF1 is mapped to the diaphysis, it visualizes contrasting orientations of maximal SMA orientation in human, chimpanzee, and gorilla hallucal metatarsals (Fig. 6). Red and blue regions in the distal diaphysis visualize the human hallucal metatarsal as having high SMAs dorsoplantarly (blue region), while chimpanzees and gorillas exhibit high SMAs along a dorsolateral to plantomedial axis (red region) (Fig. 6). The mapped PDF1, in this manner, reinforces trends in SMAs that are visible in Fig. 5A-B.

Gorillas exhibit significantly higher absolute SMAs (Table 2) than chimpanzees at 35%, 50%, and 65% diaphyseal locations. After standardizing SMAs, however, the difference between gorillas and chimpanzees essentially disappears (Table 3). When systematically comparing group consensus standardized SMAs across the entire diaphysis, the consensus gorilla pattern tends to exhibit slightly higher SMAs than the consensus chimpanzee pattern only in plantolateral and dorsomedial regions of the proximal shaft (Fig. 5A). When comparing other regions within the diaphysis, consensus gorilla and chimpanzee patterns exhibit very similar distributions (Fig. 5B).

The second penalized discriminant function tends to separate chimpanzee and gorilla standardized SMAs with the exception of one chimpanzee that is misclassified in the gorilla cluster (Fig. 6). When PDF2 is mapped to the diaphysis, it reinforces trends in SMAs that are visible in Fig. 5A-B, particularly the emphasis on a contrast between proximal and midshaft locations of hallucal metatarsals.

### Conserved versus variable regions in structural properties

Humans exhibit fewer radial CBT measurements around a given cross section with low CVs compared to chimpanzees and gorillas (i.e., humans exhibit fewer blue/purple pixels in color maps) (Fig. 3C), suggesting more variable CBT regions exist in humans and more conserved CBT regions exist in chimpanzees and gorillas. Local regions of human hallucal metatarsals with low CVs, however, can still be subtly distinguished in medial, lateral, and plantar aspects of the midshaft (Fig. 3C). Regions of chimpanzee hallucal metatarsals that exhibit low CVs are comparatively more apparent, particularly in the plantar and medial aspects of the distal diaphysis (Fig. 3C). Regions of gorilla hallucal metatarsals that exhibit low CVs are relatively concentrated in the proximal half of the diaphysis, except for a particularly prominent region in the plantomedial aspect of the distal diaphysis (Fig. 3C).

Chimpanzees exhibit more SMA measurements around a given cross section with low CVs compared to either humans or gorillas (i.e., chimpanzees exhibit more blue/purple pixels). This indicates more conserved SMA regions in chimpanzees and more variable SMA regions in humans and gorillas (Fig. 5C). Regions of chimpanzee hallucal metatarsals with low CVs are most apparent in plantolateral and dorsomedial aspects of the distal two-thirds of the diaphysis (Fig. 5C). Near midshaft, gorilla and human patterns diverge. Whereas regions of high variability in gorilla hallucal metatarsals occur laterally and medially in the proximal diaphysis, these regions rotate internally to occupy dorsolateral and plantomedial aspects of the diaphysis in distal regions of the diaphysis. Human hallucal metatarsals, on the other hand, retain dorsal and plantar locations of high variability for the entire length of the diaphysis (Fig. 5C).

## Discussion

Diaphyseal structure of human hallucal metatarsals is distinguishable from that of chimpanzee and gorilla hallucal metatarsals. While humans exhibit systematically thinner diaphyseal cortices than either of the other hominoids, even after having standardized cortical thickness by bone length, humans exhibit systematically greater standardized SMAs than other hominoids (Tables 2 and 3; Fig. 3A, 5A). As hypothesized, human hallucal metatarsals appear to be uniquely reinforced in the midshaft region (and more distally) to resist bending in a dorsoplantar orientation (Fig. 5A-B). Moreover, variability in standardized SMA magnitudes is uniquely prominent in dorsal and plantar cortices of human hallucal metatarsals (Fig. 5C), while medial and lateral cortices are comparatively more constrained in SMA magnitude variability. This is suggestive evidence for bone functional adaptations in the human hallucal metatarsal [55]. Specifically, human hallucal metatarsals vary less in orientations of high stiffness (e.g., suggesting relatively stereotyped dorsoplantar load orientations) than they vary in magnitudes of high stiffness (e.g., suggesting variable load sizes or frequencies). This finding would be consistent with humans in the sample exhibiting different amounts of relatively stereotypically-oriented loading (i.e., dorsoplantar bending), presumably reflecting different activity levels rather than different activity patterns or possibly the use of different substrates.

Evaluation of additional human groups could be illuminating in assessing the apparent form-function relationships observed in human hallucal metatarsals. The human foot has been shown to be plastically modified by the introduction and use of footwear [79]. Current best estimates for the antiquity of footwear do not extend beyond the Upper Palaeolithic (approximately 40,000 BP) based on suggested anatomical evidence [80–81], or much more recently (15,000 BP) based on direct evidence (e.g., actual footwear or depiction in rock art) [9]. Griffin and colleagues [51] assessed internal structure of hallucal metatarsals at midshaft in both habitually shod and unshod humans, observing no significant differences in standardized cross-sectional properties. As demonstrated in the present analysis, visualization and quantification of continuous cortical structure may elucidate more nuanced structural differences not readily discernible from single cross section comparisons (e.g., midshaft locations). Despite its unique configuration relative to other hominoids, the human foot does not preclude climbing from positively impacting fitness [82–84]. Analysing continuous diaphyseal structure in human subjects that utilize tree climbing, or arboreal activities more generally-speaking, also could be revealing as to how hominoid metatarsals reflect loading, since presumably human climbers should appear less like humans in the present sample. Finally, investigating continuous diaphyseal structure in hominin fossils (e.g., hallucal metatarsals) may pinpoint when the unique modern human configuration emerged in human evolutionary history, as well as provide further insight as to when important events such as obligate terrestrialism and footwear may have first occurred.

In general, variation in loading regimes experienced by hallucal metatarsals likely differs between humans versus chimpanzees and gorillas. Greater incorporation of arboreal substrate use during locomotor activities of the latter (e.g., climbing) likely introduces greater variation in loading regimes experienced by their hallucal metatarsals [85–86]. Moreover, orientation of the hallux in *Pan* varies considerably during stance, ranging from no contact with the substrate to moderate adduction [29]. It is likely that this amount of variability, presumably also expressed by gorillas, could be a contributing factor to the differentiation of their hallucal metatarsal diaphyses compared to humans. Nonetheless, consensus diaphyseal structure of chimpanzees and gorillas also can be differentiated according to standardized cortical thicknesses and SMAs (Table 3, Fig. 3–6), although these differences are more subtle than those uniquely distinguishing human hallucal metatarsals. The observed morphological differences between chimpanzee and gorilla hallucal metatarsals may be related, in part, to greater sexual dimorphism in gorilla body size, and consequently to greater dimorphism in gorilla arboreal locomotor behavior [87]. Present samples included both males and females from each taxon, which would not preclude these possibilities. However, full appreciation of the morphological differences between chimpanzee and gorilla hallucal metatarsal diaphyses is undermined by the absence of published gorilla plantar pressure data during terrestrial locomotion.

Support for the second hypothesis also was observed. Humans, chimpanzees, and gorillas exhibit regions of conserved low and high variability in hallucal metatarsals, though chimpanzee CV color maps exhibit less speckling than those of the other two groups. Rather, chimpanzees exhibit the most expansive, continuous areas of low variability in standardized cortical thickness, while humans exhibit the least expansive, continuous areas of low variability (Fig. 3C). Gorillas are approximately intermediate between the other two species (Fig. 3C). The observed higher overall CVs of human hallucal metatarsal thicknesses (Fig. 3C) may be an artefact of their low absolute mean thicknesses. When absolute mean thicknesses are higher, as in chimpanzees and gorillas, similar levels of variation will not be as apparent in CV maps simply because of the higher average mean thicknesses (denominators). By comparison, separation of the diaphysis into expansive and continuous areas of low versus high variability is comparatively more striking when quantifying standardized SMAs (Fig. 5C).

It is important to note that the extent of the structural differentiation documented in the present study is not replicated by comparing individual cross sections (e.g., see Tables 2–3). In analyzing metatarsal midshaft sections, Marchi [34] reported greater standardized CAs in chimpanzee hallucal metatarsals compared to those from either humans (intermediate) or gorillas (lowest). In the present study, which visualized and quantified cortical thicknesses in diaphyses of hallucal metatarsals from these same groups, gorillas exhibited similar or greater standardized cortical thicknesses than chimpanzees while both groups exceeded human values. The present study, similar to midshaft trends reported by Marchi [34], documented systematically greater stiffness throughout the diaphysis of human hallucal metatarsals compared to chimpanzees and gorillas. Unlike Marchi [34] who reported lower strength properties (i.e., standardized polar moments of area) in gorilla midshafts compared to chimpanzee midshafts, the present study documented relatively similar SMAs throughout the diaphysis of chimpanzees and gorillas. Despite this observed similarity, importantly, the present study was able to capture different patterns of SMA distributions in diaphyses of chimpanzee and gorilla hallucal metatarsals. Fully understanding the functional implications of the morphological differences and similarities in chimpanzees and gorillas in the present study, or the different results reported by Marchi [34] concerning gorilla hallucal metatarsal properties, is complicated by the aforementioned absence of plantar pressure data during gorilla terrestrial locomotion. It also cannot be ruled out that apparent contradictions between the present study and that of Marchi [34], particularly with regards to gorilla structural properties, may be partly attributable to sampling different individuals in the two studies, as well as the difference in data acquisition methods (e.g. [34] used biplanar radiographs to estimate cross-sectional properties).

Opposing rotations of maximal SMAs along the hallucal metatarsal diaphysis exhibited by gorillas and chimpanzees on the one hand (external), and humans on the other (internal), are intriguing, and previously unknown from metatarsal studies focusing on single diaphyseal locations (e.g., midshafts). While a functional explanation for these opposing trends is unclear, a difference in foot posture during terminal stance phase may be implicated. The human hallux is the last digit to break contact with the substrate during toe-off, while chimpanzees exhibit lateral roll-off during toe-off [29]. Position of the gorilla foot and pedal digits relative to the center of pressure during terminal stance is undocumented. The opposing human versus chimpanzee trend at toe-off appears to mirror the direction of opposing rotations of maximal SMAs along diaphyses in humans versus chimpanzees. Perhaps reorientation of maximal diaphyseal reinforcement within the distal diaphysis is partially reflecting structural constraints arising from the contrasting orientations of metatarsal heads on distal metaphyses.

The complexity of bone loading regimes during locomotor activity is well-established [52–55], but remains occasionally underappreciated when analyzing long bone structure (e.g., see [59]). Morimoto and colleagues [59] reported differences in ML bending strength of femora from captive and non-captive chimpanzee, which could be an indicator of different patterns of diaphyseal bending resulting from variation in pelvic breadth and femoral neck length, as has been suggested for different human groups [60]. Using a morphometric map approach, the present study has demonstrated that a range of high SMAs reinforce hallucal metatarsal diaphyses to either side of the direction of its greatest bending stiffness (Fig. 5). It is noteworthy that each of the three species exhibits similarly broad ranges of high SMAs in hallucal metatarsal cortices, despite experimental data suggesting differences exist in foot loading variability [29–30]. Demonstration of a range of strongly-reinforced directions bracketing the direction of maximum reinforcement is exciting since it suggests that even if bending orientations shift from the plane of maximum reinforcement (i.e., neutral axes passing through the centroid rotate several degrees), hallucal metatarsals are still well-reinforced to resist bending. This suggests a safety factor of sorts, or a range of high bending strengths bracketing the maximum bending strength, possibly in response to asymmetric bending, which may be more commonly experienced than appreciated in free-ranging animals [54]. Importantly, this information is unobtainable when analyzing selected cross sections and SMAs about specific neutral axes (e.g., AP and ML), but it could be a rich source of information for understanding the nature of bone functional adaptations given the relevance of mobility, and substrate complexity in particular [88].

We argue for the appropriateness of PDA when quantifying structural variation with a morphometric map approach. Other applications have focused on strictly visualization [60] or qualitative comparisons of structure [60]. The only other study that attempted to quantify structural differences [59] used a method of data exploration (PCA) rather than a technique for group discrimination (PDA). PDA mitigates the difficulty posed by having more variables than sample observations. One can accentuate regions that most ably discriminate between groups using a PDA. Cross-validation assesses whether fitted discriminant functions have predictive out-of-sample value. The PDA projection results in fewer discriminant functions to interpret; for example, in the present study there were only two since three groups were compared, all having centroids lying in a single plane. Additionally, PDA load projections for each discriminant axis can be visualized as a color map with positive and negative loadings, providing useful tools for interpreting the distributions along corresponding axes.

It is important to acknowledge limitations that exist in the present study, and more generally in the approach. While other studies using the morphometric map approach have not attempted to quantify variation within the sample, instead focusing on differentiating consensus patterns [59–62], CVs were informative, but ultimately proved to be a relatively crude instrument for visualizing and quantifying variability in standardized cortical thicknesses, as the human sample demonstrated (Fig. 3C). In the present study, the diaphysis was sampled only between 25% and 65% length in order to avoid regions where trabecular bone may have been present. Thus, structural information relevant for differentiating groups may have been inadvertently eliminated by restricting the area of investigation in this manner. The present study also did not consider diaphyseal curvature, nor have similar applications to the femoral diaphysis [59–62], which when present can impact loading regimes and region-specific cortical properties in curved bones such as phalanges [85], [89]. Computational needs for using this approach are not trivial, although with advancing technology these needs become less limiting. There is definitely a time and resource trade-off between using sample sizes in the hundreds, which would be more effective for quantifying variation within a taxon, versus sampling diaphyses with high resolution, which would be more effective for capturing subtle structural differences.

## Conclusions

The diaphysis of the hallucal metatarsal reflects loading history of the foot during stance phase of gait in hominoids. Here we quantified continuous thickness and second moments of area, discriminating between humans, chimpanzees, and gorillas with a PDA, and tested explicit hypotheses about loading histories and diaphyseal structure. Unlike chimpanzee and gorilla hallucal metatarsals, human hallucal metatarsals exhibit the greatest resistance to bending in dorsoplantar orientations. Chimpanzee and gorilla hallucal metatarsals exhibit greater obliquely-oriented resistance to bending, presumably due to the combined effects of terrestrial and arboreal locomotion on loading regimes. Importantly, hallucal metatarsals from each group exhibited a range of high rigidities bracketing the maximum stiffness. This may indicate that hallucal metatarsals are structured for resisting bending about several neutral axes rotating through the centroid, perhaps as a response to traversing complex substrates. The approach adopted here offers the potential for transformative insights when studying long bone diaphyseal structure, and may be particularly exciting for inferring new information on loading patterns of long bones in extinct organisms (e.g., fossils).

## Supporting Information

S1 Fig Distribution of standardized CBT for each individual in each of the three groups: gorilla, chimpanzee and human. The color scale for each map is constructed so as to range between the minimum and maximum standardized CBT values for that individual.
(TIF)

S2 Fig Expected PDA test error estimates and degrees of freedom for each variable. A. Cortical bone thickness (CBT). Expected test error estimates derived from 100 repeated stratified 12-fold cross-validation runs, with approximate 95% prediction errors (red band) and training error (green band). The degrees of freedom are selected by identifying all estimated test error rates below the minimum observed upper 95% prediction error bound and then choosing the lowest degrees of freedom amongst these (15.2 df for CBT). B. Second moment of area (SMA). Expected test error estimates derived from 100 repeated stratified 12-fold cross-validation runs, with approximate 95% prediction errors (red band) and training error (green band). The degrees of freedom are selected by identifying all estimated test error rates below the minimum observed upper 95% prediction error bound and then choosing the lowest degrees of freedom amongst these (4.3 df for SMA).
(TIF)

S3 Fig Distribution of standardized SMA for each individual in each of the three groups: gorilla, chimpanzee and human. The color scale for each map is constructed so as to range between the minimum and maximum standardized SMA values for that individual.
(TIF)

S1 File X-ray images of hallucal metatarsals of 43 extant, adult hominoids (S1 Text). Individuals in central chimpanzee (Pan troglodytes troglodytes) and western lowland gorilla (Gorilla gorilla gorilla) are housed in the Primate Collection of the Department of Comparative Anatomy of the National Museum of Natural History, Paris, France (S1 Text). Humans (Homo sapiens) are housed in the Raymond A. Dart Collection of Human Skeletons at the University of the Witwatersrand, Johannesburg, South Africa (S1 Text) [70].
(ZIP)

S1 Text Sample and collections. (DOC)

S2 Text Methodology for extracting contours of cross-sections via kernel smoothing). (DOC)
