# Supplementary figures and images for: Cortical Structure of Hallucal Metatarsals and Locomotor Adaptations in Hominoids (part 1 of 3)
Source: PLoS One. 2015 Jan 30;10(1):e0117905. doi: 10.1371/journal.pone.0117905 (PMC4311976; doi:10.1371/journal.pone.0117905)

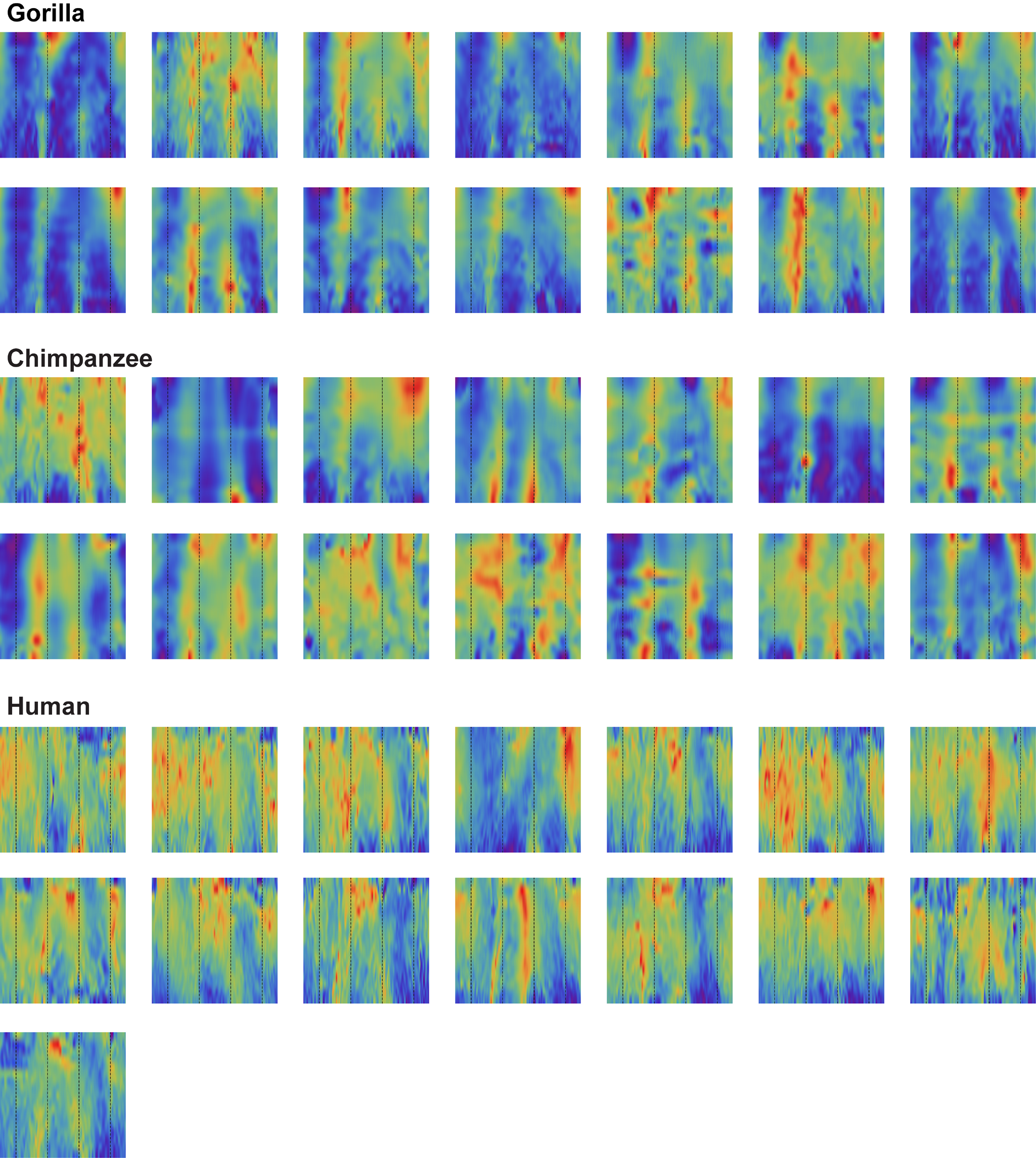

Supplement: S1 Fig — The color scale for each map is constructed so as to range between the minimum and maximum standardized CBT values for that individual. (TIF) [file pone.0117905.s001.tif]

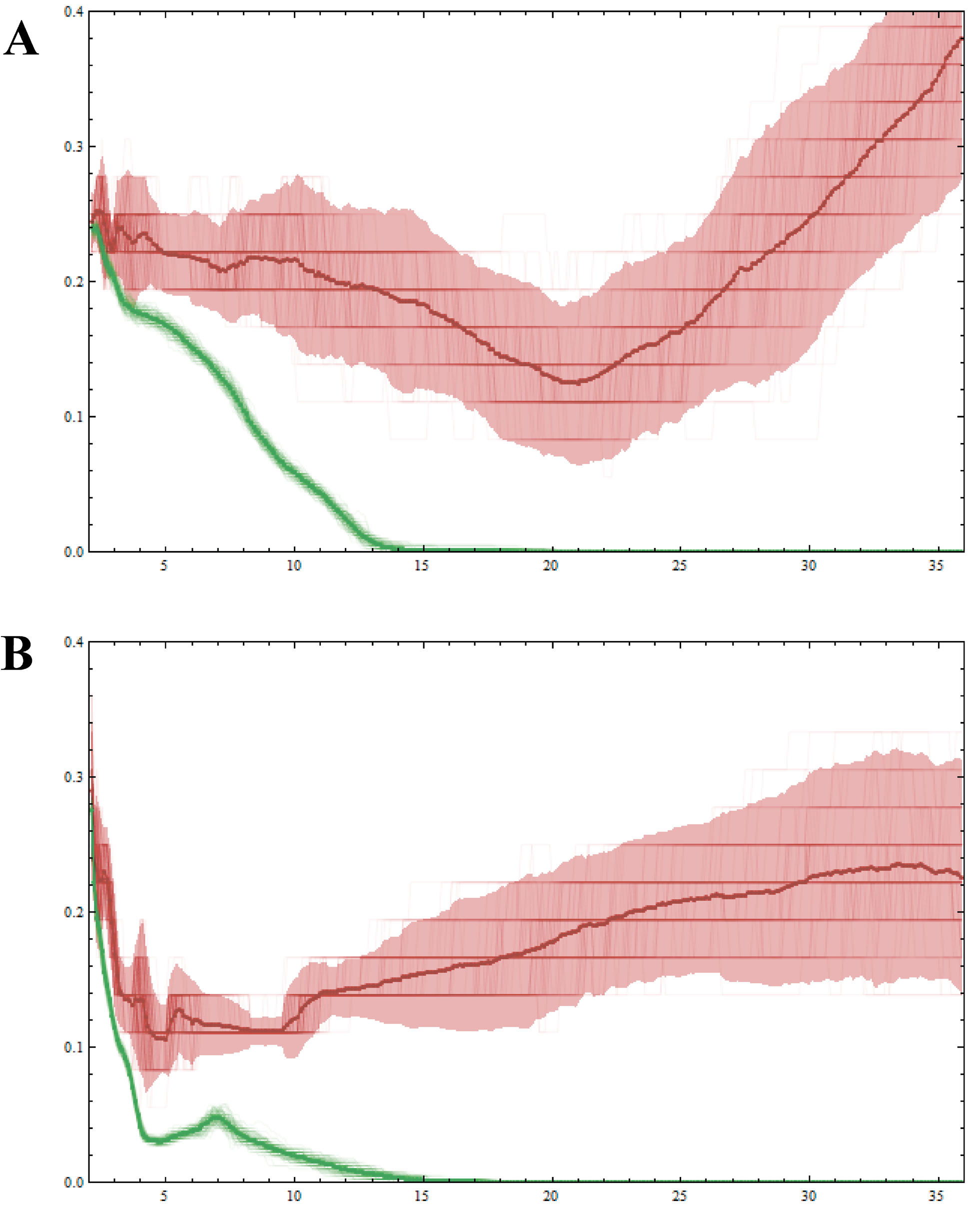

Supplement: S2 Fig — A. Cortical bone thickness (CBT). Expected test error estimates derived from 100 repeated stratified 12-fold cross-validation runs, with approximate 95% prediction errors (red band) and training error (green band). The degrees of freedom are selected by identifying all estimated test error rates below the minimum observed upper 95% prediction error bound and then choosing the lowest degrees of freedom amongst these (15.2 df for CBT). B. Second moment of area (SMA). Expected test error estimates derived from 100 repeated stratified 12-fold cross-validation runs, with approximate 95% prediction errors (red band) and training error (green band). The degrees of freedom are selected by identifying all estimated test error rates below the minimum observed upper 95% prediction error bound and then choosing the lowest degrees of freedom amongst these (4.3 df for SMA). (TIF) [file pone.0117905.s002.tif]

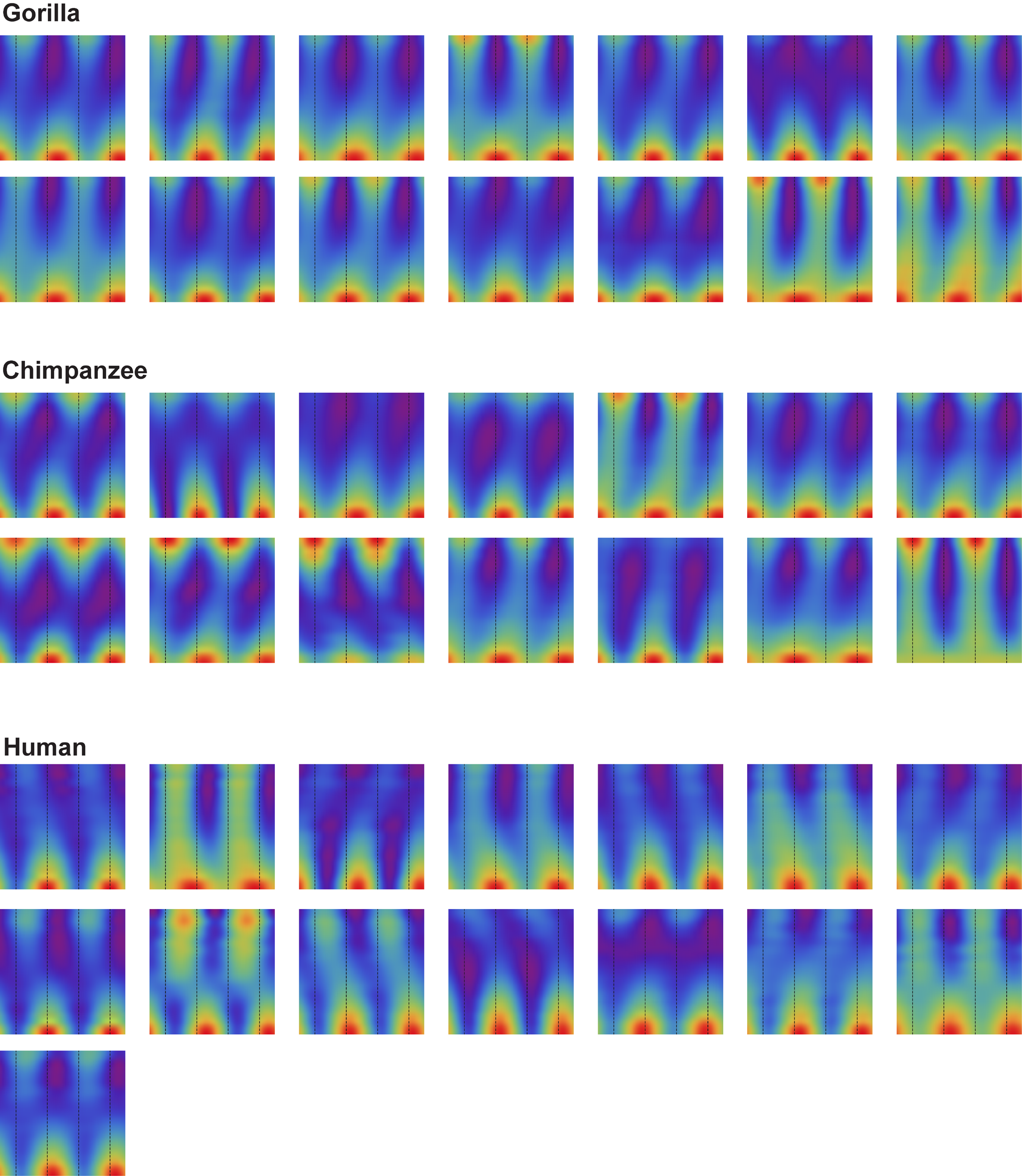

Supplement: S3 Fig — The color scale for each map is constructed so as to range between the minimum and maximum standardized SMA values for that individual. (TIF) [file pone.0117905.s003.tif]

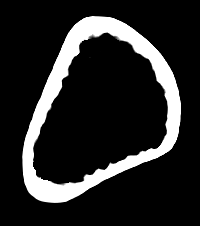

Supplement: S1 File — Individuals in central chimpanzee (Pan troglodytes troglodytes) and western lowland gorilla (Gorilla gorilla gorilla) are housed in the Primate Collection of the Department of Comparative Anatomy of the National Museum of Natural History, Paris, France (S1 Text). Humans (Homo sapiens) are housed in the Raymond A. Dart Collection of Human Skeletons at the University of the Witwatersrand, Johannesburg, South Africa (S1 Text) [70]. (ZIP) [file pone.0117905.s004.zip › X-Ray_Cortical structure of hallucal metatarsals and locomotor adaptations in hominoids/20_M_A9_Vend_MT1L.0000.bmp]

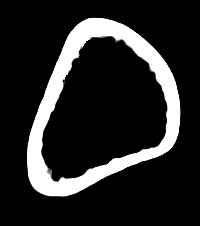

Supplement: S1 File — Individuals in central chimpanzee (Pan troglodytes troglodytes) and western lowland gorilla (Gorilla gorilla gorilla) are housed in the Primate Collection of the Department of Comparative Anatomy of the National Museum of Natural History, Paris, France (S1 Text). Humans (Homo sapiens) are housed in the Raymond A. Dart Collection of Human Skeletons at the University of the Witwatersrand, Johannesburg, South Africa (S1 Text) [70]. (ZIP) [file pone.0117905.s004.zip › X-Ray_Cortical structure of hallucal metatarsals and locomotor adaptations in hominoids/20_M_A9_Vend_MT1L.0001.bmp]

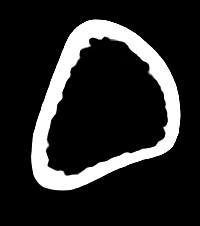

Supplement: S1 File — Individuals in central chimpanzee (Pan troglodytes troglodytes) and western lowland gorilla (Gorilla gorilla gorilla) are housed in the Primate Collection of the Department of Comparative Anatomy of the National Museum of Natural History, Paris, France (S1 Text). Humans (Homo sapiens) are housed in the Raymond A. Dart Collection of Human Skeletons at the University of the Witwatersrand, Johannesburg, South Africa (S1 Text) [70]. (ZIP) [file pone.0117905.s004.zip › X-Ray_Cortical structure of hallucal metatarsals and locomotor adaptations in hominoids/20_M_A9_Vend_MT1L.0002.bmp]

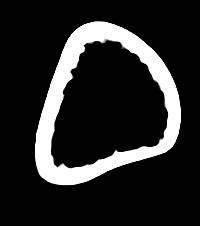

Supplement: S1 File — Individuals in central chimpanzee (Pan troglodytes troglodytes) and western lowland gorilla (Gorilla gorilla gorilla) are housed in the Primate Collection of the Department of Comparative Anatomy of the National Museum of Natural History, Paris, France (S1 Text). Humans (Homo sapiens) are housed in the Raymond A. Dart Collection of Human Skeletons at the University of the Witwatersrand, Johannesburg, South Africa (S1 Text) [70]. (ZIP) [file pone.0117905.s004.zip › X-Ray_Cortical structure of hallucal metatarsals and locomotor adaptations in hominoids/20_M_A9_Vend_MT1L.0003.bmp]

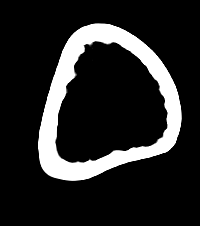

Supplement: S1 File — Individuals in central chimpanzee (Pan troglodytes troglodytes) and western lowland gorilla (Gorilla gorilla gorilla) are housed in the Primate Collection of the Department of Comparative Anatomy of the National Museum of Natural History, Paris, France (S1 Text). Humans (Homo sapiens) are housed in the Raymond A. Dart Collection of Human Skeletons at the University of the Witwatersrand, Johannesburg, South Africa (S1 Text) [70]. (ZIP) [file pone.0117905.s004.zip › X-Ray_Cortical structure of hallucal metatarsals and locomotor adaptations in hominoids/20_M_A9_Vend_MT1L.0004.bmp]

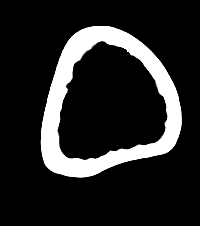

Supplement: S1 File — Individuals in central chimpanzee (Pan troglodytes troglodytes) and western lowland gorilla (Gorilla gorilla gorilla) are housed in the Primate Collection of the Department of Comparative Anatomy of the National Museum of Natural History, Paris, France (S1 Text). Humans (Homo sapiens) are housed in the Raymond A. Dart Collection of Human Skeletons at the University of the Witwatersrand, Johannesburg, South Africa (S1 Text) [70]. (ZIP) [file pone.0117905.s004.zip › X-Ray_Cortical structure of hallucal metatarsals and locomotor adaptations in hominoids/20_M_A9_Vend_MT1L.0005.bmp]

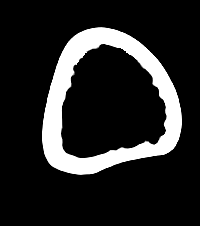

Supplement: S1 File — Individuals in central chimpanzee (Pan troglodytes troglodytes) and western lowland gorilla (Gorilla gorilla gorilla) are housed in the Primate Collection of the Department of Comparative Anatomy of the National Museum of Natural History, Paris, France (S1 Text). Humans (Homo sapiens) are housed in the Raymond A. Dart Collection of Human Skeletons at the University of the Witwatersrand, Johannesburg, South Africa (S1 Text) [70]. (ZIP) [file pone.0117905.s004.zip › X-Ray_Cortical structure of hallucal metatarsals and locomotor adaptations in hominoids/20_M_A9_Vend_MT1L.0006.bmp]

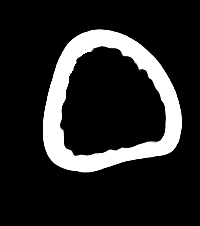

Supplement: S1 File — Individuals in central chimpanzee (Pan troglodytes troglodytes) and western lowland gorilla (Gorilla gorilla gorilla) are housed in the Primate Collection of the Department of Comparative Anatomy of the National Museum of Natural History, Paris, France (S1 Text). Humans (Homo sapiens) are housed in the Raymond A. Dart Collection of Human Skeletons at the University of the Witwatersrand, Johannesburg, South Africa (S1 Text) [70]. (ZIP) [file pone.0117905.s004.zip › X-Ray_Cortical structure of hallucal metatarsals and locomotor adaptations in hominoids/20_M_A9_Vend_MT1L.0007.bmp]

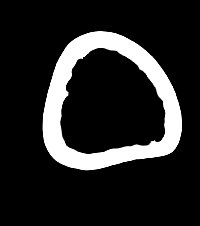

Supplement: S1 File — Individuals in central chimpanzee (Pan troglodytes troglodytes) and western lowland gorilla (Gorilla gorilla gorilla) are housed in the Primate Collection of the Department of Comparative Anatomy of the National Museum of Natural History, Paris, France (S1 Text). Humans (Homo sapiens) are housed in the Raymond A. Dart Collection of Human Skeletons at the University of the Witwatersrand, Johannesburg, South Africa (S1 Text) [70]. (ZIP) [file pone.0117905.s004.zip › X-Ray_Cortical structure of hallucal metatarsals and locomotor adaptations in hominoids/20_M_A9_Vend_MT1L.0008.bmp]

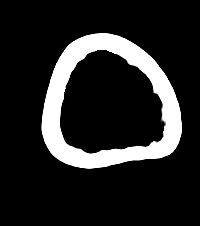

Supplement: S1 File — Individuals in central chimpanzee (Pan troglodytes troglodytes) and western lowland gorilla (Gorilla gorilla gorilla) are housed in the Primate Collection of the Department of Comparative Anatomy of the National Museum of Natural History, Paris, France (S1 Text). Humans (Homo sapiens) are housed in the Raymond A. Dart Collection of Human Skeletons at the University of the Witwatersrand, Johannesburg, South Africa (S1 Text) [70]. (ZIP) [file pone.0117905.s004.zip › X-Ray_Cortical structure of hallucal metatarsals and locomotor adaptations in hominoids/20_M_A9_Vend_MT1L.0009.bmp]

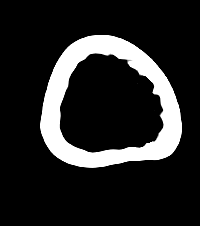

Supplement: S1 File — Individuals in central chimpanzee (Pan troglodytes troglodytes) and western lowland gorilla (Gorilla gorilla gorilla) are housed in the Primate Collection of the Department of Comparative Anatomy of the National Museum of Natural History, Paris, France (S1 Text). Humans (Homo sapiens) are housed in the Raymond A. Dart Collection of Human Skeletons at the University of the Witwatersrand, Johannesburg, South Africa (S1 Text) [70]. (ZIP) [file pone.0117905.s004.zip › X-Ray_Cortical structure of hallucal metatarsals and locomotor adaptations in hominoids/20_M_A9_Vend_MT1L.0010.bmp]

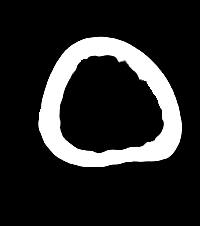

Supplement: S1 File — Individuals in central chimpanzee (Pan troglodytes troglodytes) and western lowland gorilla (Gorilla gorilla gorilla) are housed in the Primate Collection of the Department of Comparative Anatomy of the National Museum of Natural History, Paris, France (S1 Text). Humans (Homo sapiens) are housed in the Raymond A. Dart Collection of Human Skeletons at the University of the Witwatersrand, Johannesburg, South Africa (S1 Text) [70]. (ZIP) [file pone.0117905.s004.zip › X-Ray_Cortical structure of hallucal metatarsals and locomotor adaptations in hominoids/20_M_A9_Vend_MT1L.0011.bmp]

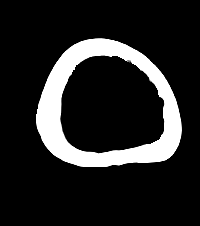

Supplement: S1 File — Individuals in central chimpanzee (Pan troglodytes troglodytes) and western lowland gorilla (Gorilla gorilla gorilla) are housed in the Primate Collection of the Department of Comparative Anatomy of the National Museum of Natural History, Paris, France (S1 Text). Humans (Homo sapiens) are housed in the Raymond A. Dart Collection of Human Skeletons at the University of the Witwatersrand, Johannesburg, South Africa (S1 Text) [70]. (ZIP) [file pone.0117905.s004.zip › X-Ray_Cortical structure of hallucal metatarsals and locomotor adaptations in hominoids/20_M_A9_Vend_MT1L.0012.bmp]

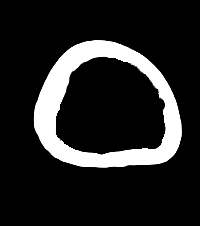

Supplement: S1 File — Individuals in central chimpanzee (Pan troglodytes troglodytes) and western lowland gorilla (Gorilla gorilla gorilla) are housed in the Primate Collection of the Department of Comparative Anatomy of the National Museum of Natural History, Paris, France (S1 Text). Humans (Homo sapiens) are housed in the Raymond A. Dart Collection of Human Skeletons at the University of the Witwatersrand, Johannesburg, South Africa (S1 Text) [70]. (ZIP) [file pone.0117905.s004.zip › X-Ray_Cortical structure of hallucal metatarsals and locomotor adaptations in hominoids/20_M_A9_Vend_MT1L.0013.bmp]

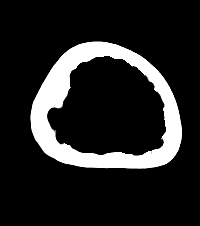

Supplement: S1 File — Individuals in central chimpanzee (Pan troglodytes troglodytes) and western lowland gorilla (Gorilla gorilla gorilla) are housed in the Primate Collection of the Department of Comparative Anatomy of the National Museum of Natural History, Paris, France (S1 Text). Humans (Homo sapiens) are housed in the Raymond A. Dart Collection of Human Skeletons at the University of the Witwatersrand, Johannesburg, South Africa (S1 Text) [70]. (ZIP) [file pone.0117905.s004.zip › X-Ray_Cortical structure of hallucal metatarsals and locomotor adaptations in hominoids/20_M_A9_Vend_MT1L.0014.bmp]

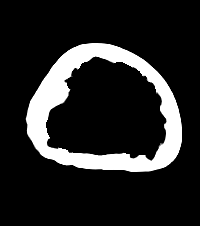

Supplement: S1 File — Individuals in central chimpanzee (Pan troglodytes troglodytes) and western lowland gorilla (Gorilla gorilla gorilla) are housed in the Primate Collection of the Department of Comparative Anatomy of the National Museum of Natural History, Paris, France (S1 Text). Humans (Homo sapiens) are housed in the Raymond A. Dart Collection of Human Skeletons at the University of the Witwatersrand, Johannesburg, South Africa (S1 Text) [70]. (ZIP) [file pone.0117905.s004.zip › X-Ray_Cortical structure of hallucal metatarsals and locomotor adaptations in hominoids/20_M_A9_Vend_MT1L.0015.bmp]

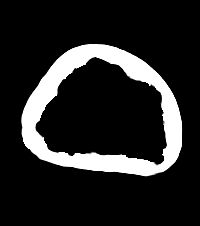

Supplement: S1 File — Individuals in central chimpanzee (Pan troglodytes troglodytes) and western lowland gorilla (Gorilla gorilla gorilla) are housed in the Primate Collection of the Department of Comparative Anatomy of the National Museum of Natural History, Paris, France (S1 Text). Humans (Homo sapiens) are housed in the Raymond A. Dart Collection of Human Skeletons at the University of the Witwatersrand, Johannesburg, South Africa (S1 Text) [70]. (ZIP) [file pone.0117905.s004.zip › X-Ray_Cortical structure of hallucal metatarsals and locomotor adaptations in hominoids/20_M_A9_Vend_MT1L.0016.bmp]

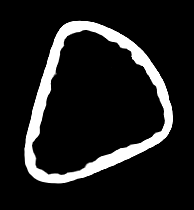

Supplement: S1 File — Individuals in central chimpanzee (Pan troglodytes troglodytes) and western lowland gorilla (Gorilla gorilla gorilla) are housed in the Primate Collection of the Department of Comparative Anatomy of the National Museum of Natural History, Paris, France (S1 Text). Humans (Homo sapiens) are housed in the Raymond A. Dart Collection of Human Skeletons at the University of the Witwatersrand, Johannesburg, South Africa (S1 Text) [70]. (ZIP) [file pone.0117905.s004.zip › X-Ray_Cortical structure of hallucal metatarsals and locomotor adaptations in hominoids/24_F_A458_Soto_MT1L.0000.bmp]

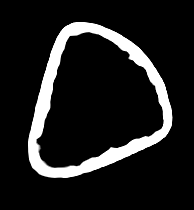

Supplement: S1 File — Individuals in central chimpanzee (Pan troglodytes troglodytes) and western lowland gorilla (Gorilla gorilla gorilla) are housed in the Primate Collection of the Department of Comparative Anatomy of the National Museum of Natural History, Paris, France (S1 Text). Humans (Homo sapiens) are housed in the Raymond A. Dart Collection of Human Skeletons at the University of the Witwatersrand, Johannesburg, South Africa (S1 Text) [70]. (ZIP) [file pone.0117905.s004.zip › X-Ray_Cortical structure of hallucal metatarsals and locomotor adaptations in hominoids/24_F_A458_Soto_MT1L.0001.bmp]

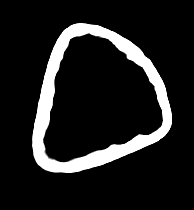

Supplement: S1 File — Individuals in central chimpanzee (Pan troglodytes troglodytes) and western lowland gorilla (Gorilla gorilla gorilla) are housed in the Primate Collection of the Department of Comparative Anatomy of the National Museum of Natural History, Paris, France (S1 Text). Humans (Homo sapiens) are housed in the Raymond A. Dart Collection of Human Skeletons at the University of the Witwatersrand, Johannesburg, South Africa (S1 Text) [70]. (ZIP) [file pone.0117905.s004.zip › X-Ray_Cortical structure of hallucal metatarsals and locomotor adaptations in hominoids/24_F_A458_Soto_MT1L.0002.bmp]

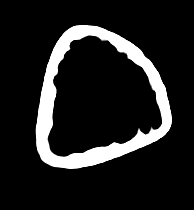

Supplement: S1 File — Individuals in central chimpanzee (Pan troglodytes troglodytes) and western lowland gorilla (Gorilla gorilla gorilla) are housed in the Primate Collection of the Department of Comparative Anatomy of the National Museum of Natural History, Paris, France (S1 Text). Humans (Homo sapiens) are housed in the Raymond A. Dart Collection of Human Skeletons at the University of the Witwatersrand, Johannesburg, South Africa (S1 Text) [70]. (ZIP) [file pone.0117905.s004.zip › X-Ray_Cortical structure of hallucal metatarsals and locomotor adaptations in hominoids/24_F_A458_Soto_MT1L.0003.bmp]

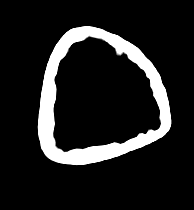

Supplement: S1 File — Individuals in central chimpanzee (Pan troglodytes troglodytes) and western lowland gorilla (Gorilla gorilla gorilla) are housed in the Primate Collection of the Department of Comparative Anatomy of the National Museum of Natural History, Paris, France (S1 Text). Humans (Homo sapiens) are housed in the Raymond A. Dart Collection of Human Skeletons at the University of the Witwatersrand, Johannesburg, South Africa (S1 Text) [70]. (ZIP) [file pone.0117905.s004.zip › X-Ray_Cortical structure of hallucal metatarsals and locomotor adaptations in hominoids/24_F_A458_Soto_MT1L.0004.bmp]

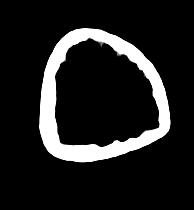

Supplement: S1 File — Individuals in central chimpanzee (Pan troglodytes troglodytes) and western lowland gorilla (Gorilla gorilla gorilla) are housed in the Primate Collection of the Department of Comparative Anatomy of the National Museum of Natural History, Paris, France (S1 Text). Humans (Homo sapiens) are housed in the Raymond A. Dart Collection of Human Skeletons at the University of the Witwatersrand, Johannesburg, South Africa (S1 Text) [70]. (ZIP) [file pone.0117905.s004.zip › X-Ray_Cortical structure of hallucal metatarsals and locomotor adaptations in hominoids/24_F_A458_Soto_MT1L.0005.bmp]

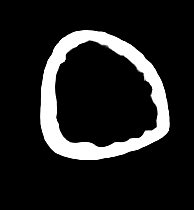

Supplement: S1 File — Individuals in central chimpanzee (Pan troglodytes troglodytes) and western lowland gorilla (Gorilla gorilla gorilla) are housed in the Primate Collection of the Department of Comparative Anatomy of the National Museum of Natural History, Paris, France (S1 Text). Humans (Homo sapiens) are housed in the Raymond A. Dart Collection of Human Skeletons at the University of the Witwatersrand, Johannesburg, South Africa (S1 Text) [70]. (ZIP) [file pone.0117905.s004.zip › X-Ray_Cortical structure of hallucal metatarsals and locomotor adaptations in hominoids/24_F_A458_Soto_MT1L.0006.bmp]

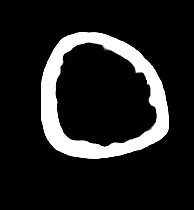

Supplement: S1 File — Individuals in central chimpanzee (Pan troglodytes troglodytes) and western lowland gorilla (Gorilla gorilla gorilla) are housed in the Primate Collection of the Department of Comparative Anatomy of the National Museum of Natural History, Paris, France (S1 Text). Humans (Homo sapiens) are housed in the Raymond A. Dart Collection of Human Skeletons at the University of the Witwatersrand, Johannesburg, South Africa (S1 Text) [70]. (ZIP) [file pone.0117905.s004.zip › X-Ray_Cortical structure of hallucal metatarsals and locomotor adaptations in hominoids/24_F_A458_Soto_MT1L.0007.bmp]

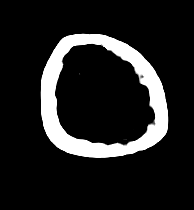

Supplement: S1 File — Individuals in central chimpanzee (Pan troglodytes troglodytes) and western lowland gorilla (Gorilla gorilla gorilla) are housed in the Primate Collection of the Department of Comparative Anatomy of the National Museum of Natural History, Paris, France (S1 Text). Humans (Homo sapiens) are housed in the Raymond A. Dart Collection of Human Skeletons at the University of the Witwatersrand, Johannesburg, South Africa (S1 Text) [70]. (ZIP) [file pone.0117905.s004.zip › X-Ray_Cortical structure of hallucal metatarsals and locomotor adaptations in hominoids/24_F_A458_Soto_MT1L.0008.bmp]

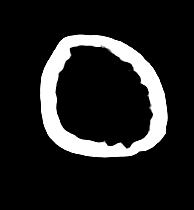

Supplement: S1 File — Individuals in central chimpanzee (Pan troglodytes troglodytes) and western lowland gorilla (Gorilla gorilla gorilla) are housed in the Primate Collection of the Department of Comparative Anatomy of the National Museum of Natural History, Paris, France (S1 Text). Humans (Homo sapiens) are housed in the Raymond A. Dart Collection of Human Skeletons at the University of the Witwatersrand, Johannesburg, South Africa (S1 Text) [70]. (ZIP) [file pone.0117905.s004.zip › X-Ray_Cortical structure of hallucal metatarsals and locomotor adaptations in hominoids/24_F_A458_Soto_MT1L.0009.bmp]

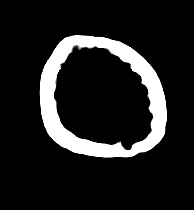

Supplement: S1 File — Individuals in central chimpanzee (Pan troglodytes troglodytes) and western lowland gorilla (Gorilla gorilla gorilla) are housed in the Primate Collection of the Department of Comparative Anatomy of the National Museum of Natural History, Paris, France (S1 Text). Humans (Homo sapiens) are housed in the Raymond A. Dart Collection of Human Skeletons at the University of the Witwatersrand, Johannesburg, South Africa (S1 Text) [70]. (ZIP) [file pone.0117905.s004.zip › X-Ray_Cortical structure of hallucal metatarsals and locomotor adaptations in hominoids/24_F_A458_Soto_MT1L.0010.bmp]

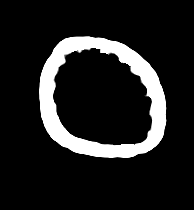

Supplement: S1 File — Individuals in central chimpanzee (Pan troglodytes troglodytes) and western lowland gorilla (Gorilla gorilla gorilla) are housed in the Primate Collection of the Department of Comparative Anatomy of the National Museum of Natural History, Paris, France (S1 Text). Humans (Homo sapiens) are housed in the Raymond A. Dart Collection of Human Skeletons at the University of the Witwatersrand, Johannesburg, South Africa (S1 Text) [70]. (ZIP) [file pone.0117905.s004.zip › X-Ray_Cortical structure of hallucal metatarsals and locomotor adaptations in hominoids/24_F_A458_Soto_MT1L.0011.bmp]

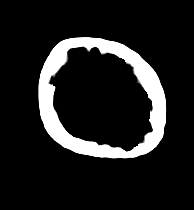

Supplement: S1 File — Individuals in central chimpanzee (Pan troglodytes troglodytes) and western lowland gorilla (Gorilla gorilla gorilla) are housed in the Primate Collection of the Department of Comparative Anatomy of the National Museum of Natural History, Paris, France (S1 Text). Humans (Homo sapiens) are housed in the Raymond A. Dart Collection of Human Skeletons at the University of the Witwatersrand, Johannesburg, South Africa (S1 Text) [70]. (ZIP) [file pone.0117905.s004.zip › X-Ray_Cortical structure of hallucal metatarsals and locomotor adaptations in hominoids/24_F_A458_Soto_MT1L.0012.bmp]

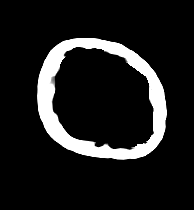

Supplement: S1 File — Individuals in central chimpanzee (Pan troglodytes troglodytes) and western lowland gorilla (Gorilla gorilla gorilla) are housed in the Primate Collection of the Department of Comparative Anatomy of the National Museum of Natural History, Paris, France (S1 Text). Humans (Homo sapiens) are housed in the Raymond A. Dart Collection of Human Skeletons at the University of the Witwatersrand, Johannesburg, South Africa (S1 Text) [70]. (ZIP) [file pone.0117905.s004.zip › X-Ray_Cortical structure of hallucal metatarsals and locomotor adaptations in hominoids/24_F_A458_Soto_MT1L.0013.bmp]

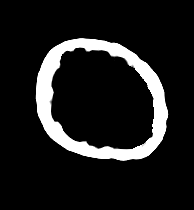

Supplement: S1 File — Individuals in central chimpanzee (Pan troglodytes troglodytes) and western lowland gorilla (Gorilla gorilla gorilla) are housed in the Primate Collection of the Department of Comparative Anatomy of the National Museum of Natural History, Paris, France (S1 Text). Humans (Homo sapiens) are housed in the Raymond A. Dart Collection of Human Skeletons at the University of the Witwatersrand, Johannesburg, South Africa (S1 Text) [70]. (ZIP) [file pone.0117905.s004.zip › X-Ray_Cortical structure of hallucal metatarsals and locomotor adaptations in hominoids/24_F_A458_Soto_MT1L.0014.bmp]

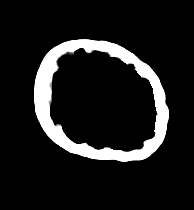

Supplement: S1 File — Individuals in central chimpanzee (Pan troglodytes troglodytes) and western lowland gorilla (Gorilla gorilla gorilla) are housed in the Primate Collection of the Department of Comparative Anatomy of the National Museum of Natural History, Paris, France (S1 Text). Humans (Homo sapiens) are housed in the Raymond A. Dart Collection of Human Skeletons at the University of the Witwatersrand, Johannesburg, South Africa (S1 Text) [70]. (ZIP) [file pone.0117905.s004.zip › X-Ray_Cortical structure of hallucal metatarsals and locomotor adaptations in hominoids/24_F_A458_Soto_MT1L.0015.bmp]

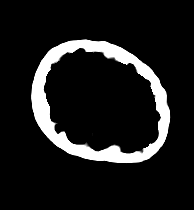

Supplement: S1 File — Individuals in central chimpanzee (Pan troglodytes troglodytes) and western lowland gorilla (Gorilla gorilla gorilla) are housed in the Primate Collection of the Department of Comparative Anatomy of the National Museum of Natural History, Paris, France (S1 Text). Humans (Homo sapiens) are housed in the Raymond A. Dart Collection of Human Skeletons at the University of the Witwatersrand, Johannesburg, South Africa (S1 Text) [70]. (ZIP) [file pone.0117905.s004.zip › X-Ray_Cortical structure of hallucal metatarsals and locomotor adaptations in hominoids/24_F_A458_Soto_MT1L.0016.bmp]

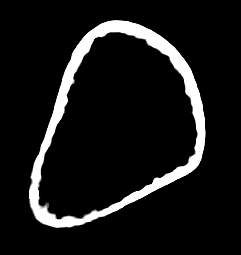

Supplement: S1 File — Individuals in central chimpanzee (Pan troglodytes troglodytes) and western lowland gorilla (Gorilla gorilla gorilla) are housed in the Primate Collection of the Department of Comparative Anatomy of the National Museum of Natural History, Paris, France (S1 Text). Humans (Homo sapiens) are housed in the Raymond A. Dart Collection of Human Skeletons at the University of the Witwatersrand, Johannesburg, South Africa (S1 Text) [70]. (ZIP) [file pone.0117905.s004.zip › X-Ray_Cortical structure of hallucal metatarsals and locomotor adaptations in hominoids/25_F_A209_Soto_MT1L.0000.bmp]

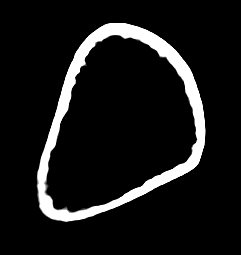

Supplement: S1 File — Individuals in central chimpanzee (Pan troglodytes troglodytes) and western lowland gorilla (Gorilla gorilla gorilla) are housed in the Primate Collection of the Department of Comparative Anatomy of the National Museum of Natural History, Paris, France (S1 Text). Humans (Homo sapiens) are housed in the Raymond A. Dart Collection of Human Skeletons at the University of the Witwatersrand, Johannesburg, South Africa (S1 Text) [70]. (ZIP) [file pone.0117905.s004.zip › X-Ray_Cortical structure of hallucal metatarsals and locomotor adaptations in hominoids/25_F_A209_Soto_MT1L.0001.bmp]

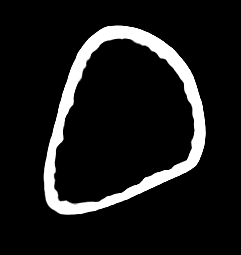

Supplement: S1 File — Individuals in central chimpanzee (Pan troglodytes troglodytes) and western lowland gorilla (Gorilla gorilla gorilla) are housed in the Primate Collection of the Department of Comparative Anatomy of the National Museum of Natural History, Paris, France (S1 Text). Humans (Homo sapiens) are housed in the Raymond A. Dart Collection of Human Skeletons at the University of the Witwatersrand, Johannesburg, South Africa (S1 Text) [70]. (ZIP) [file pone.0117905.s004.zip › X-Ray_Cortical structure of hallucal metatarsals and locomotor adaptations in hominoids/25_F_A209_Soto_MT1L.0002.bmp]

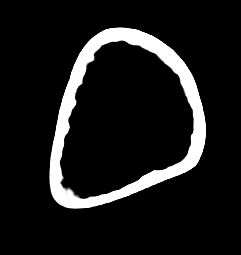

Supplement: S1 File — Individuals in central chimpanzee (Pan troglodytes troglodytes) and western lowland gorilla (Gorilla gorilla gorilla) are housed in the Primate Collection of the Department of Comparative Anatomy of the National Museum of Natural History, Paris, France (S1 Text). Humans (Homo sapiens) are housed in the Raymond A. Dart Collection of Human Skeletons at the University of the Witwatersrand, Johannesburg, South Africa (S1 Text) [70]. (ZIP) [file pone.0117905.s004.zip › X-Ray_Cortical structure of hallucal metatarsals and locomotor adaptations in hominoids/25_F_A209_Soto_MT1L.0003.bmp]

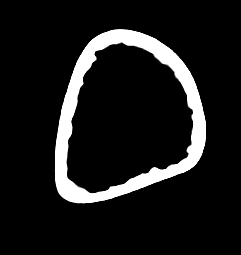

Supplement: S1 File — Individuals in central chimpanzee (Pan troglodytes troglodytes) and western lowland gorilla (Gorilla gorilla gorilla) are housed in the Primate Collection of the Department of Comparative Anatomy of the National Museum of Natural History, Paris, France (S1 Text). Humans (Homo sapiens) are housed in the Raymond A. Dart Collection of Human Skeletons at the University of the Witwatersrand, Johannesburg, South Africa (S1 Text) [70]. (ZIP) [file pone.0117905.s004.zip › X-Ray_Cortical structure of hallucal metatarsals and locomotor adaptations in hominoids/25_F_A209_Soto_MT1L.0004.bmp]

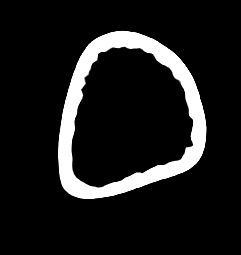

Supplement: S1 File — Individuals in central chimpanzee (Pan troglodytes troglodytes) and western lowland gorilla (Gorilla gorilla gorilla) are housed in the Primate Collection of the Department of Comparative Anatomy of the National Museum of Natural History, Paris, France (S1 Text). Humans (Homo sapiens) are housed in the Raymond A. Dart Collection of Human Skeletons at the University of the Witwatersrand, Johannesburg, South Africa (S1 Text) [70]. (ZIP) [file pone.0117905.s004.zip › X-Ray_Cortical structure of hallucal metatarsals and locomotor adaptations in hominoids/25_F_A209_Soto_MT1L.0005.bmp]

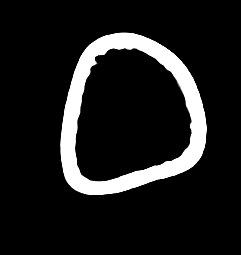

Supplement: S1 File — Individuals in central chimpanzee (Pan troglodytes troglodytes) and western lowland gorilla (Gorilla gorilla gorilla) are housed in the Primate Collection of the Department of Comparative Anatomy of the National Museum of Natural History, Paris, France (S1 Text). Humans (Homo sapiens) are housed in the Raymond A. Dart Collection of Human Skeletons at the University of the Witwatersrand, Johannesburg, South Africa (S1 Text) [70]. (ZIP) [file pone.0117905.s004.zip › X-Ray_Cortical structure of hallucal metatarsals and locomotor adaptations in hominoids/25_F_A209_Soto_MT1L.0006.bmp]

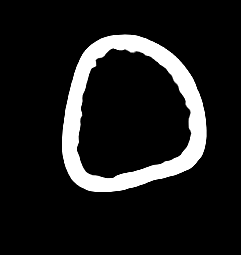

Supplement: S1 File — Individuals in central chimpanzee (Pan troglodytes troglodytes) and western lowland gorilla (Gorilla gorilla gorilla) are housed in the Primate Collection of the Department of Comparative Anatomy of the National Museum of Natural History, Paris, France (S1 Text). Humans (Homo sapiens) are housed in the Raymond A. Dart Collection of Human Skeletons at the University of the Witwatersrand, Johannesburg, South Africa (S1 Text) [70]. (ZIP) [file pone.0117905.s004.zip › X-Ray_Cortical structure of hallucal metatarsals and locomotor adaptations in hominoids/25_F_A209_Soto_MT1L.0007.bmp]

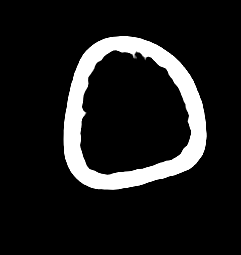

Supplement: S1 File — Individuals in central chimpanzee (Pan troglodytes troglodytes) and western lowland gorilla (Gorilla gorilla gorilla) are housed in the Primate Collection of the Department of Comparative Anatomy of the National Museum of Natural History, Paris, France (S1 Text). Humans (Homo sapiens) are housed in the Raymond A. Dart Collection of Human Skeletons at the University of the Witwatersrand, Johannesburg, South Africa (S1 Text) [70]. (ZIP) [file pone.0117905.s004.zip › X-Ray_Cortical structure of hallucal metatarsals and locomotor adaptations in hominoids/25_F_A209_Soto_MT1L.0008.bmp]

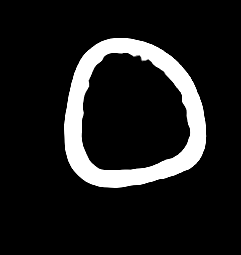

Supplement: S1 File — Individuals in central chimpanzee (Pan troglodytes troglodytes) and western lowland gorilla (Gorilla gorilla gorilla) are housed in the Primate Collection of the Department of Comparative Anatomy of the National Museum of Natural History, Paris, France (S1 Text). Humans (Homo sapiens) are housed in the Raymond A. Dart Collection of Human Skeletons at the University of the Witwatersrand, Johannesburg, South Africa (S1 Text) [70]. (ZIP) [file pone.0117905.s004.zip › X-Ray_Cortical structure of hallucal metatarsals and locomotor adaptations in hominoids/25_F_A209_Soto_MT1L.0009.bmp]

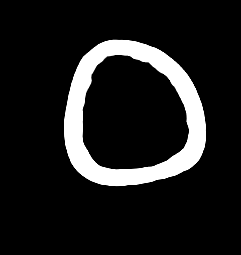

Supplement: S1 File — Individuals in central chimpanzee (Pan troglodytes troglodytes) and western lowland gorilla (Gorilla gorilla gorilla) are housed in the Primate Collection of the Department of Comparative Anatomy of the National Museum of Natural History, Paris, France (S1 Text). Humans (Homo sapiens) are housed in the Raymond A. Dart Collection of Human Skeletons at the University of the Witwatersrand, Johannesburg, South Africa (S1 Text) [70]. (ZIP) [file pone.0117905.s004.zip › X-Ray_Cortical structure of hallucal metatarsals and locomotor adaptations in hominoids/25_F_A209_Soto_MT1L.0010.bmp]

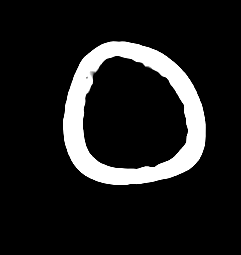

Supplement: S1 File — Individuals in central chimpanzee (Pan troglodytes troglodytes) and western lowland gorilla (Gorilla gorilla gorilla) are housed in the Primate Collection of the Department of Comparative Anatomy of the National Museum of Natural History, Paris, France (S1 Text). Humans (Homo sapiens) are housed in the Raymond A. Dart Collection of Human Skeletons at the University of the Witwatersrand, Johannesburg, South Africa (S1 Text) [70]. (ZIP) [file pone.0117905.s004.zip › X-Ray_Cortical structure of hallucal metatarsals and locomotor adaptations in hominoids/25_F_A209_Soto_MT1L.0011.bmp]

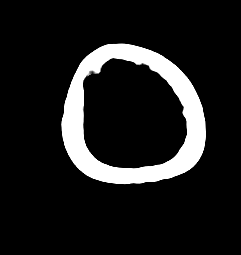

Supplement: S1 File — Individuals in central chimpanzee (Pan troglodytes troglodytes) and western lowland gorilla (Gorilla gorilla gorilla) are housed in the Primate Collection of the Department of Comparative Anatomy of the National Museum of Natural History, Paris, France (S1 Text). Humans (Homo sapiens) are housed in the Raymond A. Dart Collection of Human Skeletons at the University of the Witwatersrand, Johannesburg, South Africa (S1 Text) [70]. (ZIP) [file pone.0117905.s004.zip › X-Ray_Cortical structure of hallucal metatarsals and locomotor adaptations in hominoids/25_F_A209_Soto_MT1L.0012.bmp]

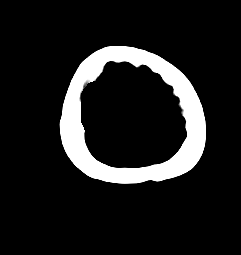

Supplement: S1 File — Individuals in central chimpanzee (Pan troglodytes troglodytes) and western lowland gorilla (Gorilla gorilla gorilla) are housed in the Primate Collection of the Department of Comparative Anatomy of the National Museum of Natural History, Paris, France (S1 Text). Humans (Homo sapiens) are housed in the Raymond A. Dart Collection of Human Skeletons at the University of the Witwatersrand, Johannesburg, South Africa (S1 Text) [70]. (ZIP) [file pone.0117905.s004.zip › X-Ray_Cortical structure of hallucal metatarsals and locomotor adaptations in hominoids/25_F_A209_Soto_MT1L.0013.bmp]

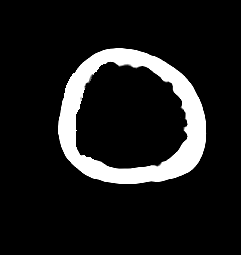

Supplement: S1 File — Individuals in central chimpanzee (Pan troglodytes troglodytes) and western lowland gorilla (Gorilla gorilla gorilla) are housed in the Primate Collection of the Department of Comparative Anatomy of the National Museum of Natural History, Paris, France (S1 Text). Humans (Homo sapiens) are housed in the Raymond A. Dart Collection of Human Skeletons at the University of the Witwatersrand, Johannesburg, South Africa (S1 Text) [70]. (ZIP) [file pone.0117905.s004.zip › X-Ray_Cortical structure of hallucal metatarsals and locomotor adaptations in hominoids/25_F_A209_Soto_MT1L.0014.bmp]

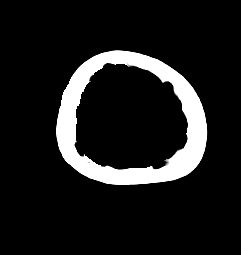

Supplement: S1 File — Individuals in central chimpanzee (Pan troglodytes troglodytes) and western lowland gorilla (Gorilla gorilla gorilla) are housed in the Primate Collection of the Department of Comparative Anatomy of the National Museum of Natural History, Paris, France (S1 Text). Humans (Homo sapiens) are housed in the Raymond A. Dart Collection of Human Skeletons at the University of the Witwatersrand, Johannesburg, South Africa (S1 Text) [70]. (ZIP) [file pone.0117905.s004.zip › X-Ray_Cortical structure of hallucal metatarsals and locomotor adaptations in hominoids/25_F_A209_Soto_MT1L.0015.bmp]

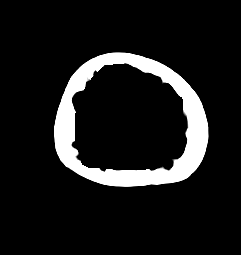

Supplement: S1 File — Individuals in central chimpanzee (Pan troglodytes troglodytes) and western lowland gorilla (Gorilla gorilla gorilla) are housed in the Primate Collection of the Department of Comparative Anatomy of the National Museum of Natural History, Paris, France (S1 Text). Humans (Homo sapiens) are housed in the Raymond A. Dart Collection of Human Skeletons at the University of the Witwatersrand, Johannesburg, South Africa (S1 Text) [70]. (ZIP) [file pone.0117905.s004.zip › X-Ray_Cortical structure of hallucal metatarsals and locomotor adaptations in hominoids/25_F_A209_Soto_MT1L.0016.bmp]

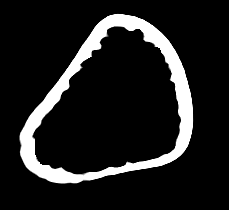

Supplement: S1 File — Individuals in central chimpanzee (Pan troglodytes troglodytes) and western lowland gorilla (Gorilla gorilla gorilla) are housed in the Primate Collection of the Department of Comparative Anatomy of the National Museum of Natural History, Paris, France (S1 Text). Humans (Homo sapiens) are housed in the Raymond A. Dart Collection of Human Skeletons at the University of the Witwatersrand, Johannesburg, South Africa (S1 Text) [70]. (ZIP) [file pone.0117905.s004.zip › X-Ray_Cortical structure of hallucal metatarsals and locomotor adaptations in hominoids/25_F_A218_Soto_MT1L.0000.bmp]

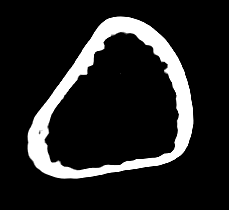

Supplement: S1 File — Individuals in central chimpanzee (Pan troglodytes troglodytes) and western lowland gorilla (Gorilla gorilla gorilla) are housed in the Primate Collection of the Department of Comparative Anatomy of the National Museum of Natural History, Paris, France (S1 Text). Humans (Homo sapiens) are housed in the Raymond A. Dart Collection of Human Skeletons at the University of the Witwatersrand, Johannesburg, South Africa (S1 Text) [70]. (ZIP) [file pone.0117905.s004.zip › X-Ray_Cortical structure of hallucal metatarsals and locomotor adaptations in hominoids/25_F_A218_Soto_MT1L.0001.bmp]

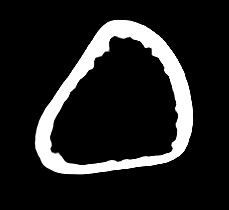

Supplement: S1 File — Individuals in central chimpanzee (Pan troglodytes troglodytes) and western lowland gorilla (Gorilla gorilla gorilla) are housed in the Primate Collection of the Department of Comparative Anatomy of the National Museum of Natural History, Paris, France (S1 Text). Humans (Homo sapiens) are housed in the Raymond A. Dart Collection of Human Skeletons at the University of the Witwatersrand, Johannesburg, South Africa (S1 Text) [70]. (ZIP) [file pone.0117905.s004.zip › X-Ray_Cortical structure of hallucal metatarsals and locomotor adaptations in hominoids/25_F_A218_Soto_MT1L.0002.bmp]

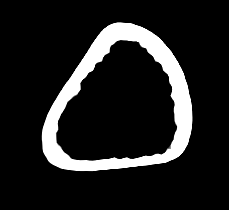

Supplement: S1 File — Individuals in central chimpanzee (Pan troglodytes troglodytes) and western lowland gorilla (Gorilla gorilla gorilla) are housed in the Primate Collection of the Department of Comparative Anatomy of the National Museum of Natural History, Paris, France (S1 Text). Humans (Homo sapiens) are housed in the Raymond A. Dart Collection of Human Skeletons at the University of the Witwatersrand, Johannesburg, South Africa (S1 Text) [70]. (ZIP) [file pone.0117905.s004.zip › X-Ray_Cortical structure of hallucal metatarsals and locomotor adaptations in hominoids/25_F_A218_Soto_MT1L.0003.bmp]

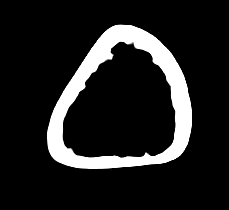

Supplement: S1 File — Individuals in central chimpanzee (Pan troglodytes troglodytes) and western lowland gorilla (Gorilla gorilla gorilla) are housed in the Primate Collection of the Department of Comparative Anatomy of the National Museum of Natural History, Paris, France (S1 Text). Humans (Homo sapiens) are housed in the Raymond A. Dart Collection of Human Skeletons at the University of the Witwatersrand, Johannesburg, South Africa (S1 Text) [70]. (ZIP) [file pone.0117905.s004.zip › X-Ray_Cortical structure of hallucal metatarsals and locomotor adaptations in hominoids/25_F_A218_Soto_MT1L.0004.bmp]

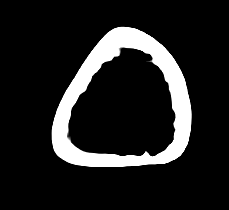

Supplement: S1 File — Individuals in central chimpanzee (Pan troglodytes troglodytes) and western lowland gorilla (Gorilla gorilla gorilla) are housed in the Primate Collection of the Department of Comparative Anatomy of the National Museum of Natural History, Paris, France (S1 Text). Humans (Homo sapiens) are housed in the Raymond A. Dart Collection of Human Skeletons at the University of the Witwatersrand, Johannesburg, South Africa (S1 Text) [70]. (ZIP) [file pone.0117905.s004.zip › X-Ray_Cortical structure of hallucal metatarsals and locomotor adaptations in hominoids/25_F_A218_Soto_MT1L.0005.bmp]

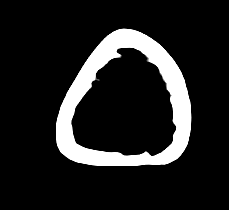

Supplement: S1 File — Individuals in central chimpanzee (Pan troglodytes troglodytes) and western lowland gorilla (Gorilla gorilla gorilla) are housed in the Primate Collection of the Department of Comparative Anatomy of the National Museum of Natural History, Paris, France (S1 Text). Humans (Homo sapiens) are housed in the Raymond A. Dart Collection of Human Skeletons at the University of the Witwatersrand, Johannesburg, South Africa (S1 Text) [70]. (ZIP) [file pone.0117905.s004.zip › X-Ray_Cortical structure of hallucal metatarsals and locomotor adaptations in hominoids/25_F_A218_Soto_MT1L.0006.bmp]

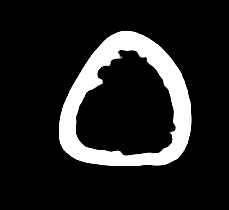

Supplement: S1 File — Individuals in central chimpanzee (Pan troglodytes troglodytes) and western lowland gorilla (Gorilla gorilla gorilla) are housed in the Primate Collection of the Department of Comparative Anatomy of the National Museum of Natural History, Paris, France (S1 Text). Humans (Homo sapiens) are housed in the Raymond A. Dart Collection of Human Skeletons at the University of the Witwatersrand, Johannesburg, South Africa (S1 Text) [70]. (ZIP) [file pone.0117905.s004.zip › X-Ray_Cortical structure of hallucal metatarsals and locomotor adaptations in hominoids/25_F_A218_Soto_MT1L.0007.bmp]

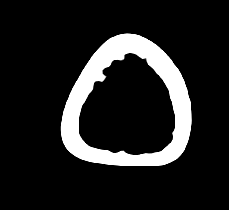

Supplement: S1 File — Individuals in central chimpanzee (Pan troglodytes troglodytes) and western lowland gorilla (Gorilla gorilla gorilla) are housed in the Primate Collection of the Department of Comparative Anatomy of the National Museum of Natural History, Paris, France (S1 Text). Humans (Homo sapiens) are housed in the Raymond A. Dart Collection of Human Skeletons at the University of the Witwatersrand, Johannesburg, South Africa (S1 Text) [70]. (ZIP) [file pone.0117905.s004.zip › X-Ray_Cortical structure of hallucal metatarsals and locomotor adaptations in hominoids/25_F_A218_Soto_MT1L.0008.bmp]

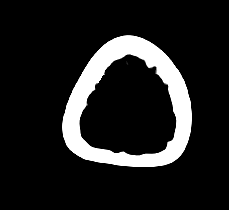

Supplement: S1 File — Individuals in central chimpanzee (Pan troglodytes troglodytes) and western lowland gorilla (Gorilla gorilla gorilla) are housed in the Primate Collection of the Department of Comparative Anatomy of the National Museum of Natural History, Paris, France (S1 Text). Humans (Homo sapiens) are housed in the Raymond A. Dart Collection of Human Skeletons at the University of the Witwatersrand, Johannesburg, South Africa (S1 Text) [70]. (ZIP) [file pone.0117905.s004.zip › X-Ray_Cortical structure of hallucal metatarsals and locomotor adaptations in hominoids/25_F_A218_Soto_MT1L.0009.bmp]

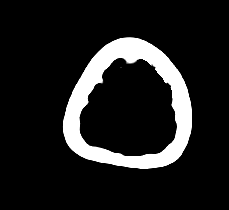

Supplement: S1 File — Individuals in central chimpanzee (Pan troglodytes troglodytes) and western lowland gorilla (Gorilla gorilla gorilla) are housed in the Primate Collection of the Department of Comparative Anatomy of the National Museum of Natural History, Paris, France (S1 Text). Humans (Homo sapiens) are housed in the Raymond A. Dart Collection of Human Skeletons at the University of the Witwatersrand, Johannesburg, South Africa (S1 Text) [70]. (ZIP) [file pone.0117905.s004.zip › X-Ray_Cortical structure of hallucal metatarsals and locomotor adaptations in hominoids/25_F_A218_Soto_MT1L.0010.bmp]

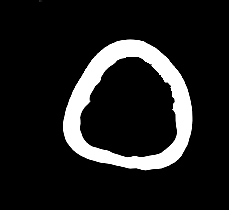

Supplement: S1 File — Individuals in central chimpanzee (Pan troglodytes troglodytes) and western lowland gorilla (Gorilla gorilla gorilla) are housed in the Primate Collection of the Department of Comparative Anatomy of the National Museum of Natural History, Paris, France (S1 Text). Humans (Homo sapiens) are housed in the Raymond A. Dart Collection of Human Skeletons at the University of the Witwatersrand, Johannesburg, South Africa (S1 Text) [70]. (ZIP) [file pone.0117905.s004.zip › X-Ray_Cortical structure of hallucal metatarsals and locomotor adaptations in hominoids/25_F_A218_Soto_MT1L.0011.bmp]

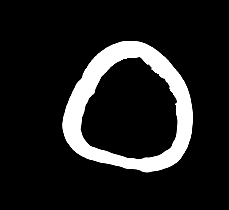

Supplement: S1 File — Individuals in central chimpanzee (Pan troglodytes troglodytes) and western lowland gorilla (Gorilla gorilla gorilla) are housed in the Primate Collection of the Department of Comparative Anatomy of the National Museum of Natural History, Paris, France (S1 Text). Humans (Homo sapiens) are housed in the Raymond A. Dart Collection of Human Skeletons at the University of the Witwatersrand, Johannesburg, South Africa (S1 Text) [70]. (ZIP) [file pone.0117905.s004.zip › X-Ray_Cortical structure of hallucal metatarsals and locomotor adaptations in hominoids/25_F_A218_Soto_MT1L.0012.bmp]

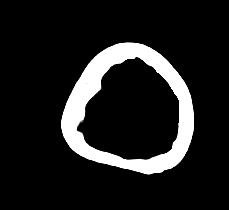

Supplement: S1 File — Individuals in central chimpanzee (Pan troglodytes troglodytes) and western lowland gorilla (Gorilla gorilla gorilla) are housed in the Primate Collection of the Department of Comparative Anatomy of the National Museum of Natural History, Paris, France (S1 Text). Humans (Homo sapiens) are housed in the Raymond A. Dart Collection of Human Skeletons at the University of the Witwatersrand, Johannesburg, South Africa (S1 Text) [70]. (ZIP) [file pone.0117905.s004.zip › X-Ray_Cortical structure of hallucal metatarsals and locomotor adaptations in hominoids/25_F_A218_Soto_MT1L.0013.bmp]

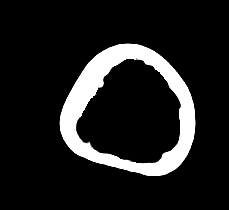

Supplement: S1 File — Individuals in central chimpanzee (Pan troglodytes troglodytes) and western lowland gorilla (Gorilla gorilla gorilla) are housed in the Primate Collection of the Department of Comparative Anatomy of the National Museum of Natural History, Paris, France (S1 Text). Humans (Homo sapiens) are housed in the Raymond A. Dart Collection of Human Skeletons at the University of the Witwatersrand, Johannesburg, South Africa (S1 Text) [70]. (ZIP) [file pone.0117905.s004.zip › X-Ray_Cortical structure of hallucal metatarsals and locomotor adaptations in hominoids/25_F_A218_Soto_MT1L.0014.bmp]

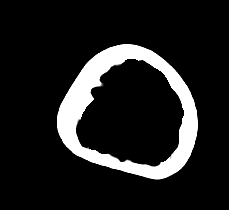

Supplement: S1 File — Individuals in central chimpanzee (Pan troglodytes troglodytes) and western lowland gorilla (Gorilla gorilla gorilla) are housed in the Primate Collection of the Department of Comparative Anatomy of the National Museum of Natural History, Paris, France (S1 Text). Humans (Homo sapiens) are housed in the Raymond A. Dart Collection of Human Skeletons at the University of the Witwatersrand, Johannesburg, South Africa (S1 Text) [70]. (ZIP) [file pone.0117905.s004.zip › X-Ray_Cortical structure of hallucal metatarsals and locomotor adaptations in hominoids/25_F_A218_Soto_MT1L.0015.bmp]

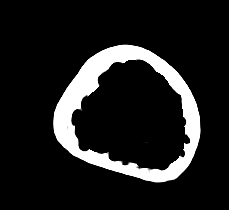

Supplement: S1 File — Individuals in central chimpanzee (Pan troglodytes troglodytes) and western lowland gorilla (Gorilla gorilla gorilla) are housed in the Primate Collection of the Department of Comparative Anatomy of the National Museum of Natural History, Paris, France (S1 Text). Humans (Homo sapiens) are housed in the Raymond A. Dart Collection of Human Skeletons at the University of the Witwatersrand, Johannesburg, South Africa (S1 Text) [70]. (ZIP) [file pone.0117905.s004.zip › X-Ray_Cortical structure of hallucal metatarsals and locomotor adaptations in hominoids/25_F_A218_Soto_MT1L.0016.bmp]

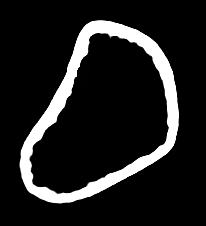

Supplement: S1 File — Individuals in central chimpanzee (Pan troglodytes troglodytes) and western lowland gorilla (Gorilla gorilla gorilla) are housed in the Primate Collection of the Department of Comparative Anatomy of the National Museum of Natural History, Paris, France (S1 Text). Humans (Homo sapiens) are housed in the Raymond A. Dart Collection of Human Skeletons at the University of the Witwatersrand, Johannesburg, South Africa (S1 Text) [70]. (ZIP) [file pone.0117905.s004.zip › X-Ray_Cortical structure of hallucal metatarsals and locomotor adaptations in hominoids/25_M_A14_Zulu_MT1.0000.bmp]

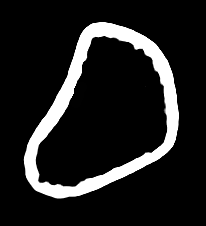

Supplement: S1 File — Individuals in central chimpanzee (Pan troglodytes troglodytes) and western lowland gorilla (Gorilla gorilla gorilla) are housed in the Primate Collection of the Department of Comparative Anatomy of the National Museum of Natural History, Paris, France (S1 Text). Humans (Homo sapiens) are housed in the Raymond A. Dart Collection of Human Skeletons at the University of the Witwatersrand, Johannesburg, South Africa (S1 Text) [70]. (ZIP) [file pone.0117905.s004.zip › X-Ray_Cortical structure of hallucal metatarsals and locomotor adaptations in hominoids/25_M_A14_Zulu_MT1.0001.bmp]

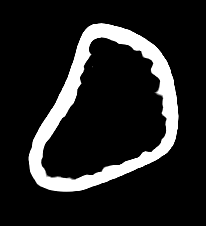

Supplement: S1 File — Individuals in central chimpanzee (Pan troglodytes troglodytes) and western lowland gorilla (Gorilla gorilla gorilla) are housed in the Primate Collection of the Department of Comparative Anatomy of the National Museum of Natural History, Paris, France (S1 Text). Humans (Homo sapiens) are housed in the Raymond A. Dart Collection of Human Skeletons at the University of the Witwatersrand, Johannesburg, South Africa (S1 Text) [70]. (ZIP) [file pone.0117905.s004.zip › X-Ray_Cortical structure of hallucal metatarsals and locomotor adaptations in hominoids/25_M_A14_Zulu_MT1.0002.bmp]

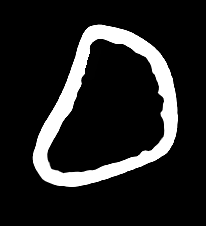

Supplement: S1 File — Individuals in central chimpanzee (Pan troglodytes troglodytes) and western lowland gorilla (Gorilla gorilla gorilla) are housed in the Primate Collection of the Department of Comparative Anatomy of the National Museum of Natural History, Paris, France (S1 Text). Humans (Homo sapiens) are housed in the Raymond A. Dart Collection of Human Skeletons at the University of the Witwatersrand, Johannesburg, South Africa (S1 Text) [70]. (ZIP) [file pone.0117905.s004.zip › X-Ray_Cortical structure of hallucal metatarsals and locomotor adaptations in hominoids/25_M_A14_Zulu_MT1.0003.bmp]

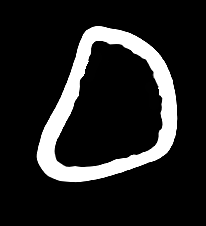

Supplement: S1 File — Individuals in central chimpanzee (Pan troglodytes troglodytes) and western lowland gorilla (Gorilla gorilla gorilla) are housed in the Primate Collection of the Department of Comparative Anatomy of the National Museum of Natural History, Paris, France (S1 Text). Humans (Homo sapiens) are housed in the Raymond A. Dart Collection of Human Skeletons at the University of the Witwatersrand, Johannesburg, South Africa (S1 Text) [70]. (ZIP) [file pone.0117905.s004.zip › X-Ray_Cortical structure of hallucal metatarsals and locomotor adaptations in hominoids/25_M_A14_Zulu_MT1.0004.bmp]

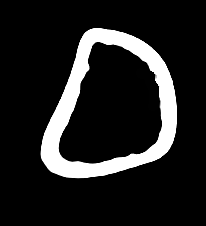

Supplement: S1 File — Individuals in central chimpanzee (Pan troglodytes troglodytes) and western lowland gorilla (Gorilla gorilla gorilla) are housed in the Primate Collection of the Department of Comparative Anatomy of the National Museum of Natural History, Paris, France (S1 Text). Humans (Homo sapiens) are housed in the Raymond A. Dart Collection of Human Skeletons at the University of the Witwatersrand, Johannesburg, South Africa (S1 Text) [70]. (ZIP) [file pone.0117905.s004.zip › X-Ray_Cortical structure of hallucal metatarsals and locomotor adaptations in hominoids/25_M_A14_Zulu_MT1.0005.bmp]

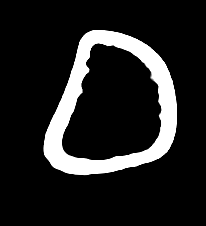

Supplement: S1 File — Individuals in central chimpanzee (Pan troglodytes troglodytes) and western lowland gorilla (Gorilla gorilla gorilla) are housed in the Primate Collection of the Department of Comparative Anatomy of the National Museum of Natural History, Paris, France (S1 Text). Humans (Homo sapiens) are housed in the Raymond A. Dart Collection of Human Skeletons at the University of the Witwatersrand, Johannesburg, South Africa (S1 Text) [70]. (ZIP) [file pone.0117905.s004.zip › X-Ray_Cortical structure of hallucal metatarsals and locomotor adaptations in hominoids/25_M_A14_Zulu_MT1.0006.bmp]

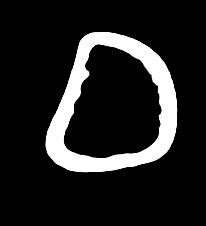

Supplement: S1 File — Individuals in central chimpanzee (Pan troglodytes troglodytes) and western lowland gorilla (Gorilla gorilla gorilla) are housed in the Primate Collection of the Department of Comparative Anatomy of the National Museum of Natural History, Paris, France (S1 Text). Humans (Homo sapiens) are housed in the Raymond A. Dart Collection of Human Skeletons at the University of the Witwatersrand, Johannesburg, South Africa (S1 Text) [70]. (ZIP) [file pone.0117905.s004.zip › X-Ray_Cortical structure of hallucal metatarsals and locomotor adaptations in hominoids/25_M_A14_Zulu_MT1.0007.bmp]

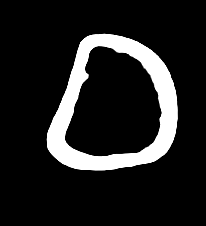

Supplement: S1 File — Individuals in central chimpanzee (Pan troglodytes troglodytes) and western lowland gorilla (Gorilla gorilla gorilla) are housed in the Primate Collection of the Department of Comparative Anatomy of the National Museum of Natural History, Paris, France (S1 Text). Humans (Homo sapiens) are housed in the Raymond A. Dart Collection of Human Skeletons at the University of the Witwatersrand, Johannesburg, South Africa (S1 Text) [70]. (ZIP) [file pone.0117905.s004.zip › X-Ray_Cortical structure of hallucal metatarsals and locomotor adaptations in hominoids/25_M_A14_Zulu_MT1.0008.bmp]

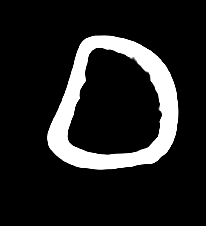

Supplement: S1 File — Individuals in central chimpanzee (Pan troglodytes troglodytes) and western lowland gorilla (Gorilla gorilla gorilla) are housed in the Primate Collection of the Department of Comparative Anatomy of the National Museum of Natural History, Paris, France (S1 Text). Humans (Homo sapiens) are housed in the Raymond A. Dart Collection of Human Skeletons at the University of the Witwatersrand, Johannesburg, South Africa (S1 Text) [70]. (ZIP) [file pone.0117905.s004.zip › X-Ray_Cortical structure of hallucal metatarsals and locomotor adaptations in hominoids/25_M_A14_Zulu_MT1.0009.bmp]

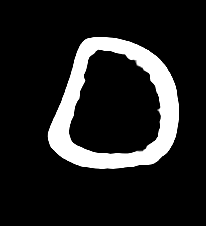

Supplement: S1 File — Individuals in central chimpanzee (Pan troglodytes troglodytes) and western lowland gorilla (Gorilla gorilla gorilla) are housed in the Primate Collection of the Department of Comparative Anatomy of the National Museum of Natural History, Paris, France (S1 Text). Humans (Homo sapiens) are housed in the Raymond A. Dart Collection of Human Skeletons at the University of the Witwatersrand, Johannesburg, South Africa (S1 Text) [70]. (ZIP) [file pone.0117905.s004.zip › X-Ray_Cortical structure of hallucal metatarsals and locomotor adaptations in hominoids/25_M_A14_Zulu_MT1.0010.bmp]

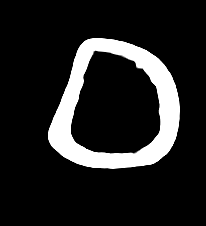

Supplement: S1 File — Individuals in central chimpanzee (Pan troglodytes troglodytes) and western lowland gorilla (Gorilla gorilla gorilla) are housed in the Primate Collection of the Department of Comparative Anatomy of the National Museum of Natural History, Paris, France (S1 Text). Humans (Homo sapiens) are housed in the Raymond A. Dart Collection of Human Skeletons at the University of the Witwatersrand, Johannesburg, South Africa (S1 Text) [70]. (ZIP) [file pone.0117905.s004.zip › X-Ray_Cortical structure of hallucal metatarsals and locomotor adaptations in hominoids/25_M_A14_Zulu_MT1.0011.bmp]

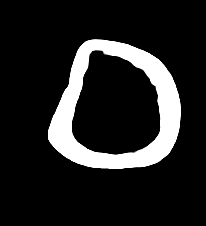

Supplement: S1 File — Individuals in central chimpanzee (Pan troglodytes troglodytes) and western lowland gorilla (Gorilla gorilla gorilla) are housed in the Primate Collection of the Department of Comparative Anatomy of the National Museum of Natural History, Paris, France (S1 Text). Humans (Homo sapiens) are housed in the Raymond A. Dart Collection of Human Skeletons at the University of the Witwatersrand, Johannesburg, South Africa (S1 Text) [70]. (ZIP) [file pone.0117905.s004.zip › X-Ray_Cortical structure of hallucal metatarsals and locomotor adaptations in hominoids/25_M_A14_Zulu_MT1.0012.bmp]

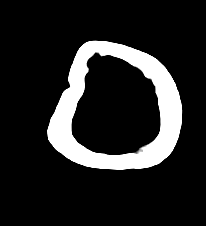

Supplement: S1 File — Individuals in central chimpanzee (Pan troglodytes troglodytes) and western lowland gorilla (Gorilla gorilla gorilla) are housed in the Primate Collection of the Department of Comparative Anatomy of the National Museum of Natural History, Paris, France (S1 Text). Humans (Homo sapiens) are housed in the Raymond A. Dart Collection of Human Skeletons at the University of the Witwatersrand, Johannesburg, South Africa (S1 Text) [70]. (ZIP) [file pone.0117905.s004.zip › X-Ray_Cortical structure of hallucal metatarsals and locomotor adaptations in hominoids/25_M_A14_Zulu_MT1.0013.bmp]

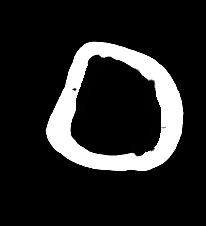

Supplement: S1 File — Individuals in central chimpanzee (Pan troglodytes troglodytes) and western lowland gorilla (Gorilla gorilla gorilla) are housed in the Primate Collection of the Department of Comparative Anatomy of the National Museum of Natural History, Paris, France (S1 Text). Humans (Homo sapiens) are housed in the Raymond A. Dart Collection of Human Skeletons at the University of the Witwatersrand, Johannesburg, South Africa (S1 Text) [70]. (ZIP) [file pone.0117905.s004.zip › X-Ray_Cortical structure of hallucal metatarsals and locomotor adaptations in hominoids/25_M_A14_Zulu_MT1.0014.bmp]

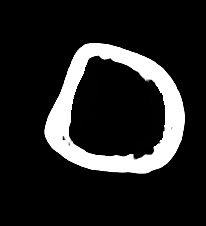

Supplement: S1 File — Individuals in central chimpanzee (Pan troglodytes troglodytes) and western lowland gorilla (Gorilla gorilla gorilla) are housed in the Primate Collection of the Department of Comparative Anatomy of the National Museum of Natural History, Paris, France (S1 Text). Humans (Homo sapiens) are housed in the Raymond A. Dart Collection of Human Skeletons at the University of the Witwatersrand, Johannesburg, South Africa (S1 Text) [70]. (ZIP) [file pone.0117905.s004.zip › X-Ray_Cortical structure of hallucal metatarsals and locomotor adaptations in hominoids/25_M_A14_Zulu_MT1.0015.bmp]

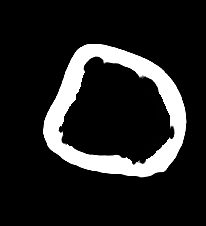

Supplement: S1 File — Individuals in central chimpanzee (Pan troglodytes troglodytes) and western lowland gorilla (Gorilla gorilla gorilla) are housed in the Primate Collection of the Department of Comparative Anatomy of the National Museum of Natural History, Paris, France (S1 Text). Humans (Homo sapiens) are housed in the Raymond A. Dart Collection of Human Skeletons at the University of the Witwatersrand, Johannesburg, South Africa (S1 Text) [70]. (ZIP) [file pone.0117905.s004.zip › X-Ray_Cortical structure of hallucal metatarsals and locomotor adaptations in hominoids/25_M_A14_Zulu_MT1.0016.bmp]

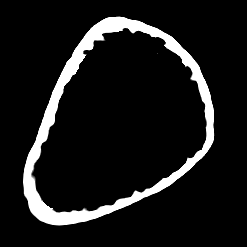

Supplement: S1 File — Individuals in central chimpanzee (Pan troglodytes troglodytes) and western lowland gorilla (Gorilla gorilla gorilla) are housed in the Primate Collection of the Department of Comparative Anatomy of the National Museum of Natural History, Paris, France (S1 Text). Humans (Homo sapiens) are housed in the Raymond A. Dart Collection of Human Skeletons at the University of the Witwatersrand, Johannesburg, South Africa (S1 Text) [70]. (ZIP) [file pone.0117905.s004.zip › X-Ray_Cortical structure of hallucal metatarsals and locomotor adaptations in hominoids/25_M_A163_Soto_MT1L.0000.bmp]

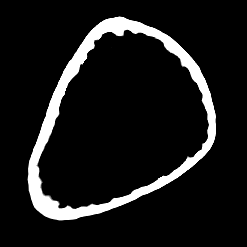

Supplement: S1 File — Individuals in central chimpanzee (Pan troglodytes troglodytes) and western lowland gorilla (Gorilla gorilla gorilla) are housed in the Primate Collection of the Department of Comparative Anatomy of the National Museum of Natural History, Paris, France (S1 Text). Humans (Homo sapiens) are housed in the Raymond A. Dart Collection of Human Skeletons at the University of the Witwatersrand, Johannesburg, South Africa (S1 Text) [70]. (ZIP) [file pone.0117905.s004.zip › X-Ray_Cortical structure of hallucal metatarsals and locomotor adaptations in hominoids/25_M_A163_Soto_MT1L.0001.bmp]

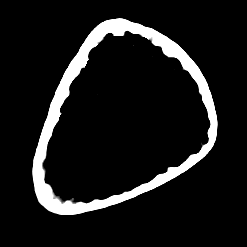

Supplement: S1 File — Individuals in central chimpanzee (Pan troglodytes troglodytes) and western lowland gorilla (Gorilla gorilla gorilla) are housed in the Primate Collection of the Department of Comparative Anatomy of the National Museum of Natural History, Paris, France (S1 Text). Humans (Homo sapiens) are housed in the Raymond A. Dart Collection of Human Skeletons at the University of the Witwatersrand, Johannesburg, South Africa (S1 Text) [70]. (ZIP) [file pone.0117905.s004.zip › X-Ray_Cortical structure of hallucal metatarsals and locomotor adaptations in hominoids/25_M_A163_Soto_MT1L.0002.bmp]

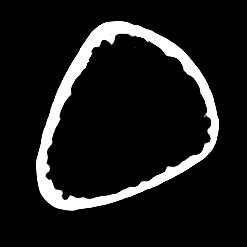

Supplement: S1 File — Individuals in central chimpanzee (Pan troglodytes troglodytes) and western lowland gorilla (Gorilla gorilla gorilla) are housed in the Primate Collection of the Department of Comparative Anatomy of the National Museum of Natural History, Paris, France (S1 Text). Humans (Homo sapiens) are housed in the Raymond A. Dart Collection of Human Skeletons at the University of the Witwatersrand, Johannesburg, South Africa (S1 Text) [70]. (ZIP) [file pone.0117905.s004.zip › X-Ray_Cortical structure of hallucal metatarsals and locomotor adaptations in hominoids/25_M_A163_Soto_MT1L.0003.bmp]

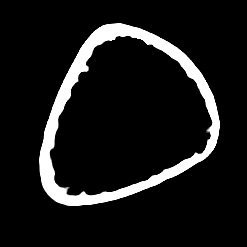

Supplement: S1 File — Individuals in central chimpanzee (Pan troglodytes troglodytes) and western lowland gorilla (Gorilla gorilla gorilla) are housed in the Primate Collection of the Department of Comparative Anatomy of the National Museum of Natural History, Paris, France (S1 Text). Humans (Homo sapiens) are housed in the Raymond A. Dart Collection of Human Skeletons at the University of the Witwatersrand, Johannesburg, South Africa (S1 Text) [70]. (ZIP) [file pone.0117905.s004.zip › X-Ray_Cortical structure of hallucal metatarsals and locomotor adaptations in hominoids/25_M_A163_Soto_MT1L.0004.bmp]

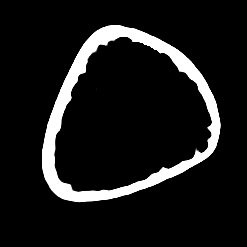

Supplement: S1 File — Individuals in central chimpanzee (Pan troglodytes troglodytes) and western lowland gorilla (Gorilla gorilla gorilla) are housed in the Primate Collection of the Department of Comparative Anatomy of the National Museum of Natural History, Paris, France (S1 Text). Humans (Homo sapiens) are housed in the Raymond A. Dart Collection of Human Skeletons at the University of the Witwatersrand, Johannesburg, South Africa (S1 Text) [70]. (ZIP) [file pone.0117905.s004.zip › X-Ray_Cortical structure of hallucal metatarsals and locomotor adaptations in hominoids/25_M_A163_Soto_MT1L.0005.bmp]

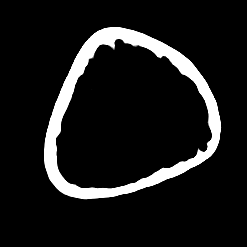

Supplement: S1 File — Individuals in central chimpanzee (Pan troglodytes troglodytes) and western lowland gorilla (Gorilla gorilla gorilla) are housed in the Primate Collection of the Department of Comparative Anatomy of the National Museum of Natural History, Paris, France (S1 Text). Humans (Homo sapiens) are housed in the Raymond A. Dart Collection of Human Skeletons at the University of the Witwatersrand, Johannesburg, South Africa (S1 Text) [70]. (ZIP) [file pone.0117905.s004.zip › X-Ray_Cortical structure of hallucal metatarsals and locomotor adaptations in hominoids/25_M_A163_Soto_MT1L.0006.bmp]

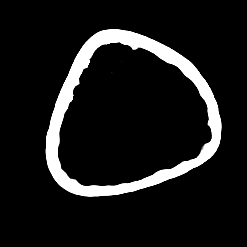

Supplement: S1 File — Individuals in central chimpanzee (Pan troglodytes troglodytes) and western lowland gorilla (Gorilla gorilla gorilla) are housed in the Primate Collection of the Department of Comparative Anatomy of the National Museum of Natural History, Paris, France (S1 Text). Humans (Homo sapiens) are housed in the Raymond A. Dart Collection of Human Skeletons at the University of the Witwatersrand, Johannesburg, South Africa (S1 Text) [70]. (ZIP) [file pone.0117905.s004.zip › X-Ray_Cortical structure of hallucal metatarsals and locomotor adaptations in hominoids/25_M_A163_Soto_MT1L.0007.bmp]

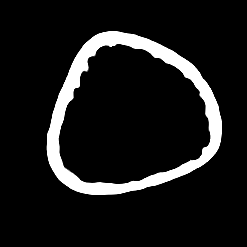

Supplement: S1 File — Individuals in central chimpanzee (Pan troglodytes troglodytes) and western lowland gorilla (Gorilla gorilla gorilla) are housed in the Primate Collection of the Department of Comparative Anatomy of the National Museum of Natural History, Paris, France (S1 Text). Humans (Homo sapiens) are housed in the Raymond A. Dart Collection of Human Skeletons at the University of the Witwatersrand, Johannesburg, South Africa (S1 Text) [70]. (ZIP) [file pone.0117905.s004.zip › X-Ray_Cortical structure of hallucal metatarsals and locomotor adaptations in hominoids/25_M_A163_Soto_MT1L.0008.bmp]

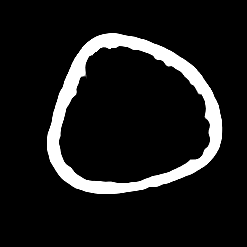

Supplement: S1 File — Individuals in central chimpanzee (Pan troglodytes troglodytes) and western lowland gorilla (Gorilla gorilla gorilla) are housed in the Primate Collection of the Department of Comparative Anatomy of the National Museum of Natural History, Paris, France (S1 Text). Humans (Homo sapiens) are housed in the Raymond A. Dart Collection of Human Skeletons at the University of the Witwatersrand, Johannesburg, South Africa (S1 Text) [70]. (ZIP) [file pone.0117905.s004.zip › X-Ray_Cortical structure of hallucal metatarsals and locomotor adaptations in hominoids/25_M_A163_Soto_MT1L.0009.bmp]

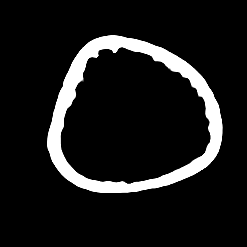

Supplement: S1 File — Individuals in central chimpanzee (Pan troglodytes troglodytes) and western lowland gorilla (Gorilla gorilla gorilla) are housed in the Primate Collection of the Department of Comparative Anatomy of the National Museum of Natural History, Paris, France (S1 Text). Humans (Homo sapiens) are housed in the Raymond A. Dart Collection of Human Skeletons at the University of the Witwatersrand, Johannesburg, South Africa (S1 Text) [70]. (ZIP) [file pone.0117905.s004.zip › X-Ray_Cortical structure of hallucal metatarsals and locomotor adaptations in hominoids/25_M_A163_Soto_MT1L.0010.bmp]

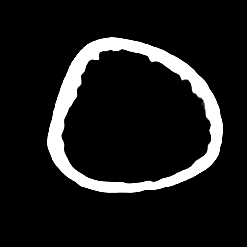

Supplement: S1 File — Individuals in central chimpanzee (Pan troglodytes troglodytes) and western lowland gorilla (Gorilla gorilla gorilla) are housed in the Primate Collection of the Department of Comparative Anatomy of the National Museum of Natural History, Paris, France (S1 Text). Humans (Homo sapiens) are housed in the Raymond A. Dart Collection of Human Skeletons at the University of the Witwatersrand, Johannesburg, South Africa (S1 Text) [70]. (ZIP) [file pone.0117905.s004.zip › X-Ray_Cortical structure of hallucal metatarsals and locomotor adaptations in hominoids/25_M_A163_Soto_MT1L.0011.bmp]
