# Supplementary figures and images for: Cortical Structure of Hallucal Metatarsals and Locomotor Adaptations in Hominoids (part 2 of 3)
Source: PLoS One. 2015 Jan 30;10(1):e0117905. doi: 10.1371/journal.pone.0117905 (PMC4311976; doi:10.1371/journal.pone.0117905)

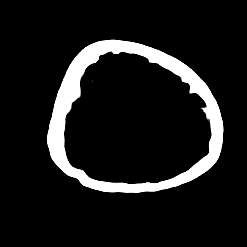

Supplement: S1 File — Individuals in central chimpanzee (Pan troglodytes troglodytes) and western lowland gorilla (Gorilla gorilla gorilla) are housed in the Primate Collection of the Department of Comparative Anatomy of the National Museum of Natural History, Paris, France (S1 Text). Humans (Homo sapiens) are housed in the Raymond A. Dart Collection of Human Skeletons at the University of the Witwatersrand, Johannesburg, South Africa (S1 Text) [70]. (ZIP) [file pone.0117905.s004.zip › X-Ray_Cortical structure of hallucal metatarsals and locomotor adaptations in hominoids/25_M_A163_Soto_MT1L.0012.bmp]

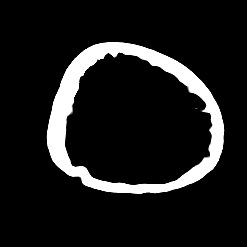

Supplement: S1 File — Individuals in central chimpanzee (Pan troglodytes troglodytes) and western lowland gorilla (Gorilla gorilla gorilla) are housed in the Primate Collection of the Department of Comparative Anatomy of the National Museum of Natural History, Paris, France (S1 Text). Humans (Homo sapiens) are housed in the Raymond A. Dart Collection of Human Skeletons at the University of the Witwatersrand, Johannesburg, South Africa (S1 Text) [70]. (ZIP) [file pone.0117905.s004.zip › X-Ray_Cortical structure of hallucal metatarsals and locomotor adaptations in hominoids/25_M_A163_Soto_MT1L.0013.bmp]

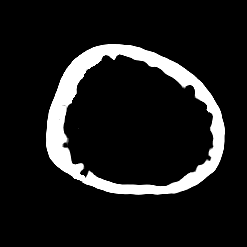

Supplement: S1 File — Individuals in central chimpanzee (Pan troglodytes troglodytes) and western lowland gorilla (Gorilla gorilla gorilla) are housed in the Primate Collection of the Department of Comparative Anatomy of the National Museum of Natural History, Paris, France (S1 Text). Humans (Homo sapiens) are housed in the Raymond A. Dart Collection of Human Skeletons at the University of the Witwatersrand, Johannesburg, South Africa (S1 Text) [70]. (ZIP) [file pone.0117905.s004.zip › X-Ray_Cortical structure of hallucal metatarsals and locomotor adaptations in hominoids/25_M_A163_Soto_MT1L.0014.bmp]

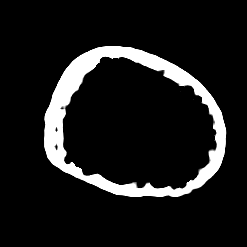

Supplement: S1 File — Individuals in central chimpanzee (Pan troglodytes troglodytes) and western lowland gorilla (Gorilla gorilla gorilla) are housed in the Primate Collection of the Department of Comparative Anatomy of the National Museum of Natural History, Paris, France (S1 Text). Humans (Homo sapiens) are housed in the Raymond A. Dart Collection of Human Skeletons at the University of the Witwatersrand, Johannesburg, South Africa (S1 Text) [70]. (ZIP) [file pone.0117905.s004.zip › X-Ray_Cortical structure of hallucal metatarsals and locomotor adaptations in hominoids/25_M_A163_Soto_MT1L.0015.bmp]

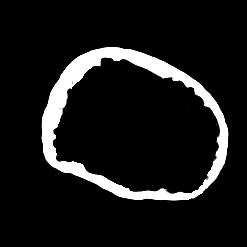

Supplement: S1 File — Individuals in central chimpanzee (Pan troglodytes troglodytes) and western lowland gorilla (Gorilla gorilla gorilla) are housed in the Primate Collection of the Department of Comparative Anatomy of the National Museum of Natural History, Paris, France (S1 Text). Humans (Homo sapiens) are housed in the Raymond A. Dart Collection of Human Skeletons at the University of the Witwatersrand, Johannesburg, South Africa (S1 Text) [70]. (ZIP) [file pone.0117905.s004.zip › X-Ray_Cortical structure of hallucal metatarsals and locomotor adaptations in hominoids/25_M_A163_Soto_MT1L.0016.bmp]

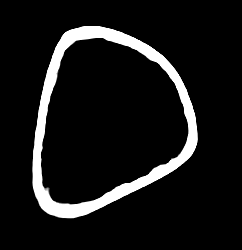

Supplement: S1 File — Individuals in central chimpanzee (Pan troglodytes troglodytes) and western lowland gorilla (Gorilla gorilla gorilla) are housed in the Primate Collection of the Department of Comparative Anatomy of the National Museum of Natural History, Paris, France (S1 Text). Humans (Homo sapiens) are housed in the Raymond A. Dart Collection of Human Skeletons at the University of the Witwatersrand, Johannesburg, South Africa (S1 Text) [70]. (ZIP) [file pone.0117905.s004.zip › X-Ray_Cortical structure of hallucal metatarsals and locomotor adaptations in hominoids/26_M_A17_Soto_MT1L.0000.bmp]

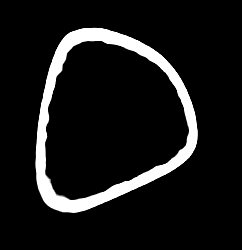

Supplement: S1 File — Individuals in central chimpanzee (Pan troglodytes troglodytes) and western lowland gorilla (Gorilla gorilla gorilla) are housed in the Primate Collection of the Department of Comparative Anatomy of the National Museum of Natural History, Paris, France (S1 Text). Humans (Homo sapiens) are housed in the Raymond A. Dart Collection of Human Skeletons at the University of the Witwatersrand, Johannesburg, South Africa (S1 Text) [70]. (ZIP) [file pone.0117905.s004.zip › X-Ray_Cortical structure of hallucal metatarsals and locomotor adaptations in hominoids/26_M_A17_Soto_MT1L.0001.bmp]

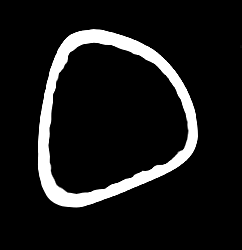

Supplement: S1 File — Individuals in central chimpanzee (Pan troglodytes troglodytes) and western lowland gorilla (Gorilla gorilla gorilla) are housed in the Primate Collection of the Department of Comparative Anatomy of the National Museum of Natural History, Paris, France (S1 Text). Humans (Homo sapiens) are housed in the Raymond A. Dart Collection of Human Skeletons at the University of the Witwatersrand, Johannesburg, South Africa (S1 Text) [70]. (ZIP) [file pone.0117905.s004.zip › X-Ray_Cortical structure of hallucal metatarsals and locomotor adaptations in hominoids/26_M_A17_Soto_MT1L.0002.bmp]

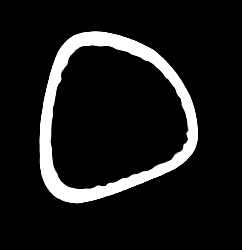

Supplement: S1 File — Individuals in central chimpanzee (Pan troglodytes troglodytes) and western lowland gorilla (Gorilla gorilla gorilla) are housed in the Primate Collection of the Department of Comparative Anatomy of the National Museum of Natural History, Paris, France (S1 Text). Humans (Homo sapiens) are housed in the Raymond A. Dart Collection of Human Skeletons at the University of the Witwatersrand, Johannesburg, South Africa (S1 Text) [70]. (ZIP) [file pone.0117905.s004.zip › X-Ray_Cortical structure of hallucal metatarsals and locomotor adaptations in hominoids/26_M_A17_Soto_MT1L.0003.bmp]

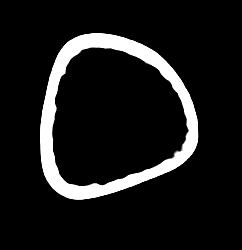

Supplement: S1 File — Individuals in central chimpanzee (Pan troglodytes troglodytes) and western lowland gorilla (Gorilla gorilla gorilla) are housed in the Primate Collection of the Department of Comparative Anatomy of the National Museum of Natural History, Paris, France (S1 Text). Humans (Homo sapiens) are housed in the Raymond A. Dart Collection of Human Skeletons at the University of the Witwatersrand, Johannesburg, South Africa (S1 Text) [70]. (ZIP) [file pone.0117905.s004.zip › X-Ray_Cortical structure of hallucal metatarsals and locomotor adaptations in hominoids/26_M_A17_Soto_MT1L.0004.bmp]

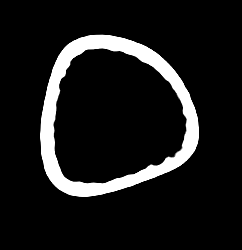

Supplement: S1 File — Individuals in central chimpanzee (Pan troglodytes troglodytes) and western lowland gorilla (Gorilla gorilla gorilla) are housed in the Primate Collection of the Department of Comparative Anatomy of the National Museum of Natural History, Paris, France (S1 Text). Humans (Homo sapiens) are housed in the Raymond A. Dart Collection of Human Skeletons at the University of the Witwatersrand, Johannesburg, South Africa (S1 Text) [70]. (ZIP) [file pone.0117905.s004.zip › X-Ray_Cortical structure of hallucal metatarsals and locomotor adaptations in hominoids/26_M_A17_Soto_MT1L.0005.bmp]

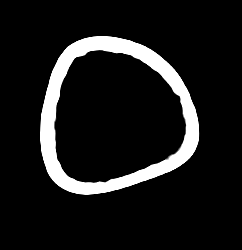

Supplement: S1 File — Individuals in central chimpanzee (Pan troglodytes troglodytes) and western lowland gorilla (Gorilla gorilla gorilla) are housed in the Primate Collection of the Department of Comparative Anatomy of the National Museum of Natural History, Paris, France (S1 Text). Humans (Homo sapiens) are housed in the Raymond A. Dart Collection of Human Skeletons at the University of the Witwatersrand, Johannesburg, South Africa (S1 Text) [70]. (ZIP) [file pone.0117905.s004.zip › X-Ray_Cortical structure of hallucal metatarsals and locomotor adaptations in hominoids/26_M_A17_Soto_MT1L.0006.bmp]

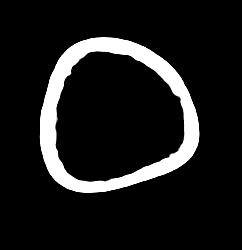

Supplement: S1 File — Individuals in central chimpanzee (Pan troglodytes troglodytes) and western lowland gorilla (Gorilla gorilla gorilla) are housed in the Primate Collection of the Department of Comparative Anatomy of the National Museum of Natural History, Paris, France (S1 Text). Humans (Homo sapiens) are housed in the Raymond A. Dart Collection of Human Skeletons at the University of the Witwatersrand, Johannesburg, South Africa (S1 Text) [70]. (ZIP) [file pone.0117905.s004.zip › X-Ray_Cortical structure of hallucal metatarsals and locomotor adaptations in hominoids/26_M_A17_Soto_MT1L.0007.bmp]

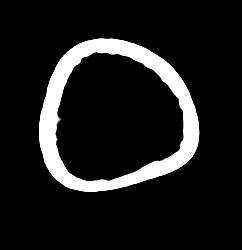

Supplement: S1 File — Individuals in central chimpanzee (Pan troglodytes troglodytes) and western lowland gorilla (Gorilla gorilla gorilla) are housed in the Primate Collection of the Department of Comparative Anatomy of the National Museum of Natural History, Paris, France (S1 Text). Humans (Homo sapiens) are housed in the Raymond A. Dart Collection of Human Skeletons at the University of the Witwatersrand, Johannesburg, South Africa (S1 Text) [70]. (ZIP) [file pone.0117905.s004.zip › X-Ray_Cortical structure of hallucal metatarsals and locomotor adaptations in hominoids/26_M_A17_Soto_MT1L.0008.bmp]

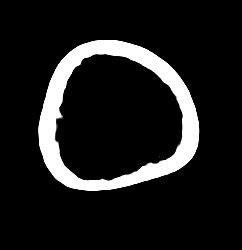

Supplement: S1 File — Individuals in central chimpanzee (Pan troglodytes troglodytes) and western lowland gorilla (Gorilla gorilla gorilla) are housed in the Primate Collection of the Department of Comparative Anatomy of the National Museum of Natural History, Paris, France (S1 Text). Humans (Homo sapiens) are housed in the Raymond A. Dart Collection of Human Skeletons at the University of the Witwatersrand, Johannesburg, South Africa (S1 Text) [70]. (ZIP) [file pone.0117905.s004.zip › X-Ray_Cortical structure of hallucal metatarsals and locomotor adaptations in hominoids/26_M_A17_Soto_MT1L.0009.bmp]

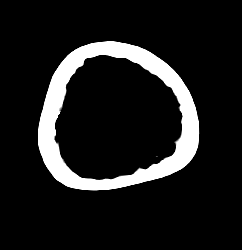

Supplement: S1 File — Individuals in central chimpanzee (Pan troglodytes troglodytes) and western lowland gorilla (Gorilla gorilla gorilla) are housed in the Primate Collection of the Department of Comparative Anatomy of the National Museum of Natural History, Paris, France (S1 Text). Humans (Homo sapiens) are housed in the Raymond A. Dart Collection of Human Skeletons at the University of the Witwatersrand, Johannesburg, South Africa (S1 Text) [70]. (ZIP) [file pone.0117905.s004.zip › X-Ray_Cortical structure of hallucal metatarsals and locomotor adaptations in hominoids/26_M_A17_Soto_MT1L.0010.bmp]

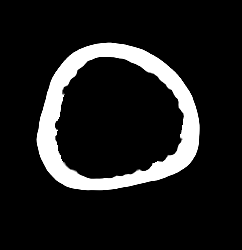

Supplement: S1 File — Individuals in central chimpanzee (Pan troglodytes troglodytes) and western lowland gorilla (Gorilla gorilla gorilla) are housed in the Primate Collection of the Department of Comparative Anatomy of the National Museum of Natural History, Paris, France (S1 Text). Humans (Homo sapiens) are housed in the Raymond A. Dart Collection of Human Skeletons at the University of the Witwatersrand, Johannesburg, South Africa (S1 Text) [70]. (ZIP) [file pone.0117905.s004.zip › X-Ray_Cortical structure of hallucal metatarsals and locomotor adaptations in hominoids/26_M_A17_Soto_MT1L.0011.bmp]

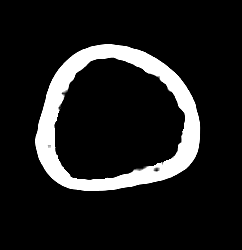

Supplement: S1 File — Individuals in central chimpanzee (Pan troglodytes troglodytes) and western lowland gorilla (Gorilla gorilla gorilla) are housed in the Primate Collection of the Department of Comparative Anatomy of the National Museum of Natural History, Paris, France (S1 Text). Humans (Homo sapiens) are housed in the Raymond A. Dart Collection of Human Skeletons at the University of the Witwatersrand, Johannesburg, South Africa (S1 Text) [70]. (ZIP) [file pone.0117905.s004.zip › X-Ray_Cortical structure of hallucal metatarsals and locomotor adaptations in hominoids/26_M_A17_Soto_MT1L.0012.bmp]

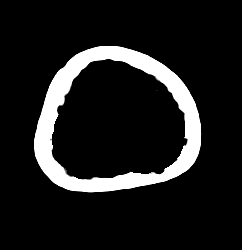

Supplement: S1 File — Individuals in central chimpanzee (Pan troglodytes troglodytes) and western lowland gorilla (Gorilla gorilla gorilla) are housed in the Primate Collection of the Department of Comparative Anatomy of the National Museum of Natural History, Paris, France (S1 Text). Humans (Homo sapiens) are housed in the Raymond A. Dart Collection of Human Skeletons at the University of the Witwatersrand, Johannesburg, South Africa (S1 Text) [70]. (ZIP) [file pone.0117905.s004.zip › X-Ray_Cortical structure of hallucal metatarsals and locomotor adaptations in hominoids/26_M_A17_Soto_MT1L.0013.bmp]

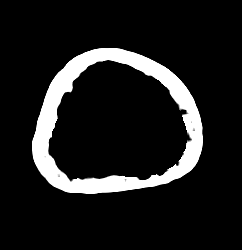

Supplement: S1 File — Individuals in central chimpanzee (Pan troglodytes troglodytes) and western lowland gorilla (Gorilla gorilla gorilla) are housed in the Primate Collection of the Department of Comparative Anatomy of the National Museum of Natural History, Paris, France (S1 Text). Humans (Homo sapiens) are housed in the Raymond A. Dart Collection of Human Skeletons at the University of the Witwatersrand, Johannesburg, South Africa (S1 Text) [70]. (ZIP) [file pone.0117905.s004.zip › X-Ray_Cortical structure of hallucal metatarsals and locomotor adaptations in hominoids/26_M_A17_Soto_MT1L.0014.bmp]

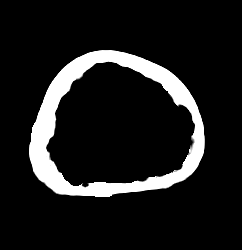

Supplement: S1 File — Individuals in central chimpanzee (Pan troglodytes troglodytes) and western lowland gorilla (Gorilla gorilla gorilla) are housed in the Primate Collection of the Department of Comparative Anatomy of the National Museum of Natural History, Paris, France (S1 Text). Humans (Homo sapiens) are housed in the Raymond A. Dart Collection of Human Skeletons at the University of the Witwatersrand, Johannesburg, South Africa (S1 Text) [70]. (ZIP) [file pone.0117905.s004.zip › X-Ray_Cortical structure of hallucal metatarsals and locomotor adaptations in hominoids/26_M_A17_Soto_MT1L.0015.bmp]

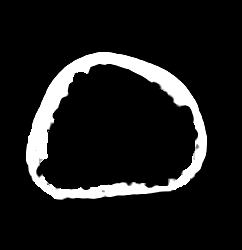

Supplement: S1 File — Individuals in central chimpanzee (Pan troglodytes troglodytes) and western lowland gorilla (Gorilla gorilla gorilla) are housed in the Primate Collection of the Department of Comparative Anatomy of the National Museum of Natural History, Paris, France (S1 Text). Humans (Homo sapiens) are housed in the Raymond A. Dart Collection of Human Skeletons at the University of the Witwatersrand, Johannesburg, South Africa (S1 Text) [70]. (ZIP) [file pone.0117905.s004.zip › X-Ray_Cortical structure of hallucal metatarsals and locomotor adaptations in hominoids/26_M_A17_Soto_MT1L.0016.bmp]

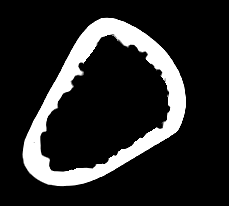

Supplement: S1 File — Individuals in central chimpanzee (Pan troglodytes troglodytes) and western lowland gorilla (Gorilla gorilla gorilla) are housed in the Primate Collection of the Department of Comparative Anatomy of the National Museum of Natural History, Paris, France (S1 Text). Humans (Homo sapiens) are housed in the Raymond A. Dart Collection of Human Skeletons at the University of the Witwatersrand, Johannesburg, South Africa (S1 Text) [70]. (ZIP) [file pone.0117905.s004.zip › X-Ray_Cortical structure of hallucal metatarsals and locomotor adaptations in hominoids/27_F_A883_Soto_MT1L.0000.bmp]

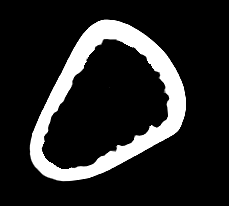

Supplement: S1 File — Individuals in central chimpanzee (Pan troglodytes troglodytes) and western lowland gorilla (Gorilla gorilla gorilla) are housed in the Primate Collection of the Department of Comparative Anatomy of the National Museum of Natural History, Paris, France (S1 Text). Humans (Homo sapiens) are housed in the Raymond A. Dart Collection of Human Skeletons at the University of the Witwatersrand, Johannesburg, South Africa (S1 Text) [70]. (ZIP) [file pone.0117905.s004.zip › X-Ray_Cortical structure of hallucal metatarsals and locomotor adaptations in hominoids/27_F_A883_Soto_MT1L.0001.bmp]

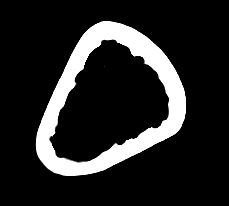

Supplement: S1 File — Individuals in central chimpanzee (Pan troglodytes troglodytes) and western lowland gorilla (Gorilla gorilla gorilla) are housed in the Primate Collection of the Department of Comparative Anatomy of the National Museum of Natural History, Paris, France (S1 Text). Humans (Homo sapiens) are housed in the Raymond A. Dart Collection of Human Skeletons at the University of the Witwatersrand, Johannesburg, South Africa (S1 Text) [70]. (ZIP) [file pone.0117905.s004.zip › X-Ray_Cortical structure of hallucal metatarsals and locomotor adaptations in hominoids/27_F_A883_Soto_MT1L.0002.bmp]

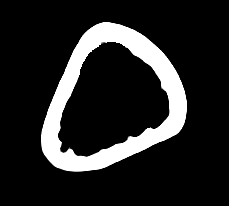

Supplement: S1 File — Individuals in central chimpanzee (Pan troglodytes troglodytes) and western lowland gorilla (Gorilla gorilla gorilla) are housed in the Primate Collection of the Department of Comparative Anatomy of the National Museum of Natural History, Paris, France (S1 Text). Humans (Homo sapiens) are housed in the Raymond A. Dart Collection of Human Skeletons at the University of the Witwatersrand, Johannesburg, South Africa (S1 Text) [70]. (ZIP) [file pone.0117905.s004.zip › X-Ray_Cortical structure of hallucal metatarsals and locomotor adaptations in hominoids/27_F_A883_Soto_MT1L.0003.bmp]

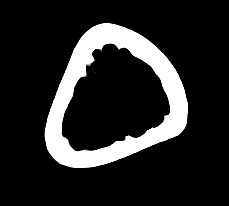

Supplement: S1 File — Individuals in central chimpanzee (Pan troglodytes troglodytes) and western lowland gorilla (Gorilla gorilla gorilla) are housed in the Primate Collection of the Department of Comparative Anatomy of the National Museum of Natural History, Paris, France (S1 Text). Humans (Homo sapiens) are housed in the Raymond A. Dart Collection of Human Skeletons at the University of the Witwatersrand, Johannesburg, South Africa (S1 Text) [70]. (ZIP) [file pone.0117905.s004.zip › X-Ray_Cortical structure of hallucal metatarsals and locomotor adaptations in hominoids/27_F_A883_Soto_MT1L.0004.bmp]

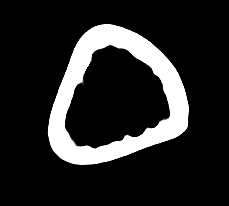

Supplement: S1 File — Individuals in central chimpanzee (Pan troglodytes troglodytes) and western lowland gorilla (Gorilla gorilla gorilla) are housed in the Primate Collection of the Department of Comparative Anatomy of the National Museum of Natural History, Paris, France (S1 Text). Humans (Homo sapiens) are housed in the Raymond A. Dart Collection of Human Skeletons at the University of the Witwatersrand, Johannesburg, South Africa (S1 Text) [70]. (ZIP) [file pone.0117905.s004.zip › X-Ray_Cortical structure of hallucal metatarsals and locomotor adaptations in hominoids/27_F_A883_Soto_MT1L.0005.bmp]

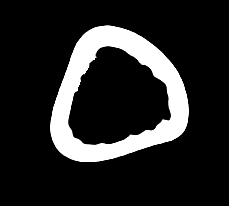

Supplement: S1 File — Individuals in central chimpanzee (Pan troglodytes troglodytes) and western lowland gorilla (Gorilla gorilla gorilla) are housed in the Primate Collection of the Department of Comparative Anatomy of the National Museum of Natural History, Paris, France (S1 Text). Humans (Homo sapiens) are housed in the Raymond A. Dart Collection of Human Skeletons at the University of the Witwatersrand, Johannesburg, South Africa (S1 Text) [70]. (ZIP) [file pone.0117905.s004.zip › X-Ray_Cortical structure of hallucal metatarsals and locomotor adaptations in hominoids/27_F_A883_Soto_MT1L.0006.bmp]

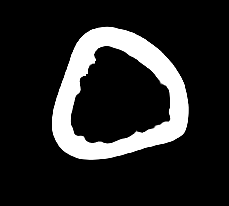

Supplement: S1 File — Individuals in central chimpanzee (Pan troglodytes troglodytes) and western lowland gorilla (Gorilla gorilla gorilla) are housed in the Primate Collection of the Department of Comparative Anatomy of the National Museum of Natural History, Paris, France (S1 Text). Humans (Homo sapiens) are housed in the Raymond A. Dart Collection of Human Skeletons at the University of the Witwatersrand, Johannesburg, South Africa (S1 Text) [70]. (ZIP) [file pone.0117905.s004.zip › X-Ray_Cortical structure of hallucal metatarsals and locomotor adaptations in hominoids/27_F_A883_Soto_MT1L.0007.bmp]

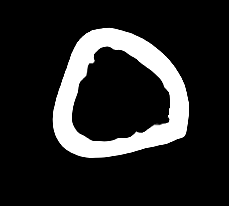

Supplement: S1 File — Individuals in central chimpanzee (Pan troglodytes troglodytes) and western lowland gorilla (Gorilla gorilla gorilla) are housed in the Primate Collection of the Department of Comparative Anatomy of the National Museum of Natural History, Paris, France (S1 Text). Humans (Homo sapiens) are housed in the Raymond A. Dart Collection of Human Skeletons at the University of the Witwatersrand, Johannesburg, South Africa (S1 Text) [70]. (ZIP) [file pone.0117905.s004.zip › X-Ray_Cortical structure of hallucal metatarsals and locomotor adaptations in hominoids/27_F_A883_Soto_MT1L.0008.bmp]

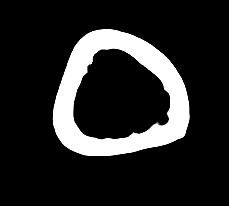

Supplement: S1 File — Individuals in central chimpanzee (Pan troglodytes troglodytes) and western lowland gorilla (Gorilla gorilla gorilla) are housed in the Primate Collection of the Department of Comparative Anatomy of the National Museum of Natural History, Paris, France (S1 Text). Humans (Homo sapiens) are housed in the Raymond A. Dart Collection of Human Skeletons at the University of the Witwatersrand, Johannesburg, South Africa (S1 Text) [70]. (ZIP) [file pone.0117905.s004.zip › X-Ray_Cortical structure of hallucal metatarsals and locomotor adaptations in hominoids/27_F_A883_Soto_MT1L.0009.bmp]

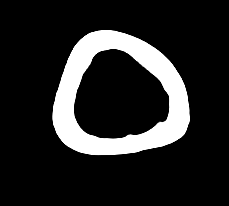

Supplement: S1 File — Individuals in central chimpanzee (Pan troglodytes troglodytes) and western lowland gorilla (Gorilla gorilla gorilla) are housed in the Primate Collection of the Department of Comparative Anatomy of the National Museum of Natural History, Paris, France (S1 Text). Humans (Homo sapiens) are housed in the Raymond A. Dart Collection of Human Skeletons at the University of the Witwatersrand, Johannesburg, South Africa (S1 Text) [70]. (ZIP) [file pone.0117905.s004.zip › X-Ray_Cortical structure of hallucal metatarsals and locomotor adaptations in hominoids/27_F_A883_Soto_MT1L.0010.bmp]

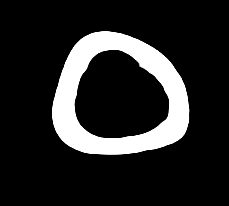

Supplement: S1 File — Individuals in central chimpanzee (Pan troglodytes troglodytes) and western lowland gorilla (Gorilla gorilla gorilla) are housed in the Primate Collection of the Department of Comparative Anatomy of the National Museum of Natural History, Paris, France (S1 Text). Humans (Homo sapiens) are housed in the Raymond A. Dart Collection of Human Skeletons at the University of the Witwatersrand, Johannesburg, South Africa (S1 Text) [70]. (ZIP) [file pone.0117905.s004.zip › X-Ray_Cortical structure of hallucal metatarsals and locomotor adaptations in hominoids/27_F_A883_Soto_MT1L.0011.bmp]

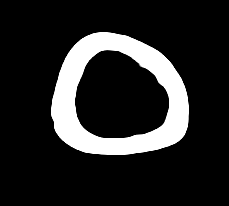

Supplement: S1 File — Individuals in central chimpanzee (Pan troglodytes troglodytes) and western lowland gorilla (Gorilla gorilla gorilla) are housed in the Primate Collection of the Department of Comparative Anatomy of the National Museum of Natural History, Paris, France (S1 Text). Humans (Homo sapiens) are housed in the Raymond A. Dart Collection of Human Skeletons at the University of the Witwatersrand, Johannesburg, South Africa (S1 Text) [70]. (ZIP) [file pone.0117905.s004.zip › X-Ray_Cortical structure of hallucal metatarsals and locomotor adaptations in hominoids/27_F_A883_Soto_MT1L.0012.bmp]

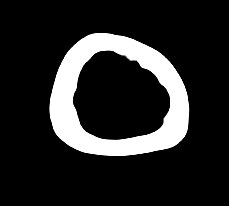

Supplement: S1 File — Individuals in central chimpanzee (Pan troglodytes troglodytes) and western lowland gorilla (Gorilla gorilla gorilla) are housed in the Primate Collection of the Department of Comparative Anatomy of the National Museum of Natural History, Paris, France (S1 Text). Humans (Homo sapiens) are housed in the Raymond A. Dart Collection of Human Skeletons at the University of the Witwatersrand, Johannesburg, South Africa (S1 Text) [70]. (ZIP) [file pone.0117905.s004.zip › X-Ray_Cortical structure of hallucal metatarsals and locomotor adaptations in hominoids/27_F_A883_Soto_MT1L.0013.bmp]

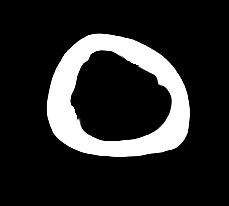

Supplement: S1 File — Individuals in central chimpanzee (Pan troglodytes troglodytes) and western lowland gorilla (Gorilla gorilla gorilla) are housed in the Primate Collection of the Department of Comparative Anatomy of the National Museum of Natural History, Paris, France (S1 Text). Humans (Homo sapiens) are housed in the Raymond A. Dart Collection of Human Skeletons at the University of the Witwatersrand, Johannesburg, South Africa (S1 Text) [70]. (ZIP) [file pone.0117905.s004.zip › X-Ray_Cortical structure of hallucal metatarsals and locomotor adaptations in hominoids/27_F_A883_Soto_MT1L.0014.bmp]

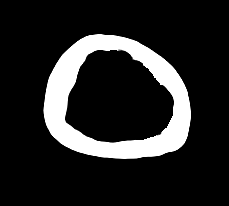

Supplement: S1 File — Individuals in central chimpanzee (Pan troglodytes troglodytes) and western lowland gorilla (Gorilla gorilla gorilla) are housed in the Primate Collection of the Department of Comparative Anatomy of the National Museum of Natural History, Paris, France (S1 Text). Humans (Homo sapiens) are housed in the Raymond A. Dart Collection of Human Skeletons at the University of the Witwatersrand, Johannesburg, South Africa (S1 Text) [70]. (ZIP) [file pone.0117905.s004.zip › X-Ray_Cortical structure of hallucal metatarsals and locomotor adaptations in hominoids/27_F_A883_Soto_MT1L.0015.bmp]

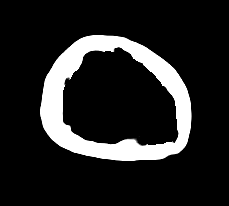

Supplement: S1 File — Individuals in central chimpanzee (Pan troglodytes troglodytes) and western lowland gorilla (Gorilla gorilla gorilla) are housed in the Primate Collection of the Department of Comparative Anatomy of the National Museum of Natural History, Paris, France (S1 Text). Humans (Homo sapiens) are housed in the Raymond A. Dart Collection of Human Skeletons at the University of the Witwatersrand, Johannesburg, South Africa (S1 Text) [70]. (ZIP) [file pone.0117905.s004.zip › X-Ray_Cortical structure of hallucal metatarsals and locomotor adaptations in hominoids/27_F_A883_Soto_MT1L.0016.bmp]

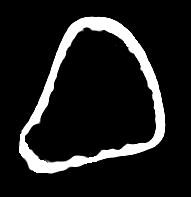

Supplement: S1 File — Individuals in central chimpanzee (Pan troglodytes troglodytes) and western lowland gorilla (Gorilla gorilla gorilla) are housed in the Primate Collection of the Department of Comparative Anatomy of the National Museum of Natural History, Paris, France (S1 Text). Humans (Homo sapiens) are housed in the Raymond A. Dart Collection of Human Skeletons at the University of the Witwatersrand, Johannesburg, South Africa (S1 Text) [70]. (ZIP) [file pone.0117905.s004.zip › X-Ray_Cortical structure of hallucal metatarsals and locomotor adaptations in hominoids/29_F_A1285_Soto_MT1.0000.bmp]

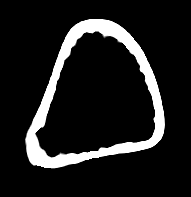

Supplement: S1 File — Individuals in central chimpanzee (Pan troglodytes troglodytes) and western lowland gorilla (Gorilla gorilla gorilla) are housed in the Primate Collection of the Department of Comparative Anatomy of the National Museum of Natural History, Paris, France (S1 Text). Humans (Homo sapiens) are housed in the Raymond A. Dart Collection of Human Skeletons at the University of the Witwatersrand, Johannesburg, South Africa (S1 Text) [70]. (ZIP) [file pone.0117905.s004.zip › X-Ray_Cortical structure of hallucal metatarsals and locomotor adaptations in hominoids/29_F_A1285_Soto_MT1.0001.bmp]

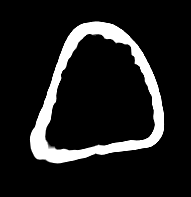

Supplement: S1 File — Individuals in central chimpanzee (Pan troglodytes troglodytes) and western lowland gorilla (Gorilla gorilla gorilla) are housed in the Primate Collection of the Department of Comparative Anatomy of the National Museum of Natural History, Paris, France (S1 Text). Humans (Homo sapiens) are housed in the Raymond A. Dart Collection of Human Skeletons at the University of the Witwatersrand, Johannesburg, South Africa (S1 Text) [70]. (ZIP) [file pone.0117905.s004.zip › X-Ray_Cortical structure of hallucal metatarsals and locomotor adaptations in hominoids/29_F_A1285_Soto_MT1.0002.bmp]

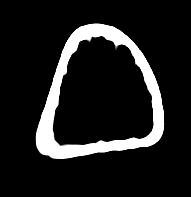

Supplement: S1 File — Individuals in central chimpanzee (Pan troglodytes troglodytes) and western lowland gorilla (Gorilla gorilla gorilla) are housed in the Primate Collection of the Department of Comparative Anatomy of the National Museum of Natural History, Paris, France (S1 Text). Humans (Homo sapiens) are housed in the Raymond A. Dart Collection of Human Skeletons at the University of the Witwatersrand, Johannesburg, South Africa (S1 Text) [70]. (ZIP) [file pone.0117905.s004.zip › X-Ray_Cortical structure of hallucal metatarsals and locomotor adaptations in hominoids/29_F_A1285_Soto_MT1.0003.bmp]

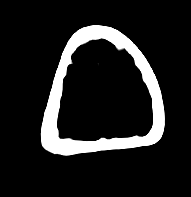

Supplement: S1 File — Individuals in central chimpanzee (Pan troglodytes troglodytes) and western lowland gorilla (Gorilla gorilla gorilla) are housed in the Primate Collection of the Department of Comparative Anatomy of the National Museum of Natural History, Paris, France (S1 Text). Humans (Homo sapiens) are housed in the Raymond A. Dart Collection of Human Skeletons at the University of the Witwatersrand, Johannesburg, South Africa (S1 Text) [70]. (ZIP) [file pone.0117905.s004.zip › X-Ray_Cortical structure of hallucal metatarsals and locomotor adaptations in hominoids/29_F_A1285_Soto_MT1.0004.bmp]

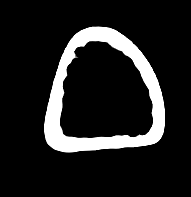

Supplement: S1 File — Individuals in central chimpanzee (Pan troglodytes troglodytes) and western lowland gorilla (Gorilla gorilla gorilla) are housed in the Primate Collection of the Department of Comparative Anatomy of the National Museum of Natural History, Paris, France (S1 Text). Humans (Homo sapiens) are housed in the Raymond A. Dart Collection of Human Skeletons at the University of the Witwatersrand, Johannesburg, South Africa (S1 Text) [70]. (ZIP) [file pone.0117905.s004.zip › X-Ray_Cortical structure of hallucal metatarsals and locomotor adaptations in hominoids/29_F_A1285_Soto_MT1.0005.bmp]

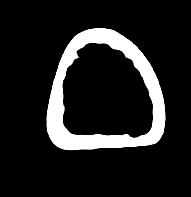

Supplement: S1 File — Individuals in central chimpanzee (Pan troglodytes troglodytes) and western lowland gorilla (Gorilla gorilla gorilla) are housed in the Primate Collection of the Department of Comparative Anatomy of the National Museum of Natural History, Paris, France (S1 Text). Humans (Homo sapiens) are housed in the Raymond A. Dart Collection of Human Skeletons at the University of the Witwatersrand, Johannesburg, South Africa (S1 Text) [70]. (ZIP) [file pone.0117905.s004.zip › X-Ray_Cortical structure of hallucal metatarsals and locomotor adaptations in hominoids/29_F_A1285_Soto_MT1.0006.bmp]

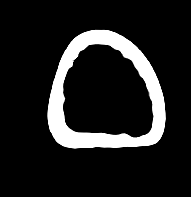

Supplement: S1 File — Individuals in central chimpanzee (Pan troglodytes troglodytes) and western lowland gorilla (Gorilla gorilla gorilla) are housed in the Primate Collection of the Department of Comparative Anatomy of the National Museum of Natural History, Paris, France (S1 Text). Humans (Homo sapiens) are housed in the Raymond A. Dart Collection of Human Skeletons at the University of the Witwatersrand, Johannesburg, South Africa (S1 Text) [70]. (ZIP) [file pone.0117905.s004.zip › X-Ray_Cortical structure of hallucal metatarsals and locomotor adaptations in hominoids/29_F_A1285_Soto_MT1.0007.bmp]

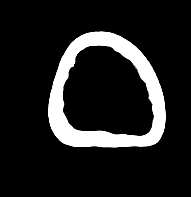

Supplement: S1 File — Individuals in central chimpanzee (Pan troglodytes troglodytes) and western lowland gorilla (Gorilla gorilla gorilla) are housed in the Primate Collection of the Department of Comparative Anatomy of the National Museum of Natural History, Paris, France (S1 Text). Humans (Homo sapiens) are housed in the Raymond A. Dart Collection of Human Skeletons at the University of the Witwatersrand, Johannesburg, South Africa (S1 Text) [70]. (ZIP) [file pone.0117905.s004.zip › X-Ray_Cortical structure of hallucal metatarsals and locomotor adaptations in hominoids/29_F_A1285_Soto_MT1.0008.bmp]

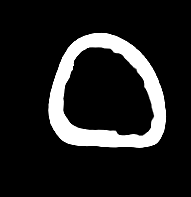

Supplement: S1 File — Individuals in central chimpanzee (Pan troglodytes troglodytes) and western lowland gorilla (Gorilla gorilla gorilla) are housed in the Primate Collection of the Department of Comparative Anatomy of the National Museum of Natural History, Paris, France (S1 Text). Humans (Homo sapiens) are housed in the Raymond A. Dart Collection of Human Skeletons at the University of the Witwatersrand, Johannesburg, South Africa (S1 Text) [70]. (ZIP) [file pone.0117905.s004.zip › X-Ray_Cortical structure of hallucal metatarsals and locomotor adaptations in hominoids/29_F_A1285_Soto_MT1.0009.bmp]

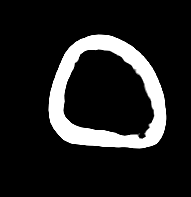

Supplement: S1 File — Individuals in central chimpanzee (Pan troglodytes troglodytes) and western lowland gorilla (Gorilla gorilla gorilla) are housed in the Primate Collection of the Department of Comparative Anatomy of the National Museum of Natural History, Paris, France (S1 Text). Humans (Homo sapiens) are housed in the Raymond A. Dart Collection of Human Skeletons at the University of the Witwatersrand, Johannesburg, South Africa (S1 Text) [70]. (ZIP) [file pone.0117905.s004.zip › X-Ray_Cortical structure of hallucal metatarsals and locomotor adaptations in hominoids/29_F_A1285_Soto_MT1.0010.bmp]

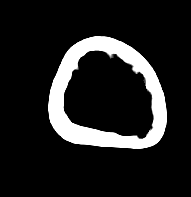

Supplement: S1 File — Individuals in central chimpanzee (Pan troglodytes troglodytes) and western lowland gorilla (Gorilla gorilla gorilla) are housed in the Primate Collection of the Department of Comparative Anatomy of the National Museum of Natural History, Paris, France (S1 Text). Humans (Homo sapiens) are housed in the Raymond A. Dart Collection of Human Skeletons at the University of the Witwatersrand, Johannesburg, South Africa (S1 Text) [70]. (ZIP) [file pone.0117905.s004.zip › X-Ray_Cortical structure of hallucal metatarsals and locomotor adaptations in hominoids/29_F_A1285_Soto_MT1.0011.bmp]

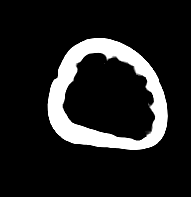

Supplement: S1 File — Individuals in central chimpanzee (Pan troglodytes troglodytes) and western lowland gorilla (Gorilla gorilla gorilla) are housed in the Primate Collection of the Department of Comparative Anatomy of the National Museum of Natural History, Paris, France (S1 Text). Humans (Homo sapiens) are housed in the Raymond A. Dart Collection of Human Skeletons at the University of the Witwatersrand, Johannesburg, South Africa (S1 Text) [70]. (ZIP) [file pone.0117905.s004.zip › X-Ray_Cortical structure of hallucal metatarsals and locomotor adaptations in hominoids/29_F_A1285_Soto_MT1.0012.bmp]

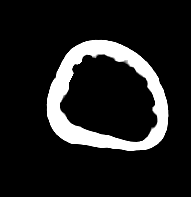

Supplement: S1 File — Individuals in central chimpanzee (Pan troglodytes troglodytes) and western lowland gorilla (Gorilla gorilla gorilla) are housed in the Primate Collection of the Department of Comparative Anatomy of the National Museum of Natural History, Paris, France (S1 Text). Humans (Homo sapiens) are housed in the Raymond A. Dart Collection of Human Skeletons at the University of the Witwatersrand, Johannesburg, South Africa (S1 Text) [70]. (ZIP) [file pone.0117905.s004.zip › X-Ray_Cortical structure of hallucal metatarsals and locomotor adaptations in hominoids/29_F_A1285_Soto_MT1.0013.bmp]

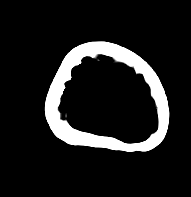

Supplement: S1 File — Individuals in central chimpanzee (Pan troglodytes troglodytes) and western lowland gorilla (Gorilla gorilla gorilla) are housed in the Primate Collection of the Department of Comparative Anatomy of the National Museum of Natural History, Paris, France (S1 Text). Humans (Homo sapiens) are housed in the Raymond A. Dart Collection of Human Skeletons at the University of the Witwatersrand, Johannesburg, South Africa (S1 Text) [70]. (ZIP) [file pone.0117905.s004.zip › X-Ray_Cortical structure of hallucal metatarsals and locomotor adaptations in hominoids/29_F_A1285_Soto_MT1.0014.bmp]

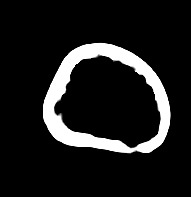

Supplement: S1 File — Individuals in central chimpanzee (Pan troglodytes troglodytes) and western lowland gorilla (Gorilla gorilla gorilla) are housed in the Primate Collection of the Department of Comparative Anatomy of the National Museum of Natural History, Paris, France (S1 Text). Humans (Homo sapiens) are housed in the Raymond A. Dart Collection of Human Skeletons at the University of the Witwatersrand, Johannesburg, South Africa (S1 Text) [70]. (ZIP) [file pone.0117905.s004.zip › X-Ray_Cortical structure of hallucal metatarsals and locomotor adaptations in hominoids/29_F_A1285_Soto_MT1.0015.bmp]

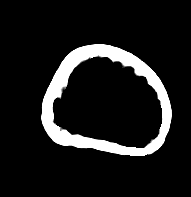

Supplement: S1 File — Individuals in central chimpanzee (Pan troglodytes troglodytes) and western lowland gorilla (Gorilla gorilla gorilla) are housed in the Primate Collection of the Department of Comparative Anatomy of the National Museum of Natural History, Paris, France (S1 Text). Humans (Homo sapiens) are housed in the Raymond A. Dart Collection of Human Skeletons at the University of the Witwatersrand, Johannesburg, South Africa (S1 Text) [70]. (ZIP) [file pone.0117905.s004.zip › X-Ray_Cortical structure of hallucal metatarsals and locomotor adaptations in hominoids/29_F_A1285_Soto_MT1.0016.bmp]

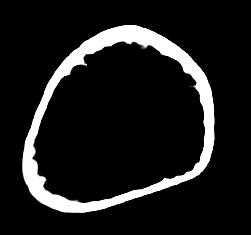

Supplement: S1 File — Individuals in central chimpanzee (Pan troglodytes troglodytes) and western lowland gorilla (Gorilla gorilla gorilla) are housed in the Primate Collection of the Department of Comparative Anatomy of the National Museum of Natural History, Paris, France (S1 Text). Humans (Homo sapiens) are housed in the Raymond A. Dart Collection of Human Skeletons at the University of the Witwatersrand, Johannesburg, South Africa (S1 Text) [70]. (ZIP) [file pone.0117905.s004.zip › X-Ray_Cortical structure of hallucal metatarsals and locomotor adaptations in hominoids/29_F_A3060_Soto_MT1L.0000.bmp]

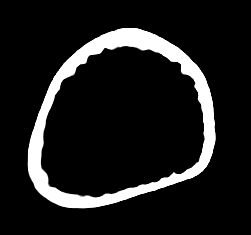

Supplement: S1 File — Individuals in central chimpanzee (Pan troglodytes troglodytes) and western lowland gorilla (Gorilla gorilla gorilla) are housed in the Primate Collection of the Department of Comparative Anatomy of the National Museum of Natural History, Paris, France (S1 Text). Humans (Homo sapiens) are housed in the Raymond A. Dart Collection of Human Skeletons at the University of the Witwatersrand, Johannesburg, South Africa (S1 Text) [70]. (ZIP) [file pone.0117905.s004.zip › X-Ray_Cortical structure of hallucal metatarsals and locomotor adaptations in hominoids/29_F_A3060_Soto_MT1L.0001.bmp]

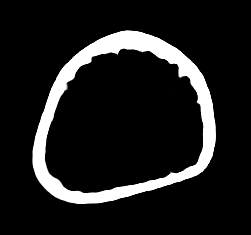

Supplement: S1 File — Individuals in central chimpanzee (Pan troglodytes troglodytes) and western lowland gorilla (Gorilla gorilla gorilla) are housed in the Primate Collection of the Department of Comparative Anatomy of the National Museum of Natural History, Paris, France (S1 Text). Humans (Homo sapiens) are housed in the Raymond A. Dart Collection of Human Skeletons at the University of the Witwatersrand, Johannesburg, South Africa (S1 Text) [70]. (ZIP) [file pone.0117905.s004.zip › X-Ray_Cortical structure of hallucal metatarsals and locomotor adaptations in hominoids/29_F_A3060_Soto_MT1L.0002.bmp]

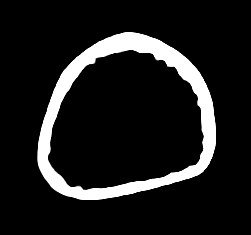

Supplement: S1 File — Individuals in central chimpanzee (Pan troglodytes troglodytes) and western lowland gorilla (Gorilla gorilla gorilla) are housed in the Primate Collection of the Department of Comparative Anatomy of the National Museum of Natural History, Paris, France (S1 Text). Humans (Homo sapiens) are housed in the Raymond A. Dart Collection of Human Skeletons at the University of the Witwatersrand, Johannesburg, South Africa (S1 Text) [70]. (ZIP) [file pone.0117905.s004.zip › X-Ray_Cortical structure of hallucal metatarsals and locomotor adaptations in hominoids/29_F_A3060_Soto_MT1L.0003.bmp]

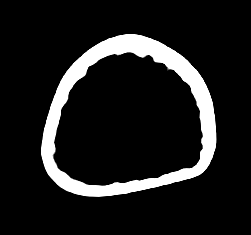

Supplement: S1 File — Individuals in central chimpanzee (Pan troglodytes troglodytes) and western lowland gorilla (Gorilla gorilla gorilla) are housed in the Primate Collection of the Department of Comparative Anatomy of the National Museum of Natural History, Paris, France (S1 Text). Humans (Homo sapiens) are housed in the Raymond A. Dart Collection of Human Skeletons at the University of the Witwatersrand, Johannesburg, South Africa (S1 Text) [70]. (ZIP) [file pone.0117905.s004.zip › X-Ray_Cortical structure of hallucal metatarsals and locomotor adaptations in hominoids/29_F_A3060_Soto_MT1L.0004.bmp]

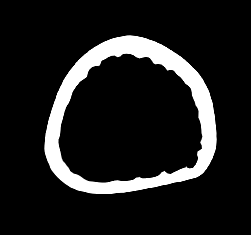

Supplement: S1 File — Individuals in central chimpanzee (Pan troglodytes troglodytes) and western lowland gorilla (Gorilla gorilla gorilla) are housed in the Primate Collection of the Department of Comparative Anatomy of the National Museum of Natural History, Paris, France (S1 Text). Humans (Homo sapiens) are housed in the Raymond A. Dart Collection of Human Skeletons at the University of the Witwatersrand, Johannesburg, South Africa (S1 Text) [70]. (ZIP) [file pone.0117905.s004.zip › X-Ray_Cortical structure of hallucal metatarsals and locomotor adaptations in hominoids/29_F_A3060_Soto_MT1L.0005.bmp]

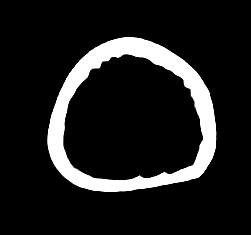

Supplement: S1 File — Individuals in central chimpanzee (Pan troglodytes troglodytes) and western lowland gorilla (Gorilla gorilla gorilla) are housed in the Primate Collection of the Department of Comparative Anatomy of the National Museum of Natural History, Paris, France (S1 Text). Humans (Homo sapiens) are housed in the Raymond A. Dart Collection of Human Skeletons at the University of the Witwatersrand, Johannesburg, South Africa (S1 Text) [70]. (ZIP) [file pone.0117905.s004.zip › X-Ray_Cortical structure of hallucal metatarsals and locomotor adaptations in hominoids/29_F_A3060_Soto_MT1L.0006.bmp]

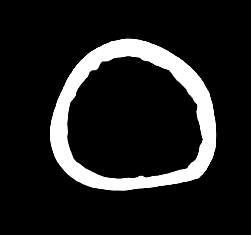

Supplement: S1 File — Individuals in central chimpanzee (Pan troglodytes troglodytes) and western lowland gorilla (Gorilla gorilla gorilla) are housed in the Primate Collection of the Department of Comparative Anatomy of the National Museum of Natural History, Paris, France (S1 Text). Humans (Homo sapiens) are housed in the Raymond A. Dart Collection of Human Skeletons at the University of the Witwatersrand, Johannesburg, South Africa (S1 Text) [70]. (ZIP) [file pone.0117905.s004.zip › X-Ray_Cortical structure of hallucal metatarsals and locomotor adaptations in hominoids/29_F_A3060_Soto_MT1L.0007.bmp]

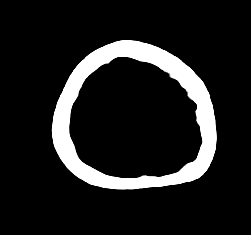

Supplement: S1 File — Individuals in central chimpanzee (Pan troglodytes troglodytes) and western lowland gorilla (Gorilla gorilla gorilla) are housed in the Primate Collection of the Department of Comparative Anatomy of the National Museum of Natural History, Paris, France (S1 Text). Humans (Homo sapiens) are housed in the Raymond A. Dart Collection of Human Skeletons at the University of the Witwatersrand, Johannesburg, South Africa (S1 Text) [70]. (ZIP) [file pone.0117905.s004.zip › X-Ray_Cortical structure of hallucal metatarsals and locomotor adaptations in hominoids/29_F_A3060_Soto_MT1L.0008.bmp]

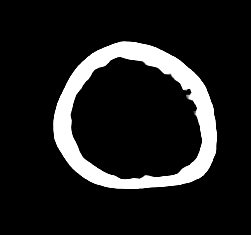

Supplement: S1 File — Individuals in central chimpanzee (Pan troglodytes troglodytes) and western lowland gorilla (Gorilla gorilla gorilla) are housed in the Primate Collection of the Department of Comparative Anatomy of the National Museum of Natural History, Paris, France (S1 Text). Humans (Homo sapiens) are housed in the Raymond A. Dart Collection of Human Skeletons at the University of the Witwatersrand, Johannesburg, South Africa (S1 Text) [70]. (ZIP) [file pone.0117905.s004.zip › X-Ray_Cortical structure of hallucal metatarsals and locomotor adaptations in hominoids/29_F_A3060_Soto_MT1L.0009.bmp]

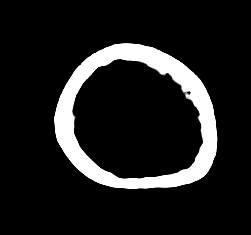

Supplement: S1 File — Individuals in central chimpanzee (Pan troglodytes troglodytes) and western lowland gorilla (Gorilla gorilla gorilla) are housed in the Primate Collection of the Department of Comparative Anatomy of the National Museum of Natural History, Paris, France (S1 Text). Humans (Homo sapiens) are housed in the Raymond A. Dart Collection of Human Skeletons at the University of the Witwatersrand, Johannesburg, South Africa (S1 Text) [70]. (ZIP) [file pone.0117905.s004.zip › X-Ray_Cortical structure of hallucal metatarsals and locomotor adaptations in hominoids/29_F_A3060_Soto_MT1L.0010.bmp]

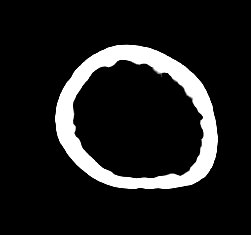

Supplement: S1 File — Individuals in central chimpanzee (Pan troglodytes troglodytes) and western lowland gorilla (Gorilla gorilla gorilla) are housed in the Primate Collection of the Department of Comparative Anatomy of the National Museum of Natural History, Paris, France (S1 Text). Humans (Homo sapiens) are housed in the Raymond A. Dart Collection of Human Skeletons at the University of the Witwatersrand, Johannesburg, South Africa (S1 Text) [70]. (ZIP) [file pone.0117905.s004.zip › X-Ray_Cortical structure of hallucal metatarsals and locomotor adaptations in hominoids/29_F_A3060_Soto_MT1L.0011.bmp]

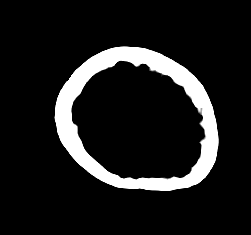

Supplement: S1 File — Individuals in central chimpanzee (Pan troglodytes troglodytes) and western lowland gorilla (Gorilla gorilla gorilla) are housed in the Primate Collection of the Department of Comparative Anatomy of the National Museum of Natural History, Paris, France (S1 Text). Humans (Homo sapiens) are housed in the Raymond A. Dart Collection of Human Skeletons at the University of the Witwatersrand, Johannesburg, South Africa (S1 Text) [70]. (ZIP) [file pone.0117905.s004.zip › X-Ray_Cortical structure of hallucal metatarsals and locomotor adaptations in hominoids/29_F_A3060_Soto_MT1L.0012.bmp]

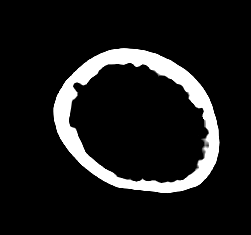

Supplement: S1 File — Individuals in central chimpanzee (Pan troglodytes troglodytes) and western lowland gorilla (Gorilla gorilla gorilla) are housed in the Primate Collection of the Department of Comparative Anatomy of the National Museum of Natural History, Paris, France (S1 Text). Humans (Homo sapiens) are housed in the Raymond A. Dart Collection of Human Skeletons at the University of the Witwatersrand, Johannesburg, South Africa (S1 Text) [70]. (ZIP) [file pone.0117905.s004.zip › X-Ray_Cortical structure of hallucal metatarsals and locomotor adaptations in hominoids/29_F_A3060_Soto_MT1L.0013.bmp]

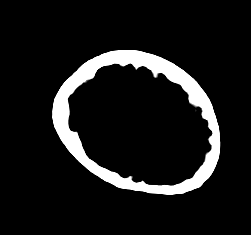

Supplement: S1 File — Individuals in central chimpanzee (Pan troglodytes troglodytes) and western lowland gorilla (Gorilla gorilla gorilla) are housed in the Primate Collection of the Department of Comparative Anatomy of the National Museum of Natural History, Paris, France (S1 Text). Humans (Homo sapiens) are housed in the Raymond A. Dart Collection of Human Skeletons at the University of the Witwatersrand, Johannesburg, South Africa (S1 Text) [70]. (ZIP) [file pone.0117905.s004.zip › X-Ray_Cortical structure of hallucal metatarsals and locomotor adaptations in hominoids/29_F_A3060_Soto_MT1L.0014.bmp]

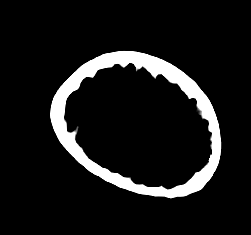

Supplement: S1 File — Individuals in central chimpanzee (Pan troglodytes troglodytes) and western lowland gorilla (Gorilla gorilla gorilla) are housed in the Primate Collection of the Department of Comparative Anatomy of the National Museum of Natural History, Paris, France (S1 Text). Humans (Homo sapiens) are housed in the Raymond A. Dart Collection of Human Skeletons at the University of the Witwatersrand, Johannesburg, South Africa (S1 Text) [70]. (ZIP) [file pone.0117905.s004.zip › X-Ray_Cortical structure of hallucal metatarsals and locomotor adaptations in hominoids/29_F_A3060_Soto_MT1L.0015.bmp]

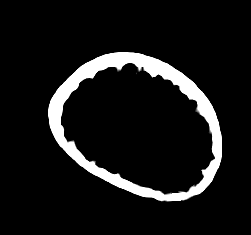

Supplement: S1 File — Individuals in central chimpanzee (Pan troglodytes troglodytes) and western lowland gorilla (Gorilla gorilla gorilla) are housed in the Primate Collection of the Department of Comparative Anatomy of the National Museum of Natural History, Paris, France (S1 Text). Humans (Homo sapiens) are housed in the Raymond A. Dart Collection of Human Skeletons at the University of the Witwatersrand, Johannesburg, South Africa (S1 Text) [70]. (ZIP) [file pone.0117905.s004.zip › X-Ray_Cortical structure of hallucal metatarsals and locomotor adaptations in hominoids/29_F_A3060_Soto_MT1L.0016.bmp]

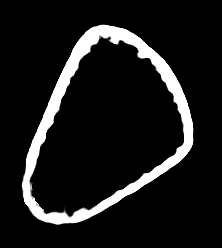

Supplement: S1 File — Individuals in central chimpanzee (Pan troglodytes troglodytes) and western lowland gorilla (Gorilla gorilla gorilla) are housed in the Primate Collection of the Department of Comparative Anatomy of the National Museum of Natural History, Paris, France (S1 Text). Humans (Homo sapiens) are housed in the Raymond A. Dart Collection of Human Skeletons at the University of the Witwatersrand, Johannesburg, South Africa (S1 Text) [70]. (ZIP) [file pone.0117905.s004.zip › X-Ray_Cortical structure of hallucal metatarsals and locomotor adaptations in hominoids/29_M_A170_Soto_MT1L.0000.bmp]

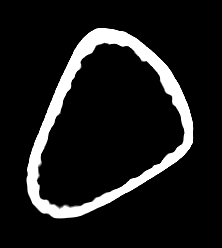

Supplement: S1 File — Individuals in central chimpanzee (Pan troglodytes troglodytes) and western lowland gorilla (Gorilla gorilla gorilla) are housed in the Primate Collection of the Department of Comparative Anatomy of the National Museum of Natural History, Paris, France (S1 Text). Humans (Homo sapiens) are housed in the Raymond A. Dart Collection of Human Skeletons at the University of the Witwatersrand, Johannesburg, South Africa (S1 Text) [70]. (ZIP) [file pone.0117905.s004.zip › X-Ray_Cortical structure of hallucal metatarsals and locomotor adaptations in hominoids/29_M_A170_Soto_MT1L.0001.bmp]

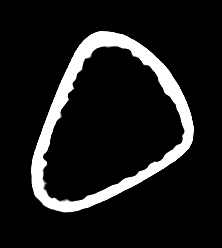

Supplement: S1 File — Individuals in central chimpanzee (Pan troglodytes troglodytes) and western lowland gorilla (Gorilla gorilla gorilla) are housed in the Primate Collection of the Department of Comparative Anatomy of the National Museum of Natural History, Paris, France (S1 Text). Humans (Homo sapiens) are housed in the Raymond A. Dart Collection of Human Skeletons at the University of the Witwatersrand, Johannesburg, South Africa (S1 Text) [70]. (ZIP) [file pone.0117905.s004.zip › X-Ray_Cortical structure of hallucal metatarsals and locomotor adaptations in hominoids/29_M_A170_Soto_MT1L.0002.bmp]

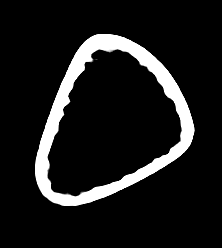

Supplement: S1 File — Individuals in central chimpanzee (Pan troglodytes troglodytes) and western lowland gorilla (Gorilla gorilla gorilla) are housed in the Primate Collection of the Department of Comparative Anatomy of the National Museum of Natural History, Paris, France (S1 Text). Humans (Homo sapiens) are housed in the Raymond A. Dart Collection of Human Skeletons at the University of the Witwatersrand, Johannesburg, South Africa (S1 Text) [70]. (ZIP) [file pone.0117905.s004.zip › X-Ray_Cortical structure of hallucal metatarsals and locomotor adaptations in hominoids/29_M_A170_Soto_MT1L.0003.bmp]

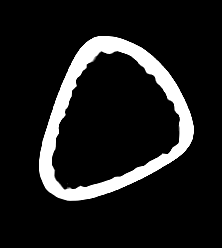

Supplement: S1 File — Individuals in central chimpanzee (Pan troglodytes troglodytes) and western lowland gorilla (Gorilla gorilla gorilla) are housed in the Primate Collection of the Department of Comparative Anatomy of the National Museum of Natural History, Paris, France (S1 Text). Humans (Homo sapiens) are housed in the Raymond A. Dart Collection of Human Skeletons at the University of the Witwatersrand, Johannesburg, South Africa (S1 Text) [70]. (ZIP) [file pone.0117905.s004.zip › X-Ray_Cortical structure of hallucal metatarsals and locomotor adaptations in hominoids/29_M_A170_Soto_MT1L.0004.bmp]

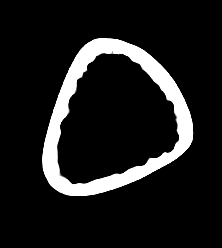

Supplement: S1 File — Individuals in central chimpanzee (Pan troglodytes troglodytes) and western lowland gorilla (Gorilla gorilla gorilla) are housed in the Primate Collection of the Department of Comparative Anatomy of the National Museum of Natural History, Paris, France (S1 Text). Humans (Homo sapiens) are housed in the Raymond A. Dart Collection of Human Skeletons at the University of the Witwatersrand, Johannesburg, South Africa (S1 Text) [70]. (ZIP) [file pone.0117905.s004.zip › X-Ray_Cortical structure of hallucal metatarsals and locomotor adaptations in hominoids/29_M_A170_Soto_MT1L.0005.bmp]

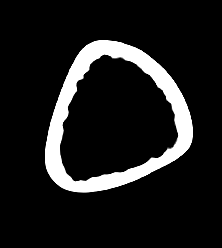

Supplement: S1 File — Individuals in central chimpanzee (Pan troglodytes troglodytes) and western lowland gorilla (Gorilla gorilla gorilla) are housed in the Primate Collection of the Department of Comparative Anatomy of the National Museum of Natural History, Paris, France (S1 Text). Humans (Homo sapiens) are housed in the Raymond A. Dart Collection of Human Skeletons at the University of the Witwatersrand, Johannesburg, South Africa (S1 Text) [70]. (ZIP) [file pone.0117905.s004.zip › X-Ray_Cortical structure of hallucal metatarsals and locomotor adaptations in hominoids/29_M_A170_Soto_MT1L.0006.bmp]

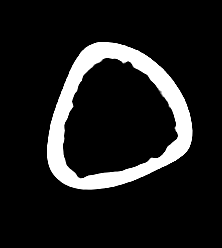

Supplement: S1 File — Individuals in central chimpanzee (Pan troglodytes troglodytes) and western lowland gorilla (Gorilla gorilla gorilla) are housed in the Primate Collection of the Department of Comparative Anatomy of the National Museum of Natural History, Paris, France (S1 Text). Humans (Homo sapiens) are housed in the Raymond A. Dart Collection of Human Skeletons at the University of the Witwatersrand, Johannesburg, South Africa (S1 Text) [70]. (ZIP) [file pone.0117905.s004.zip › X-Ray_Cortical structure of hallucal metatarsals and locomotor adaptations in hominoids/29_M_A170_Soto_MT1L.0007.bmp]

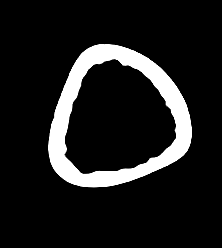

Supplement: S1 File — Individuals in central chimpanzee (Pan troglodytes troglodytes) and western lowland gorilla (Gorilla gorilla gorilla) are housed in the Primate Collection of the Department of Comparative Anatomy of the National Museum of Natural History, Paris, France (S1 Text). Humans (Homo sapiens) are housed in the Raymond A. Dart Collection of Human Skeletons at the University of the Witwatersrand, Johannesburg, South Africa (S1 Text) [70]. (ZIP) [file pone.0117905.s004.zip › X-Ray_Cortical structure of hallucal metatarsals and locomotor adaptations in hominoids/29_M_A170_Soto_MT1L.0008.bmp]

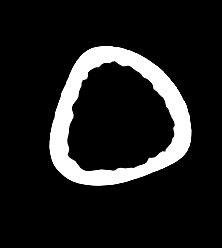

Supplement: S1 File — Individuals in central chimpanzee (Pan troglodytes troglodytes) and western lowland gorilla (Gorilla gorilla gorilla) are housed in the Primate Collection of the Department of Comparative Anatomy of the National Museum of Natural History, Paris, France (S1 Text). Humans (Homo sapiens) are housed in the Raymond A. Dart Collection of Human Skeletons at the University of the Witwatersrand, Johannesburg, South Africa (S1 Text) [70]. (ZIP) [file pone.0117905.s004.zip › X-Ray_Cortical structure of hallucal metatarsals and locomotor adaptations in hominoids/29_M_A170_Soto_MT1L.0009.bmp]

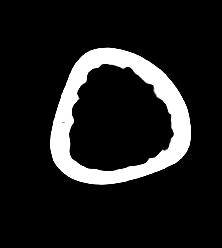

Supplement: S1 File — Individuals in central chimpanzee (Pan troglodytes troglodytes) and western lowland gorilla (Gorilla gorilla gorilla) are housed in the Primate Collection of the Department of Comparative Anatomy of the National Museum of Natural History, Paris, France (S1 Text). Humans (Homo sapiens) are housed in the Raymond A. Dart Collection of Human Skeletons at the University of the Witwatersrand, Johannesburg, South Africa (S1 Text) [70]. (ZIP) [file pone.0117905.s004.zip › X-Ray_Cortical structure of hallucal metatarsals and locomotor adaptations in hominoids/29_M_A170_Soto_MT1L.0010.bmp]

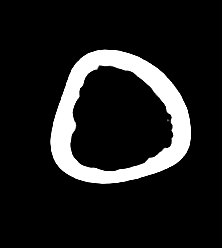

Supplement: S1 File — Individuals in central chimpanzee (Pan troglodytes troglodytes) and western lowland gorilla (Gorilla gorilla gorilla) are housed in the Primate Collection of the Department of Comparative Anatomy of the National Museum of Natural History, Paris, France (S1 Text). Humans (Homo sapiens) are housed in the Raymond A. Dart Collection of Human Skeletons at the University of the Witwatersrand, Johannesburg, South Africa (S1 Text) [70]. (ZIP) [file pone.0117905.s004.zip › X-Ray_Cortical structure of hallucal metatarsals and locomotor adaptations in hominoids/29_M_A170_Soto_MT1L.0011.bmp]

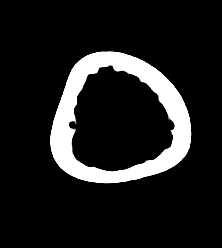

Supplement: S1 File — Individuals in central chimpanzee (Pan troglodytes troglodytes) and western lowland gorilla (Gorilla gorilla gorilla) are housed in the Primate Collection of the Department of Comparative Anatomy of the National Museum of Natural History, Paris, France (S1 Text). Humans (Homo sapiens) are housed in the Raymond A. Dart Collection of Human Skeletons at the University of the Witwatersrand, Johannesburg, South Africa (S1 Text) [70]. (ZIP) [file pone.0117905.s004.zip › X-Ray_Cortical structure of hallucal metatarsals and locomotor adaptations in hominoids/29_M_A170_Soto_MT1L.0012.bmp]

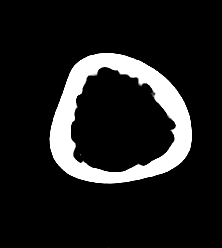

Supplement: S1 File — Individuals in central chimpanzee (Pan troglodytes troglodytes) and western lowland gorilla (Gorilla gorilla gorilla) are housed in the Primate Collection of the Department of Comparative Anatomy of the National Museum of Natural History, Paris, France (S1 Text). Humans (Homo sapiens) are housed in the Raymond A. Dart Collection of Human Skeletons at the University of the Witwatersrand, Johannesburg, South Africa (S1 Text) [70]. (ZIP) [file pone.0117905.s004.zip › X-Ray_Cortical structure of hallucal metatarsals and locomotor adaptations in hominoids/29_M_A170_Soto_MT1L.0013.bmp]

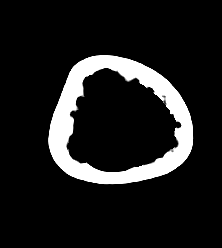

Supplement: S1 File — Individuals in central chimpanzee (Pan troglodytes troglodytes) and western lowland gorilla (Gorilla gorilla gorilla) are housed in the Primate Collection of the Department of Comparative Anatomy of the National Museum of Natural History, Paris, France (S1 Text). Humans (Homo sapiens) are housed in the Raymond A. Dart Collection of Human Skeletons at the University of the Witwatersrand, Johannesburg, South Africa (S1 Text) [70]. (ZIP) [file pone.0117905.s004.zip › X-Ray_Cortical structure of hallucal metatarsals and locomotor adaptations in hominoids/29_M_A170_Soto_MT1L.0014.bmp]

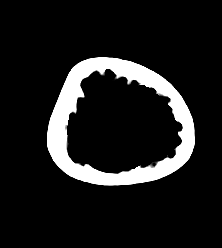

Supplement: S1 File — Individuals in central chimpanzee (Pan troglodytes troglodytes) and western lowland gorilla (Gorilla gorilla gorilla) are housed in the Primate Collection of the Department of Comparative Anatomy of the National Museum of Natural History, Paris, France (S1 Text). Humans (Homo sapiens) are housed in the Raymond A. Dart Collection of Human Skeletons at the University of the Witwatersrand, Johannesburg, South Africa (S1 Text) [70]. (ZIP) [file pone.0117905.s004.zip › X-Ray_Cortical structure of hallucal metatarsals and locomotor adaptations in hominoids/29_M_A170_Soto_MT1L.0015.bmp]

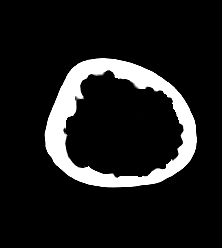

Supplement: S1 File — Individuals in central chimpanzee (Pan troglodytes troglodytes) and western lowland gorilla (Gorilla gorilla gorilla) are housed in the Primate Collection of the Department of Comparative Anatomy of the National Museum of Natural History, Paris, France (S1 Text). Humans (Homo sapiens) are housed in the Raymond A. Dart Collection of Human Skeletons at the University of the Witwatersrand, Johannesburg, South Africa (S1 Text) [70]. (ZIP) [file pone.0117905.s004.zip › X-Ray_Cortical structure of hallucal metatarsals and locomotor adaptations in hominoids/29_M_A170_Soto_MT1L.0016.bmp]

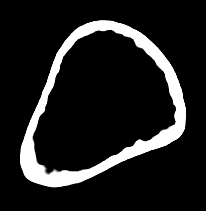

Supplement: S1 File — Individuals in central chimpanzee (Pan troglodytes troglodytes) and western lowland gorilla (Gorilla gorilla gorilla) are housed in the Primate Collection of the Department of Comparative Anatomy of the National Museum of Natural History, Paris, France (S1 Text). Humans (Homo sapiens) are housed in the Raymond A. Dart Collection of Human Skeletons at the University of the Witwatersrand, Johannesburg, South Africa (S1 Text) [70]. (ZIP) [file pone.0117905.s004.zip › X-Ray_Cortical structure of hallucal metatarsals and locomotor adaptations in hominoids/30_F_A22_Xosa_MT1L.0000.bmp]

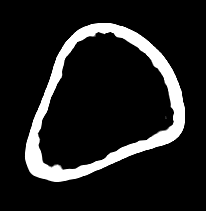

Supplement: S1 File — Individuals in central chimpanzee (Pan troglodytes troglodytes) and western lowland gorilla (Gorilla gorilla gorilla) are housed in the Primate Collection of the Department of Comparative Anatomy of the National Museum of Natural History, Paris, France (S1 Text). Humans (Homo sapiens) are housed in the Raymond A. Dart Collection of Human Skeletons at the University of the Witwatersrand, Johannesburg, South Africa (S1 Text) [70]. (ZIP) [file pone.0117905.s004.zip › X-Ray_Cortical structure of hallucal metatarsals and locomotor adaptations in hominoids/30_F_A22_Xosa_MT1L.0001.bmp]

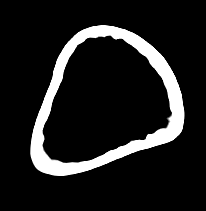

Supplement: S1 File — Individuals in central chimpanzee (Pan troglodytes troglodytes) and western lowland gorilla (Gorilla gorilla gorilla) are housed in the Primate Collection of the Department of Comparative Anatomy of the National Museum of Natural History, Paris, France (S1 Text). Humans (Homo sapiens) are housed in the Raymond A. Dart Collection of Human Skeletons at the University of the Witwatersrand, Johannesburg, South Africa (S1 Text) [70]. (ZIP) [file pone.0117905.s004.zip › X-Ray_Cortical structure of hallucal metatarsals and locomotor adaptations in hominoids/30_F_A22_Xosa_MT1L.0002.bmp]

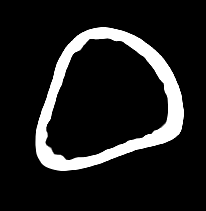

Supplement: S1 File — Individuals in central chimpanzee (Pan troglodytes troglodytes) and western lowland gorilla (Gorilla gorilla gorilla) are housed in the Primate Collection of the Department of Comparative Anatomy of the National Museum of Natural History, Paris, France (S1 Text). Humans (Homo sapiens) are housed in the Raymond A. Dart Collection of Human Skeletons at the University of the Witwatersrand, Johannesburg, South Africa (S1 Text) [70]. (ZIP) [file pone.0117905.s004.zip › X-Ray_Cortical structure of hallucal metatarsals and locomotor adaptations in hominoids/30_F_A22_Xosa_MT1L.0003.bmp]

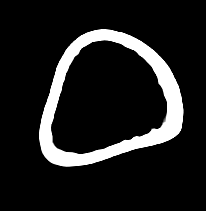

Supplement: S1 File — Individuals in central chimpanzee (Pan troglodytes troglodytes) and western lowland gorilla (Gorilla gorilla gorilla) are housed in the Primate Collection of the Department of Comparative Anatomy of the National Museum of Natural History, Paris, France (S1 Text). Humans (Homo sapiens) are housed in the Raymond A. Dart Collection of Human Skeletons at the University of the Witwatersrand, Johannesburg, South Africa (S1 Text) [70]. (ZIP) [file pone.0117905.s004.zip › X-Ray_Cortical structure of hallucal metatarsals and locomotor adaptations in hominoids/30_F_A22_Xosa_MT1L.0004.bmp]

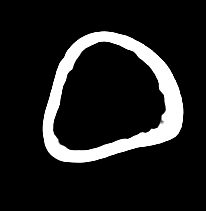

Supplement: S1 File — Individuals in central chimpanzee (Pan troglodytes troglodytes) and western lowland gorilla (Gorilla gorilla gorilla) are housed in the Primate Collection of the Department of Comparative Anatomy of the National Museum of Natural History, Paris, France (S1 Text). Humans (Homo sapiens) are housed in the Raymond A. Dart Collection of Human Skeletons at the University of the Witwatersrand, Johannesburg, South Africa (S1 Text) [70]. (ZIP) [file pone.0117905.s004.zip › X-Ray_Cortical structure of hallucal metatarsals and locomotor adaptations in hominoids/30_F_A22_Xosa_MT1L.0005.bmp]

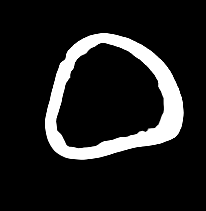

Supplement: S1 File — Individuals in central chimpanzee (Pan troglodytes troglodytes) and western lowland gorilla (Gorilla gorilla gorilla) are housed in the Primate Collection of the Department of Comparative Anatomy of the National Museum of Natural History, Paris, France (S1 Text). Humans (Homo sapiens) are housed in the Raymond A. Dart Collection of Human Skeletons at the University of the Witwatersrand, Johannesburg, South Africa (S1 Text) [70]. (ZIP) [file pone.0117905.s004.zip › X-Ray_Cortical structure of hallucal metatarsals and locomotor adaptations in hominoids/30_F_A22_Xosa_MT1L.0006.bmp]

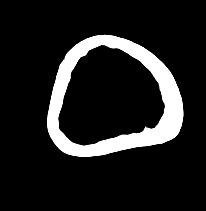

Supplement: S1 File — Individuals in central chimpanzee (Pan troglodytes troglodytes) and western lowland gorilla (Gorilla gorilla gorilla) are housed in the Primate Collection of the Department of Comparative Anatomy of the National Museum of Natural History, Paris, France (S1 Text). Humans (Homo sapiens) are housed in the Raymond A. Dart Collection of Human Skeletons at the University of the Witwatersrand, Johannesburg, South Africa (S1 Text) [70]. (ZIP) [file pone.0117905.s004.zip › X-Ray_Cortical structure of hallucal metatarsals and locomotor adaptations in hominoids/30_F_A22_Xosa_MT1L.0007.bmp]

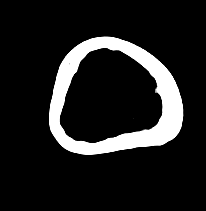

Supplement: S1 File — Individuals in central chimpanzee (Pan troglodytes troglodytes) and western lowland gorilla (Gorilla gorilla gorilla) are housed in the Primate Collection of the Department of Comparative Anatomy of the National Museum of Natural History, Paris, France (S1 Text). Humans (Homo sapiens) are housed in the Raymond A. Dart Collection of Human Skeletons at the University of the Witwatersrand, Johannesburg, South Africa (S1 Text) [70]. (ZIP) [file pone.0117905.s004.zip › X-Ray_Cortical structure of hallucal metatarsals and locomotor adaptations in hominoids/30_F_A22_Xosa_MT1L.0008.bmp]

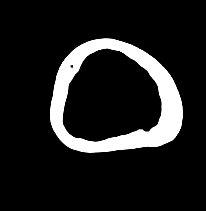

Supplement: S1 File — Individuals in central chimpanzee (Pan troglodytes troglodytes) and western lowland gorilla (Gorilla gorilla gorilla) are housed in the Primate Collection of the Department of Comparative Anatomy of the National Museum of Natural History, Paris, France (S1 Text). Humans (Homo sapiens) are housed in the Raymond A. Dart Collection of Human Skeletons at the University of the Witwatersrand, Johannesburg, South Africa (S1 Text) [70]. (ZIP) [file pone.0117905.s004.zip › X-Ray_Cortical structure of hallucal metatarsals and locomotor adaptations in hominoids/30_F_A22_Xosa_MT1L.0009.bmp]
