# Supplementary material for: Cortical Structure of Hallucal Metatarsals and Locomotor Adaptations in Hominoids
Source: PLoS One. 2015 Jan 30;10(1):e0117905. doi: 10.1371/journal.pone.0117905 (PMC4311976; doi:10.1371/journal.pone.0117905)
Supplement: S1 Text — (DOC) [file pone.0117905.s005.doc]

**S1 Text:** Sample and collection

| **Id Number** | **Taxon** |  |  | **Collection** |
| --- | --- | --- | --- | --- |
| 1901-659 | Pan |  |  | The Primate Collection of the Department of Comparative Anatomy of the National Museum of Natural History, Paris, France |
| 1923-2497 | Pan |  |  | The Primate Collection of the Department of Comparative Anatomy of the National Museum of Natural History, Paris, France |
| 1947-149 | Pan |  |  | The Primate Collection of the Department of Comparative Anatomy of the National Museum of Natural History, Paris, France |
| 1921-299 | Pan |  |  | The Primate Collection of the Department of Comparative Anatomy of the National Museum of Natural History, Paris, France |
| 1932-45 | Pan |  |  | The Primate Collection of the Department of Comparative Anatomy of the National Museum of Natural History, Paris, France |
| 1936-630 | Pan |  |  | The Primate Collection of the Department of Comparative Anatomy of the National Museum of Natural History, Paris, France |
| A8874 | Pan |  |  | The Primate Collection of the Department of Comparative Anatomy of the National Museum of Natural History, Paris, France |
| A12761 | Pan |  |  | The Primate Collection of the Department of Comparative Anatomy of the National Museum of Natural History, Paris, France |
| 1899-129 | Pan |  |  | The Primate Collection of the Department of Comparative Anatomy of the National Museum of Natural History, Paris, France |
| 1944-227 | Pan |  |  | The Primate Collection of the Department of Comparative Anatomy of the National Museum of Natural History, Paris, France |
| 1950-194 | Pan |  |  | The Primate Collection of the Department of Comparative Anatomy of the National Museum of Natural History, Paris, France |
| 1966-330 | Pan |  |  | The Primate Collection of the Department of Comparative Anatomy of the National Museum of Natural History, Paris, France |
| 1967-198bis222-3 | Pan |  |  | The Primate Collection of the Department of Comparative Anatomy of the National Museum of Natural History, Paris, France |
| 1966-332 | Pan |  |  | The Primate Collection of the Department of Comparative Anatomy of the National Museum of Natural History, Paris, France |
| 1904_125 | Gorilla |  |  | The Primate Collection of the Department of Comparative Anatomy of the National Museum of Natural History, Paris, France |
| 1943_110 | Gorilla |  |  | The Primate Collection of the Department of Comparative Anatomy of the National Museum of Natural History, Paris, France |
| 1978_89 | Gorilla |  |  | The Primate Collection of the Department of Comparative Anatomy of the National Museum of Natural History, Paris, France |
| 1856_67 | Gorilla |  |  | The Primate Collection of the Department of Comparative Anatomy of the National Museum of Natural History, Paris, France |
| 1897_276 | Gorilla |  |  | The Primate Collection of the Department of Comparative Anatomy of the National Museum of Natural History, Paris, France |
| 1912-475 | Gorilla |  |  | The Primate Collection of the Department of Comparative Anatomy of the National Museum of Natural History, Paris, France |
| 1914-98 | Gorilla |  |  | The Primate Collection of the Department of Comparative Anatomy of the National Museum of Natural History, Paris, France |
| 1928_272 | Gorilla |  |  | The Primate Collection of the Department of Comparative Anatomy of the National Museum of Natural History, Paris, France |
| 1931_657 | Gorilla |  |  | The Primate Collection of the Department of Comparative Anatomy of the National Museum of Natural History, Paris, France |
| 1933-74 | Gorilla |  |  | The Primate Collection of the Department of Comparative Anatomy of the National Museum of Natural History, Paris, France |
| 1981_020 | Gorilla |  |  | The Primate Collection of the Department of Comparative Anatomy of the National Museum of Natural History, Paris, France |
| A12747 | Gorilla |  |  | The Primate Collection of the Department of Comparative Anatomy of the National Museum of Natural History, Paris, France |
| 1914-99 | Gorilla |  |  | The Primate Collection of the Department of Comparative Anatomy of the National Museum of Natural History, Paris, France |
| 220060 | Gorilla |  |  | USNM |
| A8_Soto | Humans | M | 30 | Raymond A. Dart Collection of Human Skeletons at the University of the Witwatersrand, Johannesburg, South Africa |
| A9_Vend | Humans | M | 20 | Raymond A. Dart Collection of Human Skeletons at the University of the Witwatersrand, Johannesburg, South Africa |
| A13_Pondo | Humans | M | 30 | Raymond A. Dart Collection of Human Skeletons at the University of the Witwatersrand, Johannesburg, South Africa |
| A14_Zulu | Humans | M | 25 | Raymond A. Dart Collection of Human Skeletons at the University of the Witwatersrand, Johannesburg, South Africa |
| A17_Soto | Humans | M | 26 | Raymond A. Dart Collection of Human Skeletons at the University of the Witwatersrand, Johannesburg, South Africa |
| A163_Soto | Humans | M | 25 | Raymond A. Dart Collection of Human Skeletons at the University of the Witwatersrand, Johannesburg, South Africa |
| A170_Soto | Humans | M | 29 | Raymond A. Dart Collection of Human Skeletons at the University of the Witwatersrand, Johannesburg, South Africa |
| A22_Xosa | Humans | F | 30 | Raymond A. Dart Collection of Human Skeletons at the University of the Witwatersrand, Johannesburg, South Africa |
| A209_Soto | Humans | F | 25 | Raymond A. Dart Collection of Human Skeletons at the University of the Witwatersrand, Johannesburg, South Africa |
| A218_Soto | Humans | F | 25 | Raymond A. Dart Collection of Human Skeletons at the University of the Witwatersrand, Johannesburg, South Africa |
| A458_Soto | Humans | F | 24 | Raymond A. Dart Collection of Human Skeletons at the University of the Witwatersrand, Johannesburg, South Africa |
| A883_Soto | Humans | F | 27 | Raymond A. Dart Collection of Human Skeletons at the University of the Witwatersrand, Johannesburg, South Africa |
| A1285_Soto | Humans | F | 29 | Raymond A. Dart Collection of Human Skeletons at the University of the Witwatersrand, Johannesburg, South Africa |
| A3060_Soto | Humans | F | 29 | Raymond A. Dart Collection of Human Skeletons at the University of the Witwatersrand, Johannesburg, South Africa |
| A10_Xosa | Humans | M | 45 | Raymond A. Dart Collection of Human Skeletons at the University of the Witwatersrand, Johannesburg, South Africa |
