# Supplementary material for: Cortical Structure of Hallucal Metatarsals and Locomotor Adaptations in Hominoids
Source: PLoS One. 2015 Jan 30;10(1):e0117905. doi: 10.1371/journal.pone.0117905 (PMC4311976; doi:10.1371/journal.pone.0117905)
Supplement: S2 Text — (DOC) [file pone.0117905.s006.doc]

**S2 Text**

Methodology for Extracting Contours of Cross Sections via Kernel Smoothing

The scanning equipment derived data are a raster of 8-bit cells forming a gray-scale image of a single cross section. For a given image, let $p_{ij}^{*}$ represent the 8-bit value for the pixel in the *i*^th^ row and *j*^th^ column of the raster (i.e. $0\leq p_{ij}^{*}\leq255$) and $p_{ij}=p_{ij}^{*}/255$ represent the gray-scale value for that same (*i*, *j*)^th^ pixel (i.e. $0\leq p_{ij}\leq1$). The goal is to use this image raster information to (digitally) obtain measurements of variables of interest (e.g. cortical thickness).

In order to extract measurements of cortical thickness a mathematical representation of the (analog) contour defining the inner and outer edges of the cortical bone needs to first be derived from the (digital) raster data above. Typically, for cross sections of bone, the true contour would be expected to be a smooth, continuous function (at least at the resolutions available for scanning). However, if the digital raster data is pre-processed with a binary-threshold (for example, set $p_{ij}^{*}=0$whenever $p_{ij}^{*}<128$ and $p_{ij}^{*}=255$ otherwise, or equivalently set $p_{ij}=0$whenever $p_{ij}<128/255\approx0.5$ and $p_{ij}=1$ otherwise), then the implied contour edge will not be smooth if it is taken to follow the boundaries of transition pixels (due to the square corners and straight edges of the digital pixels). Thresholding in this way may be followed by an elliptical Fourier analysis (EFA) (e.g. Kuhl and Giardina (1982)) to extract the first few Fourier components that yield a smooth curve to represent the underlying contour. A choice of the degree of smoothing is required (as would be required by any alternative approach too); for EFA the amount of smoothing is related to the number of Fourier components retained – something probably most often chosen subjectively and for convenience. A slight drawback to any method based on a binary-threshold first step is that some area/volume-averaged information may be discarded (if the pixel value is below the threshold, even if only slightly) or a pixel may be given disproportionate emphasis (if the pixel value is above the threshold, even if only slightly).

In an attempt to account for the secondary information available in area/volume-averaged pixels as well as obtain a mathematical representation of a smooth, continuous contour, a kernel-based approach might be used instead. To do this, a kernel function, $K_{h}\left(u,v\right)$, is positioned at the center of every pixel and weighted by the value of that pixel. The aggregate of all these kernels across the entire raster creates a three-dimensional surface, $S\left(x,y\right)$, whose height at any point (not just at pixel centers) is related to the value of pixels in the neighbourhood of that point. Mathematically,

where $K_{h}\left(u,v\right)$ is any valid kernel function with smoothing parameter *h*, and *C* is a (arbitrary) scaling constant, since we’re only interested in the height of this surface. In two dimensions, the kernel (i.e. ignoring any scaling constant) of any bivariate probability distribution is a valid kernel function. One such candidate is the spherical (i.e. zero correlation) bivariate normal density function given by

$K_{h}\left(u,v\right)=\frac{1}{2\pi h}\exp\left\{-\frac{1}{2h}\left[u^{2}+v^{2}\right]\right\}$ with $-\infty<u,v<\infty$.

As a small example, Figure S4 shows a gray-scale raster image (left) and the same viewed as a 3D histogram of 8-bit pixel values (right), while S5 Figure shows the inferred contours for this image using a bivariate normal kernel function with *h* = 1/2.

It now remains to determine which contour to use (e.g. in left plot of S5 Figure) as the representative for the edge of the cross section. This could be done “by eye” (similar to choosing the number of EFA components to retain) or else it could be done by minimizing some measure (e.g. L_1_ or L_2_ norm) of the difference between the original raster pixel values and the (area/volume-averaged) pixel values that are obtained when partitioning the implied cross section on the same grid as the original raster i.e. choose a contour that would “as close as possible” recover the original area/volume-averaged pixel-values.

Any attempt at optimizing the extracted contour necessarily incurs additional computational expense. A “quick-and-dirty” contour could be extracted by choosing the contour whose height is the same as the height of the kernel on the midpoints of the four edges of the pixel square that it is placed within. This seems (empirically) to be somewhat equivalent to using a (very low parameter in a) binary-threshold.

For example, the two plots in S6 Figure show the same real digital cross section: the left plot shows the inferred contour using the coarse “edge-height” choice mentioned above while the right plot shows the contour selected by minimizing the difference between actual versus inferred pixel values in L_2_ norm (i.e. squared error).

The differences are subtle but it is clear the left choice incorrectly infers a slightly “thicker” cross section than that on the right, which visually seems to more faithfully adhere to the apparent edge of the real digital cross section.

**S4, S5, S6 Figures for S2 Text**

**S4 Figure**. Subsection of a gray-scale raster image (left) and the same viewed as a 3D histogram of 8-bit pixel values (right).

**S5 Figure**. Kernel-inferred contours (left) and the 3D kernel surface *S*(*x*, *y*) (right) for the same gray-scale raster image example in S2 Figure. [Note: there are some “edge” effects in this small example on the boundaries of the square raster.]

**S6 Figure**. Contour using the coarse “edge-height” choice (left). Contour selected by minimizing the difference between actual versus inferred pixel values in L_2_ norm (i.e. squared error) (right). The cyan lines are the inferred cross section boundary and the red dot corresponds to the centroid of the cross section.
